# Single-Cell Analysis of Sex and Gender Differences in the Human Brain During Development and Disease

**DOI:** 10.1007/s10571-025-01536-2

**Published:** 2025-02-27

**Authors:** Aura Zelco, Anagha Joshi

**Affiliations:** 1 Department of Clinical Science, Computational Biology Unit, University of Bergen, Bergen, Norway; 2 Department of Biotechnology, Bhupat and Jyoti Mehta School of Biosciences, IIT Madras, Chennai, India

**Keywords:** Sex and gender differences, Human brain, Transcriptome, Single-nucleus RNA-seq, Brain development, Data integration

## Abstract

Sex and gender (SG) differences in the human brain are of interest to society and science as numerous processes are impacted by them, including brain development, behavior, and diseases. By collecting publicly available single-cell data from the *in-utero* to elderly age in healthy, Alzheimer’s disease and multiple sclerosis samples, we identified and characterized SG-biased genes in ten brain cell types across 9 age and disease groups. Sex and gender differences in the transcriptome were present throughout the lifespan and across all cell types. Although there was limited overlap among SG-biased genes across different age and disease groups, we observed significant functional overlap. Female-biased genes are consistently enriched for brain-related processes, while male-biased genes are enriched for metabolic pathways. Additionally, mitochondrial genes showed a consistent female bias across cell types. We also found that androgen response elements (not estrogen) were significantly enriched in both male- and female-biased genes, and thymosin hormone targets being consistently enriched only in male-biased genes. We systematically characterised SG differences in brain development and brain-related disorders at a single-cell level, by analysing a total of publicly available 419,885 single nuclei from 161 human brain samples (72 females, 89 males). The significant enrichment of androgen (not estrogen) response elements in both male- and female-biased genes suggests that androgens are important regulators likely establishing these SG differences. Finally, we provide full characterization of SG-biased genes at different thresholds for the scientific community as a web resource.

**Graphical Abstract:**

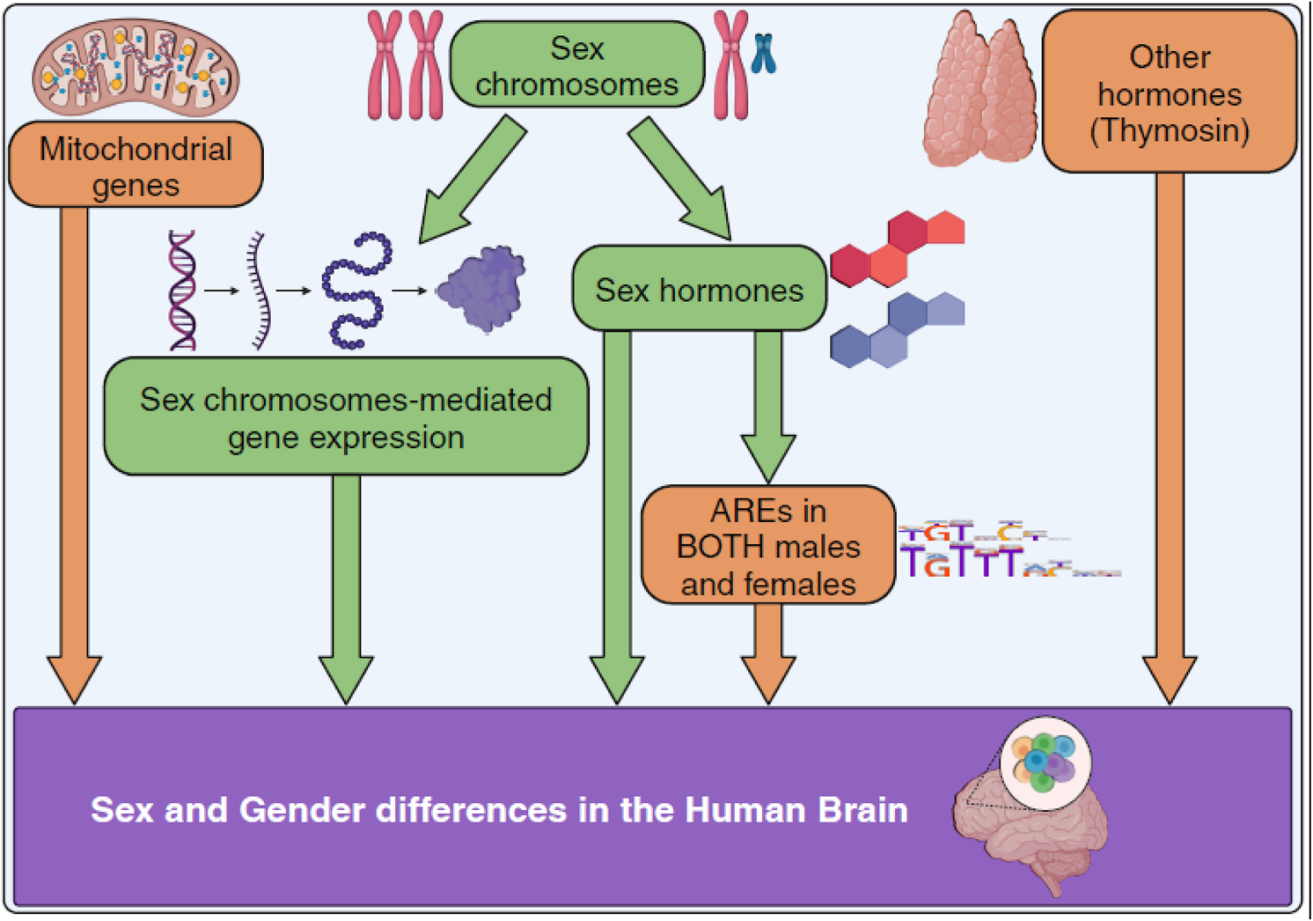
Summary of the sex and gender differences found in the human cortex transcriptome. After stratification by cell type and developmental stage, we identified SG-biased genes in human cortex transcriptome. Comprehensive analysis of SG-biased genes resulted in main findings; the female-biased mitochondrial gene up-regulation, the presence of ARE binding sites at the SG-biased DEGs for both sexes, and the enrichment of hormone targets other than sex hormones, such as thymosin. Our findings (highlighted in orange) further extend the traditional model of sex and gender differences in the human brain (highlighted in green). The ARE motif (split in two lines) was obtained via the HOmo sapiens COmprehensive MOdel COllection (version 12). *ARE* androgen response elements.

**Supplementary Information:**

The online version contains supplementary material available at 10.1007/s10571-025-01536-2.

## Background

The existence, scope, and importance of sex differences in the human brain are contentious issues in both the scientific community and the public. In scientific literature, the terminology for sex and gender is often used confusingly. Although sex is increasingly considered a biological variable in scientific investigation, phenotypes often depend on both sex and gender. In our study, we define gender as the influence of socio-cultural distinctions and identity, and sex as a dimorphic state based on the presence of X and Y chromosomes in DNA. While we acknowledge that this definition is somewhat limited and does not encompass the diversity of sexes and genders, these cases are beyond the scope of our study. Importantly, sex and gender differences in the brain might originate from either the sex chromosomes, the individual’s gender identity, or both. Therefore, we will refer to them as sex and gender (SG) differences.

Differences between sexes and genders evolve dynamically through developmental processes that impact numerous phenotypes. SG differences in the human brain have implications in a variety of processes, including brain development, behavior, and the presentation, prevalence, and therapeutic response of disease. The brain is not sexually dimorphic, but there are many documented anatomical (Buffery 1971; Ingalhalikar et al. 2014; Kurth et al. 2021; Lewis et al. 2022; Fuhrmann et al. 2022; Neufang et al. 2009) and behavioural (Altemus et al. 2014; Beking et al. 2018; Peper et al. 2020) differences between the sexes and genders, including some controversies (e.g. DeCasien et al. 2022; Eliot et al. 2021; Williams et al. 2021). Male and female SG differences in behaviour emerge at early age, further widening during adolescence (Kaczkurkin et al. 2019). These differences have an impact on a variety of physiological processes, including the regulation of the immune system, the response to injury or disease, and the ability to produce and respond to hormones (Altemus et al. 2014; Arain et al. 2013; Bao and Swaab 2011; Clayton 2016; Hanamsagar and Bilbo 2016; Hines 2020; Turner et al. 2019). These differences in the brain are influenced by hormones, genetics, and environmental factors. Hormones regulate growth, maturation, and behavior, with varying levels between males and females affecting neuron development and neural circuit formation. Higher estrogen levels in females are linked to enhanced memory and emotional processing, while higher testosterone levels in males are associated with spatial ability and aggressive behavior (Zaidi 2010; Sherwin 2003). Despite the presence of consistent large behavioural sex and gender differences, the molecular or structural correlates of these differences has so far led to a limited success (van Eijk et al. 2021).

Efforts by large consortia and the reduced cost of sequencing technologies have made genome-wide expression data accessible across tissues from hundreds of individuals. This data has enabled the identification of sex and gender (SG) differences at the whole genome level in various organs and tissues, including the brain (Oliva et al. 2020; Gershoni and Pietrokovski 2017; Wapeesittipan and Joshi 2023). Accordingly the studies investigating the role of SG differences in brain have dramatically increased in recent years (Fig. S1), however they tend to focus on a specific disease, cell type or brain region to explore SG differences (Rosenkrantz et al. 2019; Rebuli et al. 2016; Belonwu et al. 2022). Previous findings indicate that there are small but consistent SG differences in several brain regions, including the cortex (Oliva et al. 2020). In addition, SG biases in transcriptome and gene regulatory networks have been observed in the second trimester of pregnancy, very early in the development process (O’Brien et al. 2019; de Toledo et al. 2022). During brain development, maturation, and neurological diseases, differences in the number, distribution, and activity of particular cells, such as neurons and immune cells, have been observed between males and females (Altemus et al. 2014; Arain et al. 2013; Bao and Swaab 2011; Clayton 2016; Hanamsagar and Bilbo 2016; Hines 2020; Turner et al. 2019). However, inconsistencies were noted in cell types with sex and gender biases (Wapeesittipan and Joshi 2023). This might be partly because these studies rely on bulk RNA-seq technology, limiting the characterization of cell type-specific SG differences in a complex organ such as the brain. Therefore, studies are needed to fully understand the nature and extent of sex differences at a single-cell level. Despite many recent studies have generated single-cell/nucleus data in the human brain at across ages and healthy/disease statuses (e.g. Velmeshev et al. 2023; Lau et al. 2020; Morabito et al. 2021; Schirmer et al. 2019; Eze et al. 2021; Nowakowski et al. 2017), they do not specifically explore SG differences.

Importantly, brain region and age contribute more to the global differences in gene expression than sex and gender (Kang et al. 2011). It is vitally important to obtain age-stratified samples to identify sex and gender differences. When the spatio-temporal dynamics of the human brain transcriptome were explored in 16 brain regions, from embryonic development to late adulthood, it demonstrated that developmentally and spatially regulated differences in gene- and exon-level expression exist between male and female brains, albeit using bulk RNA-seq samples (Kang et al. 2011). Thus, characterization of the SG differences across brain cell types throughout the life span is still missing. Furthermore, SG affect many regions of brain, therefore brain region choice is of high relevance. The cortex is one of the brain regions where most of anatomical SG differences are found (Arain et al. 2013; Lombardo et al. 2012b; Neufang et al. 2009; Fuhrmann et al. 2022), and with the most available datasets for single-cell analysis. In this study, we therefore set out to investigate and characterize in depth the SG differences during the brain maturation, from fetal to adult life, using publicly available human single-nucleus RNA-sequencing datasets from human cortical region. These datasets included also patients diagnosed with Alzheimer’s disease and multiple sclerosis, to investigate SG differences in cognitive and neurological diseases.

## Methods

### Data and Code Availability

The datasets included in this analysis were obtained from the following publicly available databases: University of California Santa Cruz (UCSC) Cell Browser (Speir et al. 2021), and DISCO v1.0 (Li et al. 2022). Both databases provide publicly available datasets, and thus were not generated specifically for this study. We found only four projects (Velmeshev et al. 2023; Lau et al. 2020; Morabito et al. 2021; Schirmer et al. 2019) providing single-cell data in human cortex through lifespan. Other studies were explored but then excluded for reasons such as incomplete metadata (sex, age, cell type annotation, etc) and/or insufficient number of samples, such as the 4–10 years Velmeshev dataset (Velmeshev et al. 2023). The included projects were chosen since they had enough male and female samples covering a large part of the human life span, including the fetal and neonatal period; adult datasets were also included to investigate temporal changes between the sexes at later stage in life as well as some disease states. Furthermore, we focused on cortical samples, for biological and sample availability reasons. Hereafter, dataset refers to samples from a given age and disease group, thus the included studies were split in multiple datasets, either by age or disease status. The scripts and code used to run the whole analysis, including figures present in this article, can be found at https://github.com/aurazelco/SGDifferencesHumanBrain.

### Single-Nucleus Data Sources

Summary information of the datasets included in this analysis are outlined in Tables  1,  2. Briefly, all studies included in this publication utilised 10X Genomics 3’ library kits v3 (Table 2), with similar combinations of enzymatic and mechanical dissociation on frozen human cortical samples. Only Velmeshev et al. have mentioned an average sequencing depth 60,000 reads/nucleus. Schirmer et al. specified that they sequenced controls and MS samples together to avoid batch effects, while Morabito et al. mentioned applying batch correction. Velmeshev et al. and Lau et al. did not detect any batch effect. Schirmer et al. sequenced the samples on an Illumina HiSeq 2500 machine, while the other groups utilised a NovaSeq 6000 sequencer. All but Morabito et al. specified that the mapping was done against the ENSEMBL GRCh38 human genome assembly. Summary figures of gene counts per cell (nFeature_RNA) and total molecules within a cell (nCount_RNA) are reported for comparison (Fig. S2). Despite differences in these parameters, each data source contained enough high quality data to continue with the downstream analysis.

**Table 1:** Summary of data from source datasets

Study ID	Number of samples	Sequencing method	Sequencing depth
Source	Study	GEO accession number	Publications
DISCO	Lau et al.	GSE157827	https://www.pnas.org/doi/10.1073/pnas.2008762117
DISCO	Morabito et al.	GSE174367	https://www.nature.com/articles/s41588-021-00894-z
DISCO	Schirmer et al.	PRJNA544731	https://www.nature.com/articles/s41586-019-1404-z
UCSC cell browser	Velmeshev et al.	–	https://www.science.org/doi/10.1126/science.adf0834

The information in this table is directly quoted from the corresponding publications, and some details are missing because they cannot be found in the original paper

### Single-Nucleus Data Processing

The main data analysis was performed using R (4.1.1, 4.2.1) (R Core Team 2020; RStudio Team 2019), with the Seurat package (4.2.0, 4.3.0) (Hao et al. 2021). Multiple versions are due to running the analysis on different machines; Apple MacBook Air M1 2020 macOS BigSur v11.6.8 (4.2.0) and OracleLinux v8.7 Fedora (4.3.0).

A standard single-cell data analysis protocol was used for the Velmeshev data, removing cells with a percentage of mitochondrial genes higher than 5% and with too few (less than 200) or too many (2500) genes. This was followed by normalization, scaling and clustering according to Seurat recommendations. We selected only cortical samples in all datasets. The sex of the samples was verified by checking the expression of XIST, a X chromosome gene mainly expressed in females (Fig. S5). There was no expression of XIST or other X and Y genes in the second trimester dataset, thus the sex metadata therefore could not be confirmed in this case.

**Table 2:** Summary of data from source datasets

Study ID	Number of samples	Sequencing method	Sequencing depth
GSE157827	7 F, 11 M	Single-nucleus RNA-seq, chromium single cell 3’ library kit v3	No information
GSE174367	9 F, 10 M	Single-nucleus RNA-seq, 10x chromium single cell 3’ v3 platform	No information
PRJNA544731	10 F, 9 M	Single-nucleus RNA-seq, 10x genomics single-cell 3’ system	No information
Velmeshev	43 F, 48 M	Single-nucleus RNA-seq, 10X genomics single-cell 3’ system	Average depth 60,000 reads/nucleus

The information in this table is directly quoted from the corresponding publications, and some details are missing because they cannot be found in the original paper

The Velmeshev single-nucleus study (Velmeshev et al. 2023) provided data for the most datasets, divided by age range; namely, second trimester, third trimester, 0–1 years, 1–2 years, 2–4 years, 4–10 years, 10–20 years and Adults. The 4–10 years dataset was excluded as it had only one female sample (Fig. S3). Each dataset was analyzed individually. The one instance where all datasets were visualized together was to confirm that our cell type distribution in the Uniform Manifold Approximation and Projection (UMAP) plot was similar to the original paper (data not shown).

From the DISCO database (Li et al. 2022), we removed from the brain v1.0 database the projects which did not have metadata about the age, and kept only the samples from the cortex. This resulted in three single-nucleus RNA-seq projects with both female and male samples in the final selection: GSE157827 (Lau et al. 2020), GSE174367 (Morabito et al. 2021) and PRJNA544731 (Schirmer et al. 2019). The DISCO projects also included samples from patients suffering from Alzheimer’s disease (AD) (Lau et al. 2020; Morabito et al. 2021) and multiple sclerosis (MS) (Schirmer et al. 2019). No modification was done to the original clustering, which were visually confirmed using the UMAP as in the original version from DISCO (data not shown).

Consistent cell annotation across datasets was one of the challenging steps of the analysis. When analyzing the individual datasets, the original cell annotation was kept as present in the metadata. When the results from the differential expression gene analysis were compared and integrated, the different annotations were organized manually (Supplementary file 1 for details).

### Differential Expression Gene Analysis

The Seurat package was used to calculate the differentially expressed genes (DEGs), using the default FindMarkers parameters (logfc.threshold 0.25, min.pct 0.1). Briefly, in each dataset, each cell type that had at least 100 cells for each of the female and male sex was analyzed, thus obtaining two lists of DEGs (one per sex) in each cell type. We selected only the up-regulated genes in each sex. We therefore stratified the samples by biological sex (female/male), however we used the SG-biased genes terminology because we cannot know for sure the source of the differential expression; sex, gender or both. These SG-biased DEG lists were then filtered further, so that only the genes with fold change with absolute values of at least 1.2 and adjusted *p*-value (Bonferroni correction) of 0.05 or lower were kept. Furthermore, only cell types with more than 10 DEGs after filtering were included for further analysis. For each dataset aforementioned in section “Single-Nucleus Data Processing”, the different ages and disease conditions were analyzed separately, including separating the three projects in the DISCO dataset.

### Down-Sampling Analysis

To investigate the consistency of our differential analysis, we performed down-sampling on one of the largest cell populations, excitatory neurons, across three different datasets (Velmeshev 3rd trimester, GSE157827 Healthy and Alzheimer’s disease), conducting multiple rounds of down-sampling. Specifically, we sampled 3, 5, 10, 50, 100, 250, and 500 cells for each dataset and SG, repeating the process 10 times. We then calculated the SG-biased DEGs for these subsets as previously described. Subsequently, we pair-wise compared the common significant SG-biased DEGs among the same down-sampling sizes to determine the distribution of common genes between any two random samplings. We excluded the smaller sampling sizes (*n* $=$ 3, 5, and 10) as they yielded no significant SG-biased DEGs.

### Mitochondrial Genes

We investigated the presence of mitochondrial genes in the SG-biased genes by selecting genes which names started with “MT-”. We also included “TIMM” and “TOMM” in the search. Since a recent study implicated a female bias in the expression of Krebs or tricarboxylic acid (TCA) cycle, in connection with mitochondrial activity, we added also these enzymes from the following source (https://maayanlab.cloud/Harmonizome/gene_set/TCA+cycle/PANTHER+Pathways). We included “XIST” as a control for female-biased expression.

### Cellular Compartment Enrichment

Using the Cell Atlas (Thul et al. 2017), we mapped the SG-biased genes according to their cellular compartment. The Cell Atlas, compiled using immunofluorescence microscopy to map proteins, was used to map the corresponding protein products of genes. For each dataset, the number of SG-biased genes were for each sex and cell type found in each cellular location were used to calculate enrichment in each cellular compartment using hyper-geometric test in R (stats package, 4.1.1). The enrichment was considered significant when *p*-value < 0.05.

### Cell Type Markers

We evaluated the cell type specificity of the SG-biased genes in each sex and dataset, using a reference (McKenzie et al. 2018). Briefly, we calculated the percentage of known markers from five brain populations (astrocytes, neurons, endothelial cells, microglia and oligodendrocytes) in the each cell type, for each dataset in each sex.

### Functional, Pathological and Transcription Enrichment

We used the SG-biased genes to study functional enrichment, in each cell type across all datasets and for each dataset across all cell types, both divided by sex. For this analysis we used the following packages: clusterProfiler v4.2.2 (Yu et al. 2012; Wu et al. 2021), disgenet2r v0.99.2 (Pinero et al. 2020), enrichR v3.1 (Chen et al. 2013; Kuleshov et al. 2016). We used clusterProfiler to investigate the enrichment for Gene Ontology (GO) of biological processes (BP), and diseases from the Disease Ontology (DO) and DisGeNet. disgenet2r was also used to obtain the enrichment using the CURATED DisGeNet database, while enrichR was used to compare pathways from Kyoto Encyclopedia of Genes and Genomes (KEGG), GWAS Catalog 2019 for SNP enrichment, and the drug enrichment using DSigDB for drug-related enrichment. Additionally, we also investigated the enrichment of transcription factor bind sites using the TRANSFAC and JASPAR PWMs database in enrichR. Only the top 5 significant terms were displayed in the plots for the comparison across the datasets or the cell types. All adjusted *p*-values from the enrichment analyses were obtained by running Fisher’s exact tests with the Benjamini–Hochberg (BH), also knows as false discovery rate (FDR), correction. The enrichment was considered significant when *p*-value < 0.05.

Additionally, we also investigated the presence of known autism-related genes using the SFARI database (https://gene.sfari.org/database/human-gene/) (Banerjee-Basu and Packer 2010). We calculated the hyper-geometric enrichment for each dataset according to SFARI nomenclature using stats (4.1.1) (R Core Team 2020; RStudio Team 2019). The enrichment was considered significant when *p*-value < 0.05.

### Neuropsychiatric Diseases Enrichment

We investigated whether genes known to be associated with neuropsychiatric diseases (Chlamydas et al. 2022) were present in the SG DEG lists, and generated a heatmap to display the results.

### Brain Region Specificity

We checked whether our SG-biased genes were expressed specifically in the cortex, or also in other brain regions. We extracted the top 500 sex-biased DEGs from all the brain regions included in a bulk RNA-seq study (Oliva et al. 2020), namely the cortex, basal ganglia, hypothalamus, amygdala, hippocampus, cerebellum, substantia nigra, spinal cord and pituitary gland. We then calculated the enrichment through hyper-geometric distribution from stats (4.1.1) (R Core Team 2020; RStudio Team 2019) and the significance was reached when *p*-value < 0.05.

### Sex Chromosomes Enrichment Analysis

The SG-biased genes were mapped to the genome with the use of the biomaRt package (2.50.3, (Durinck et al. 2005, 2009)), and enrichment of the X or Y chromosome compared to the autosomal chromosomes was calculated using the hyper-geometric test from stats (4.1.1) (R Core Team 2020; RStudio Team 2019). An enrichment was considered significant when the *p*-value < 0.05. The *p*-values were averaged to obtain one value for each cell type when comparing the datasets.

### Hormone Targets Enrichment

We next investigated the enrichment for hormone targets in the SG-biased genes by using data for 53 hormones and their respective targets from a public dataset, Hormone-Gene version 1 (Jadhav et al. 2022), available at https://github.com/BIRDSgroup/BioEmbedS. Some hormones had less than 10 targets and were therefore excluded from the enrichment. We calculated the enrichment through hyper-geometric distribution from stats (4.1.1) (R Core Team 2020; RStudio Team 2019). The enrichment was considered significant when *p*-value < 0.05 and if the hormone targets were found in more than one SG-biased list.

### Sex Hormones Response Element Analysis

We obtained list of genes with presence of androgen or estrogen response elements (AREs and EREs respectively) from previous published data (Wilson et al. 2016; Bourdeau et al. 2004). We calculated the number of genes among the SG-biased genes with ERE and ARE sites, and then represented the results as percentages of the SG-biased genes, while the genes with no RE sites labelled as “None”. The percentages for ARE sites were further divided according to whether the genes had a full, half or both half and full sites for AREs. A previous bulk RNA-seq study (Oliva et al. 2020) was used to check the percentage of ARE and ERE binding sites in the top 500 sex-biased tissue-specific genes (44 tissues and organs in total).

## Results

### An Atlas of Cell Type Specific SG-Biased Genes in Human Brain Through Life Stages and Disease

To identify and characterize SG-biased gene expression in human brain at a cellular level, we collected publicly available large single-nucleus RNA sequencing data from 419,885 nuclei (after filtering) from 161 human brain samples (72 females, 89 males) collected during entire human lifespan and disease from studies including both males and females (Figs. S3, S4). We generated consistent annotation of the cell types across studies (Supplementary file 1 for details) and defined 10 major cell types, namely dorsal and ventral progenitors, interneurons, excitatory neurons, astrocytes, T cells, microglia, endothelial cells, oligodendrocytes, oligodendrocyte precursors (OPCs), vascular cells. The data was grouped into 10 human life stages or age groups, namely second trimester, third trimester, 0–1 years, 1–2 years, 2–4 years, 10–20 years and adults (4 datasets). The diseases included were Alzheimer’s disease (AD, 2 datasets) and multiple sclerosis (MS, 1 dataset). We validated the sex of the samples from the metadata using X chromosome gene expression (Fig. S5). We stratified the samples by biological sex (female/male); however, we will henceforth use the sex and gender (SG) terminology because the exact source of the differential expression is uncertain: sex, gender, or both. The number of cells varied not only between sexes within the same dataset but also across different datasets (Figs. S4,  S6). To guarantee robust results, we used a threshold of minimum 100 cells in each cell type/sex in each dataset. Thus, some cell types were not further analyzed in some or all datasets e.g. T cells (Fig. S6). 10 major cell types; not all were present in all datasets, including the physiological absence of certain developmental cell types in later ages (e.g. dorsal and ventral progenitors) were therefore analysed further.

**Fig. 1:**
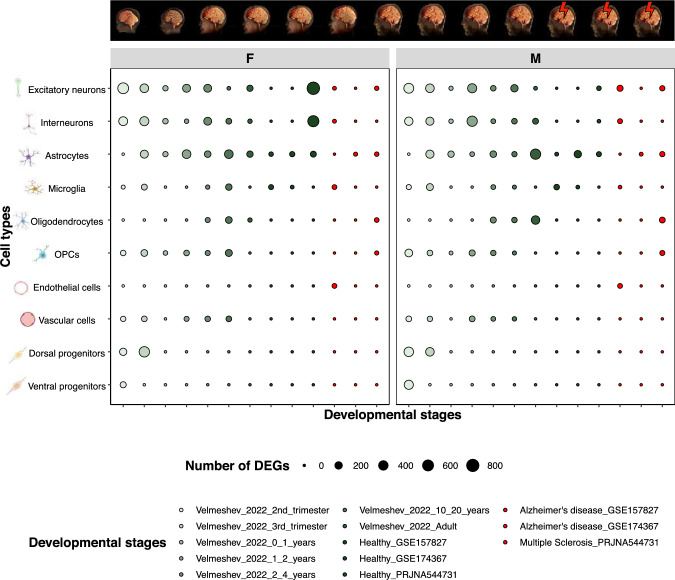
SG-biased genes are present in multiple brain cell types, and across multiple ages and pathologies. We confirmed the presence of SG-biased genes across cell types and datasets in both sexes but the number of SG-biased DEGs varied greatly. The dot size indicates the number of SG-biased DEGs found in each cell type and dataset. The brain images, indicating the ages included in this study, were modified from (Konkel 2018)

We obtained differentially expressed genes (DEGs) between males and females, henceforth called SG-biased genes using default parameters from Seurat and generated a total of 260 cell type-specific SG-biased gene lists in human brain through life stages and disease. We further combined them in 166 gene lists by merging sub-cell types into one consistent cell type annotation. The number of DEGs varied greatly across the datasets (Fig. 1) with the number of cells in each dataset influencing greatly the number of DEGs (Fig. S7). No major differences in SG-biased genes were noted between males and females within a dataset with some exceptions e.g. excitatory neurons in the healthy adults and brain pathologies.

To assess the robustness of SG-biased genes, we conducted multiple rounds of down-sampling on excitatory neurons (a cell type with large number of single cell data available) from three datasets (Velmeshev 3rd trimester, GSE157827 Healthy and Alzheimer’s disease), sampling 3, 5, 10, 50, 100, 250, and 500 cells per dataset and SG, repeating this ten times for each group. We calculated the SG-biased DEGs for these subsets using the same procedure described above and compared the common significant SG-biased DEGs among the same down-sampling sizes to determine the distribution of common genes. Smaller sampling sizes (3, 5, and 10) were excluded as they yielded no significant SG-biased DEGs. There was significant overlap between SG-biased genes across multiple down-sampling runs (only shown for Velmeshev 3rd trimester, Fig. S16). Larger sample sizes increased the number of common genes, justifying the inclusion of all available cells for each cell type in every developmental or disease group.

### Mitochondrial Genes Show a Female-Biased Gene Expression

**Fig. 2:**
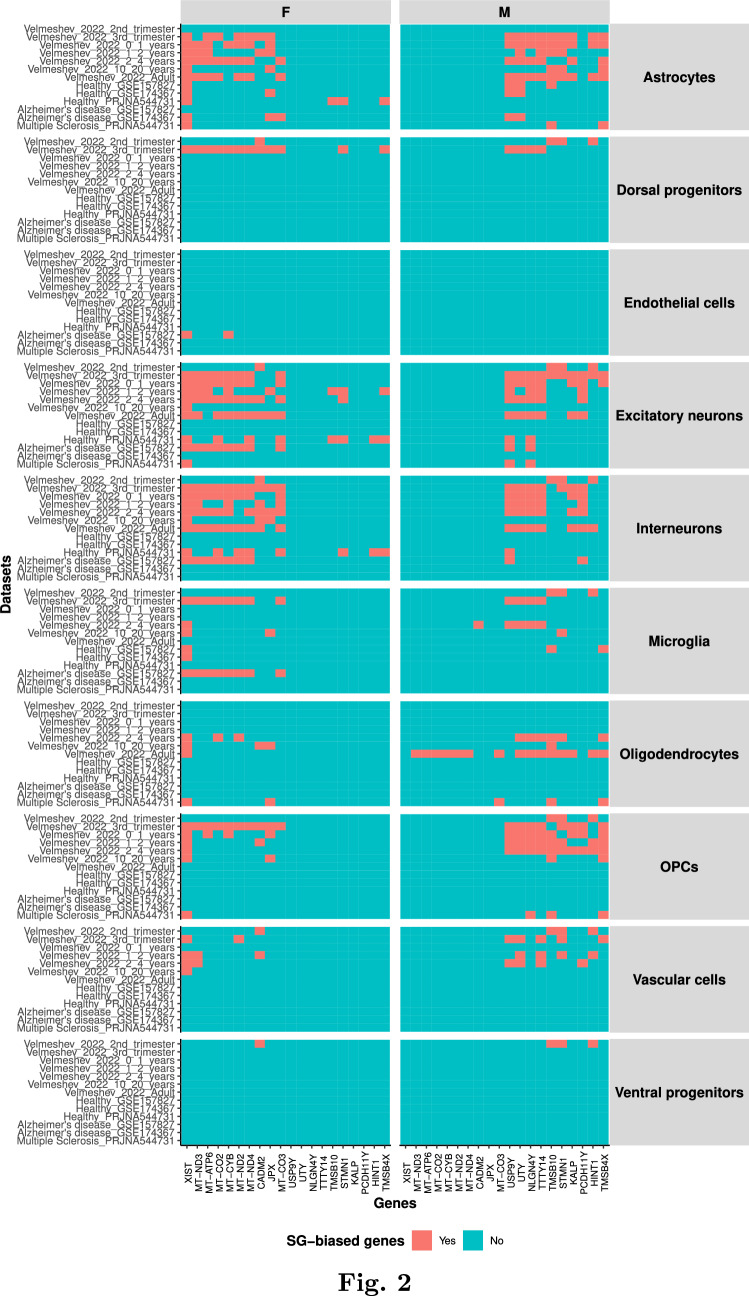
The most different SG-biased genes between sexes are mainly X- and Y-linked genes, with some exceptions. The heatmap displays the presence of the 20 most different genes between sexes (10 per sex) in SG-biased genes lists from each cell type and dataset. Most of the genes belong to either the X or Y chromosome. Notably, mitochondrial genes are among those uniquely expressed by females. Presence indicates whether the gene is found in each specific dataset-sex-cell type combination

We firstly explored the genes highly and consistently SG-biased across datasets, both for females and males (Fig. 2). As expected, the gene with the highest sex and gender (SG) difference in expression was XIST. Other female-biased SG genes included the X chromosome gene JPX, mitochondrial genes, and the autosomal gene CADM2 (chromosome 3). Similarly, genes located on the Y chromosome were predominantly expressed in males. Additionally, three male-biased SG genes (TMSB10, HINT1, STMN1) are located on autosomal chromosomes. Interestingly, TMSB4X, an X-linked gene and a target of thymosin (a non-sex hormone), is similar to TMSB10.

MT genes generally exhibit higher expression levels in females across different cell types and age groups, with some exceptions (Fig.  S8). Specifically, mitochondrial ribosomal genes like MT-RNR1 and MT-RNR2 show varied expression in female cells. Astrocytes, excitatory neurons, and interneurons consistently display increased MT gene expression in females. Conversely, MT-ATP8, which encodes ATP Synthase F0 Subunit 8, is the only mitochondrial gene with male-biased expression in excitatory neurons within the MS dataset and is not found in the female-biased gene list.

Genes functioning in mitochondria are encoded by both nuclear and mitochondrial DNA. nuclear genes associated with the Translocase Of Inner Mitochondrial Membrane/Translocase Of Outer Mitochondrial Membrane also exhibit differential expression patterns (Fig. S8). Previous studies have indicated a female bias in mitochondrial activity, with enzymes involved in the tricarboxylic acid (TCA) cycle exhibiting sex-biased gene expression. Mitochondrial complex II, which participates in both the respiratory chain and the TCA cycle using succinate as a substrate, is a key example (Lee et al. 2022). Most of the enzymes and proteins required for the TCA cycle are encoded by the nuclear DNA and then imported to mitochondria. We therefore investigated whether TCA enzymes (https://maayanlab.cloud/Harmonizome/gene_set/TCA+cycle/PANTHER+Pathways) showed SG-biased expression and found no such bias (Fig. S8).

In the down-sampling analysis mentioned earlier, many genes associated with mitochondrial function were consistently identified across various down-sampling sizes and repetitions. Notably, among genes that were found consistently SG-biased across all down-sampling runs: XIST, MT-ND4 (female-biased DEGs) and USP9Y, DDX3Y, NDUFS5 (male-biased DEGs), had a mitochondrial gene.

To gain further confidence on the female-bias of mitochondrial genes, we searched other publicly available single cell datasets in brain. Using the Single Cell Portal from the Broad Institute, we found differences in the expression of mitochondrial genes between female and male samples of human dopamine neurons from Parkinson’s disease patients (Kamath et al. 2022), fully supporting our findings (Fig. S9).

We further systematically explored whether SG-biased genes were preferentially located in specific sub-cellular locations. We obtained sub-cellular localization data of protein-coding genes from the Cell Atlas (Thul et al. 2017). Interestingly, the protein products of male-biased genes were enriched in endoplasmic reticulum in males, especially in non-neuronal populations (Fig. S10). Most corresponding proteins of the SG-biased genes were present in the cytosol, and we noted very little difference in sub-cellular location distribution between females and males, and across the datasets, except for the mitochondria, more frequent in females than in males (Fig. S10).

### SG-Biased Genes are Largely Cell Type Specific

We checked whether the SG-biased genes were shared across cell types in each dataset by calculating the overlap of gene lists across cell types. We noted that most SG-biased genes were highly specific to each cell type (Figs. S11, S12, S13, S14, S15). However, across datasets, SG-biased genes showed a higher overlap with cell types closely associated in developmental lineage, as expected. Specifically, developmentally related cell types exhibited a greater overlap of SG-biased genes compared to more distantly related cell types. For example, excitatory neurons and interneurons displayed a higher overlap of SG-biased genes compared to excitatory neurons and other non-neuronal cell types (Figs. S11, S12, S13, S14, S15).

We further explored this by using the original cell type annotations from individual datasets, which included additional sub cell types. For example, in the DISCO datasets, the neuronal population is further classified into many subtypes for both excitatory neurons and interneurons. Consistent with previous observations, the SG-biased genes were generally cell type-specific, even within the same major cell type. For instance, the various subtypes of excitatory neurons exhibited distinct differentially expressed genes (DEGs), with very few genes shared across the sub-populations (Figs. S11, S12, S13, S14, S15).

Cell type-specific SG-biased genes were mainly autosomal genes, and genes located on the X- and Y chromosomes tended to be shared across cell types (Figs. S11, S12, S13, S14, S15). One of the most shared SG-biased genes across all cell types and datasets was XIST, with female-specific expression (Figs. 2,  S15). The same was observed for Y-expressed SG-biased genes such as DDX3Y and USP9Y.

**Fig. 3:**
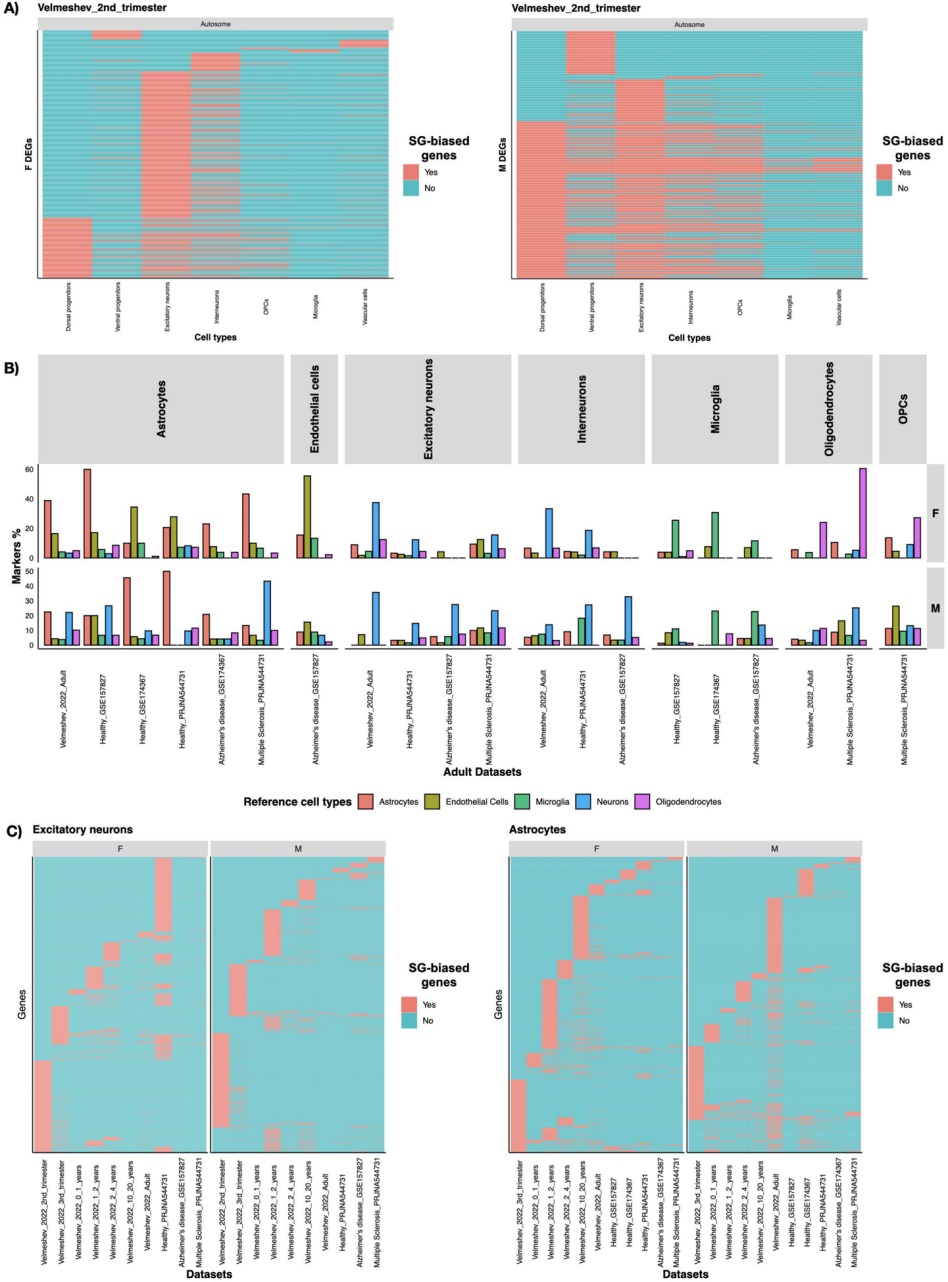
The SG-biased genes are cell type- and developmental stage-specific, and enriched for cell type specific markers. **A** Heatmaps for SG-biased genes across cell types in the second trimester datasets. **B** This bar plot shows the percentages of cell type markers found in the SG-biased genes in each cell type, by sex. For most of the cell types, the highest percentages corresponded to the expected cell type. For example, the excitatory neurons and interneurons have the highest percentages of the neuronal markers. **C** Heatmaps for SG-biased genes across datasets for excitatory neurons and astrocytes. Presence indicates whether the gene is found in each specific dataset-sex-cell type combination

We observed a cluster of shared male-biased genes across cell types in the second trimester dataset (Fig. 3A). The functional enrichment analysis of these shared genes showed that they were mainly involved in ribosomal and neurological diseases, including developmental pathologies (Fig. S17).

As SG-biased genes showed little overlap across cell types, we checked for the cell type-specific enrichment for known cell type markers, comparing the SG-biased genes with known genes from previously published data (McKenzie et al. 2018). Overall, for each reference cell type gene signature (astrocytes, endothelial cells, microglia, neurons and oligodendrocytes) greater overlap was observed in the corresponding cell types in our datasets. Exceptions were MS male patients, which showed higher percentage neuron markers for both astrocytes and oligodendrocytes, and female healthy patients which showed higher percentage of endothelial cells in astrocytes (Fig. 3B).

Since only samples from the cortex were included in this study, we investigated whether the SG-biased genes were specific to the cortex. A recent bulk RNA-seq study investigated sex differences in multiple tissues, including several brain regions (Oliva et al. 2020). We calculated the enrichment of the DEGs from this study in SG-biased genes, separately for each brain region. We found that the DEGs from the study were overall enriched in the female- but not in the male-biased genes (Fig. S18). The enrichment was found mostly in the data from early developmental stages, but not in the healthy adults (i.e. age-comparable samples to the original study) (Fig. S18). Moreover, SG-biased genes were found to be enriched in all brain region-specific DEGs from the study i.e. SG-biased genes were not cortex-specific (Fig. S19).

### SG-Biased Genes are Developmental Stage Specific

We also noted that most SG-biased genes were developmental stage specific (Figs. 3C,  S20), and a very few DEGs for each cell types were shared among at least 75% of the datasets (Supplementary file 2). Most shared genes across datasets in a given cell type were located on the X and Y chromosomes (Supplementary file 2), while the majority of the unique genes were located on the autosomes, similar to shared genes across cell types in a specific age group described in previous section. However, some autosomal genes were shared SG-biased genes across datasets, such as RGS1 in females (microglia), CADM2 and MT genes (interneurons) in females, and several genes in vascular cells (e.g. STMN1 in males) and oligodendrocytes, both in females and males.

In each cell type for each SG, gene overlap analysis showed that the groups closer in age had a higher overlap of genes compared to the other groups (Figs. S21, S22, S23, S24). Additionally, the shared genes were mainly autosomal, although the genes shared by most datasets belonged to X or Y chromosomes (Fig. S25). For some datasets, such a temporal relationship was not noted. For example, SG-biased genes in MS and AD datasets had a higher overlap with SG-biased genes at younger ages than adult in astrocytes and microglia (Figs. S23,  S24). Overall, significant overlap of SG-biased genes across related developmental stages across multiple cell types provided another proof of confidence in the SG-biased gene lists.

thus, the SG-biased genes not only were specifically expressed in each cell type across datasets, but also showed low overlap across age and disease groups within a cell type. A few highly shared genes across datasets were most located on the X and Y chromosomes.

### Functional Implications of SG-Biased Genes

**Fig. 4:**
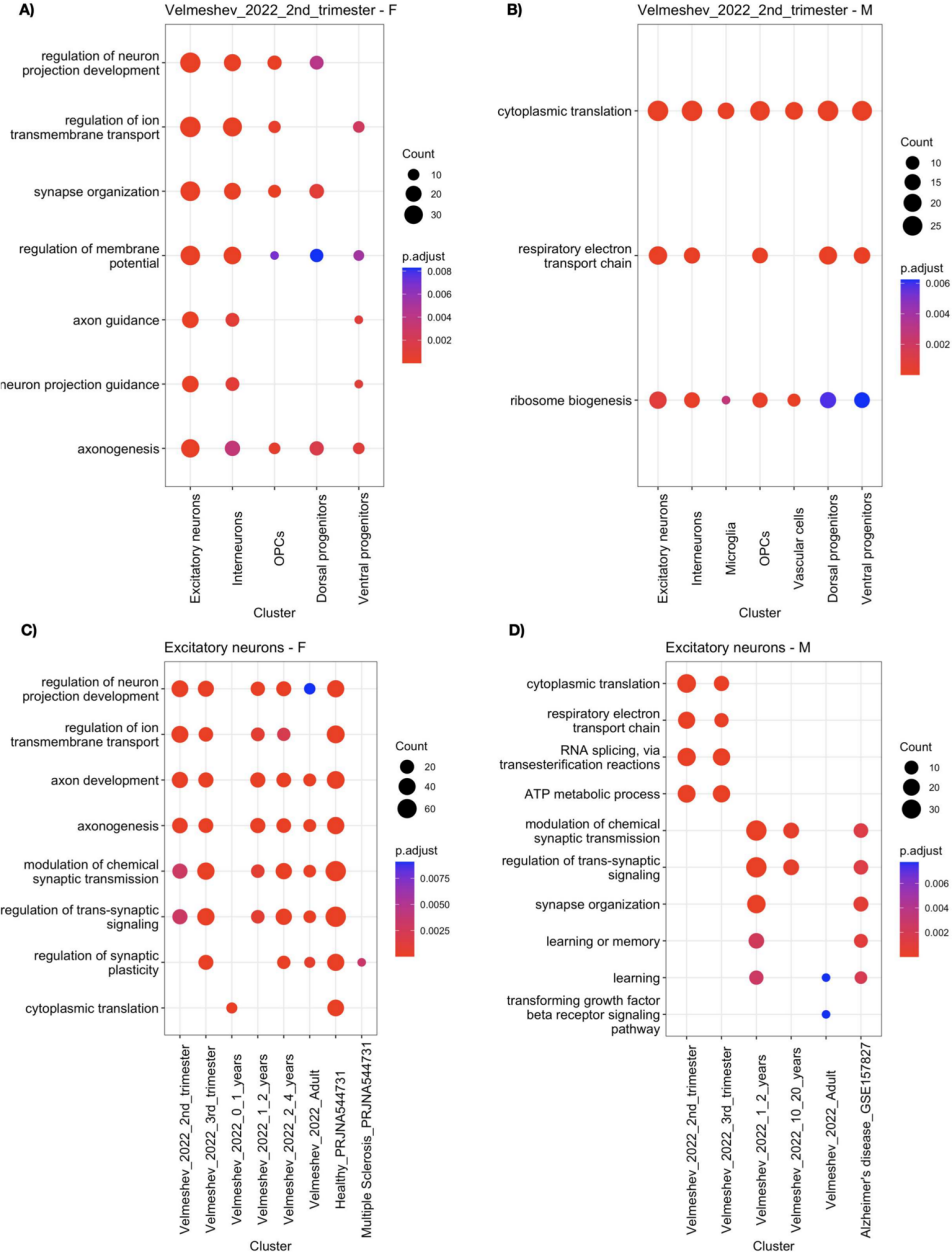
The SG-biased genes, despite being cell type- and developmental stage-specific, show overlap of enriched biological processes across both the cell types and the datasets. Both female- (**A**) and male-biased genes (**B**) showed overlap of enriched terms across different cell types, here as shown in the second trimester dataset. Similar findings were observed when instead of cell types, we compared multiple datasets in each cell type. Again, we observed an overlap in enriched biological processes both in female- (**C**) and male-biased genes (**D**) in excitatory neurons across datasets. The dot size indicate show many genes were found to belong to each GO BP term, and the color is the adjusted *p*-value (Benjamini–Hochberg correction)

To understand the likely functional implications of SG-biased genes, we calculated gene ontology (GO) enrichment for the biological processes (BPs) terms for all SG-biased gene lists. We firstly checked overlap of enriched BPs across cell types in each group. Though there was not much gene overlap of SG-biased genes across cell types and datasets (Figs. S21, S22, S23, S24, S25), many BPs were shared among different cell types, indicating similar pathways enriched at specific dataset (Figs. 4A, B,  S26, S27, S28, S29, Supplementary file 3). Across datasets, female-biased enrichment was primarily focused on brain-related BPs, such as neuron development, axonogenesis, and synapse-related terms. In contrast, BPs enrichment for males was mainly related to metabolism and cellular respiration. This difference was particularly pronounced in the earlier age datasets (second trimester to 2–4 years). However, in later age datasets, we observed brain-related functions enriched in males as well, especially in the MS and AD disease datasets (Figs. 4A, B,  S26, S27, S28, S29). We checked the overlap of GO BPs enrichments across the age groups for each cell type as well (Figs. 4C, D,  S30, S31, S32, Supplementary file 4). We again noted a high overlap of BP terms across datasets (Figs. 4C, D,  S30, S31, S32). Excitatory neurons from females consistently showed enrichment in synapse- and axon-related biological processes (BPs), which were not enriched in the excitatory neurons of males. Some BPs were enriched only at specific developmental stages. For example, the respiratory electron transport chain and other metabolic processes were enriched in excitatory neurons in males during the fetal stage but not later. Additionally, these processes were not enriched in the excitatory neurons of females in any dataset (Figs. 4C, D,  S30, S31, S32). We observed a consistent pattern across cell types: male-biased genes were primarily enriched for metabolic processes, while female-biased genes were mostly related to brain processes. For example, astrocytes in females showed enrichment in axon development and axonogenesis, whereas in males, the enriched BPs were predominantly metabolism-focused.

We further investigated enriched pathways using the Kyoto Encyclopedia of Genes and Genomes (KEGG) database. We noted a high overlap of enriched pathways across cell types in the fetal datasets, and cell types from older age groups did not display the same level of overlap (Figs. S33, S34, S35, S36, Supplementary file 5). Interestingly, most of the terms across all datasets were diseases and pathologies rather than physiological pathways, both neurological diseases and infections from a variety of pathogens, among which the most recurrent were prion, Parkinson’s and Coronavirus diseases (Figs. S37, S38, S39, Supplementary file 6).

We also investigated the enrichment of transcription factor (TF) binding sites using the TRANSFAC and JASPAR position weight matrices (PWMs) database in enrichR (Chen et al. 2013; Kuleshov et al. 2016). When TF motif enrichments for the cell types in each dataset were compared, TCFAP2A binding sites were enriched in female DEGs in 50% of the datasets. No highly shared TF binding sites were found in males (Figs. S40, S41, S42, Supplementary files 7 and 8). Similarly, the overlap of TF enrichments in each cell types across datasets showed that only two TFs were enriched at least in 50% of the cell types: TCFAP2A and POU1F1, both in females and no TFs were found in 50% of the cell types in males (Figs. S43, S44, Supplementary files 9 and 10). TCFAP2A target enrichment was found in excitatory neurons, interneurons, astrocytes, OPCs and dorsal progenitors, and mostly during the early development, from the second trimester of gestation to 2–4 years of age, and also in female MS patients (Figs. S43, S44). TCFAP2A enrichment was observed in males, specifically in excitatory neurons and interneurons at 10–20 years of age. In contrast, POU1F1 targets were almost exclusively enriched in females during early development (from the second trimester until 1–2 years of age). POU1F1 enrichment was found in females across various cell types, including excitatory neurons, interneurons, astrocytes, and dorsal and ventral progenitors. Additionally, POU1F1 was observed in male astrocytes and oligodendrocytes, respectively, in healthy adults (GSE174367) and in Velmeshev’s dataset for individuals aged 10–20 years.

### Pathological Implications of SG-Biased Genes

As SG-biased genes showed pathology-related term enrichment in pathway analysis, we explored further using the following disease databases: disease ontology (DO), DisGeNet and DisGeNET CURATED, and GWAS catalog 2019 (Supplementary files 11, 12, 13 and 14). After calculating the enrichment in each disease database, we selected the most common enriched terms in females and males in all datasets (Fig. S45A, Supplementary file 15). The most common enriched terms for females were drug use disorders and abuse, smoking, autistic behaviour and amyotrophic lateral sclerosis, among other brain-related pathologies. In males, the most common enriched disease terms were neoplasms, epilepsy, Alzheimer’s disease onset and other brain-related pathologies as well as for cancer-related terms. Additionally, we investigated the most common enriched terms for each cell type (Supplementary file 16). In females, astrocytes had enrichment for amyotrophic lateral sclerosis and androgen-insensitivity syndrome, while in males epilepsy-related terms were mostly enriched (Figs.  S45A,  S46). Excitatory neurons and interneurons were enriched in females for autistic behaviour and drug addiction-related disorders, while males displayed enriched terms for cancer-related terms, spermatogenic failure and epilepsy.

Autism is typically considered a male-biased disorder (Lai et al. 2017). Interestingly, we noted female-biased genes enriched for autism. We therefore used the SFARI autism genes database (https://gene.sfari.org/database/human-gene/) (Banerjee-Basu and Packer 2010) and calculated the number of SG-biased genes involved in autism (Figs. S47, S48). During early development (the second trimester and 1–2 years of age), nearly all cell types showed a higher autism-related gene overlap in females compared to males (Figs. S47, S48). In older age groups, either both female and male SGs had a similar number of autism-related genes, (e.g. in microglia), or males had more autism-related genes (e.g. in endothelial cells, and astrocytes). Additionally, most of these genes were located on the autosomes, indicating that the sex chromosomes were not the source of this bias (Fig. S48).

We also investigated the enrichment of disease-associated genes from the literature (Chlamydas et al. 2022) in our SG-biased genes. Notably, we found higher overlap of disease-associated genes with the female-biased genes than with the male-biased (Fig. S49). Two genes, KDM6A and PCDH11X had higher expression in females, and have been associated with a higher protection from the brain-related diseases (Chlamydas et al. 2022). Furthermore, KDM6A was expressed more ubiquitously in glial cells (astrocytes, oligodendrocytes), microglia and vascular cells (Fig. S49), and was expressed mainly in the younger datasets (third trimester until 20 years of age). PCDH11X showed a cell type-specificity, mainly neurons (Fig. S49) and was predominantly expressed in the early childhood.

Lastly, we investigated whether the SG-biased genes were enriched for drug targets, using the DSigDB database from enrichR. We noted that most of the enriched drug targets were present in both sexes (Fig. S45C, Supplementary files 17 and 18). The three most frequent enriched drugs in females were trichostatin, retinoic acid and valproic acid targets. Moreover, they were enriched in nearly all cell types in females. Males showed enrichment for these drugs as well, although to a lesser extent (Fig. S50, Supplementary file 19).

### Origins of the Sex and Gender Bias

We finally explored likely source of SG-biased gene expression. We have noted before that the majority of SG-biased genes were located on the autosomes (Figs. S11, S12, S13, S14, S15, S25). Sex-biased genes in some cell types (vascular cells, OPCs, microglia, interneurons and excitatory neurons) were enriched for the Y chromosome for the male-biased genes, while no enrichment was found for the X chromosome (Fig. S51). In previous microarray results, 91% of SG-biased genes in the dorsolateral prefrontal cortex were autosomal, higher than in other regions (Mayne et al. 2016), suggesting that indeed the sex bias in the cortex may be less dependent on the sex chromosomes than in other brain regions. We further explored whether the genes escaping X chromosome inactivation (XCI) (Mousavi et al. 2020) had a female-bias (Fig. S52). Firstly, only a few XCI genes were expressed SG differentially in brain. Furthermore, TMSB4X was more expressed in males than in females (Fig. 2). Interestingly, its Y-linked counterpart, TMSB4Y, was ubiquitously expressed at much lower levels in all datasets (Fig. S53).

Sex-divergent brain development is believed to be caused primarily by the testosterone exposure *in-utero*, where early testosterone exposure impacts social behaviours e.g. sexual orientation and gender identity, and brain structure and functions (Hines 2020; Bakker 2021). To determine if sex hormones might indirectly influence the expression of SG-biased DEGs, we examined the presence of androgen and estrogen response elements (AREs and EREs), which are downstream of the activation of androgen and estrogen nuclear receptors (Claessens et al. 2008; Rettberg et al. 2014), using previously published data (Bourdeau et al. 2004; Wilson et al. 2016). Circa 50% and 20% of tissue-specific genes had ARE and ERE sites respectively, including the brain (Fig. S54). While we found similar percentages for the ERE sites, we observed a consistent enrichment of ARE sites in almost all cell types and all datasets, with 75% and more of SG-biased genes in both females and males with full, half or full-half ARE sites (Figs. 6,  S55). However, we did not observe significant differences between females and males in ARE enrichment across datasets or cell types.

**Fig. 5:**
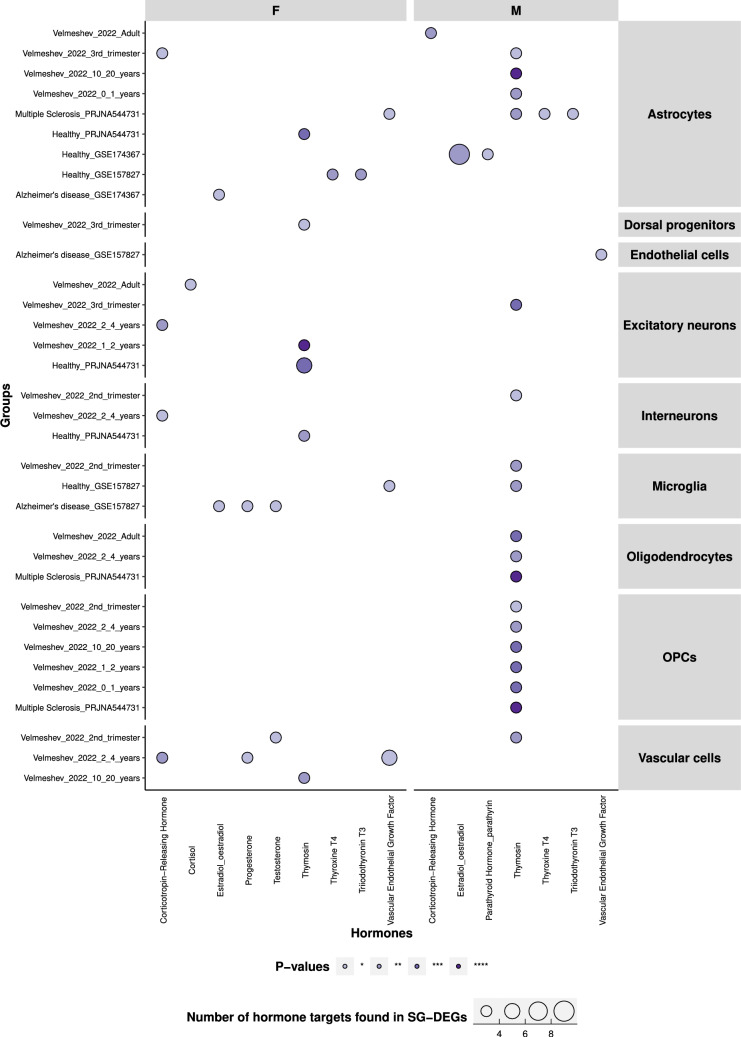
The SG-biased genes are enriched for several hormonal targets, among which is thymosin. This dotplot shows the number of hormone targets found and the *p*-values of hypergeometric enrichment for hormone targets from the literature in the SG-biased genes. Albeit most hormones are enriched in both sexes, there are some SG specific enrichments. For example, cortisol and progesterone are solely enriched in females. On the other hand, thymosin was strongly enriched in males, much more than in females, across multiple cell types and datasets. *NS* not significant; *: *p*<0.05; **: *p*<0.01; ***: *p*<0.001; ****: *p*<0.0001

We further calculated the enrichment of the targets of 53 hormones, including testosterone, in the SG-biased genes, using a reference database (Jadhav et al. 2022). The hormones enriched in more than one dataset were likely to be true positives (Fig. 5, Supplementary file 20). Overall, many hormone targets were found in several cell types, but predominantly in females, with one main exception (thymosin). Interestingly, testosterone targets were enriched in one of the Alzheimer’s disease (AD) groups in microglia in females and in vascular cells during the second trimester, also in females. No enrichment was observed for testosterone targets in males. Estradiol and progesterone both showed enrichment in microglia in one of the AD datasets. Corticotropin-releasing hormone also showed enrichment mostly in females (Fig. 5).

Thymosin was the most enriched hormone, across multiple cell types but mainly in males (Fig. 5). Thymosin targets were notably enriched in male-biased OPCs, oligodendrocytes, and astrocytes, particularly in younger males and those with MS. Previous studies have investigated the role of specifically thymosin $\beta$10 and $\beta$4 in the developing brain and in neuroembryogenesis (Dalakas and Trapp 1986; Hall 1991; Condon and Hall 1992). These two products of thymosin are encoded respectively by TMSB10 and TMSB4X, which were among the top 10 most unique genes in males (Fig. 2) and the X-escaping genes also expressed in males (TMSB4X, Figs. 2,  S52).

**Fig. 6:**
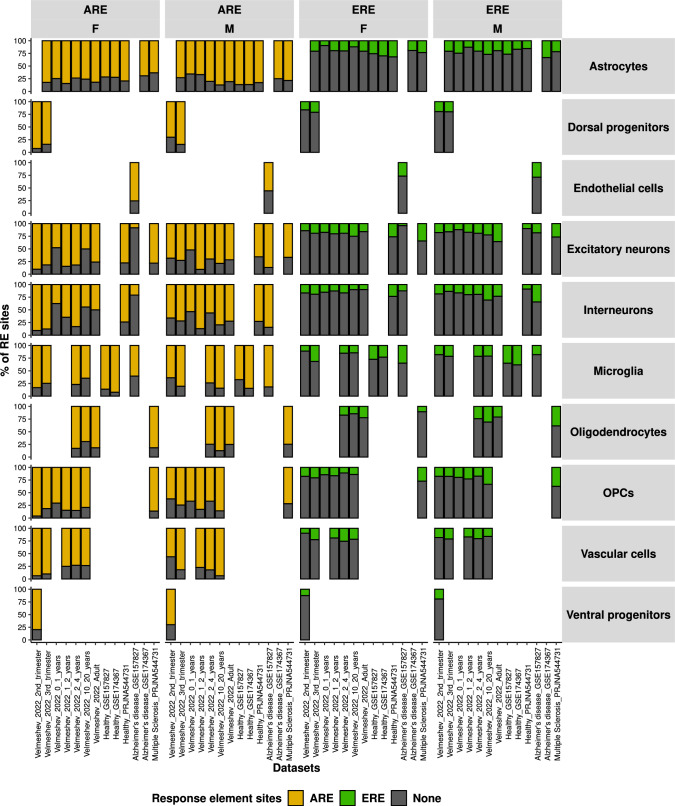
Androgen response element binding sites, but not estrogen, are consistently enriched in SG-biased genes across sexes, cell types and datasets. Androgen response element (ARE) and estrogen response element (ERE) sites presence in the SG-biased genes, by sex, cell type and dataset. The majority of the SG-biased genes had ARE binding sites, in both sexes. ERE sites instead were present in less than one fourth of SG-biased genes overall. “None” indicates SG-biased DEGs which did not have neither ARE nor ERE binding sites

In summary, sex difference in the human brain cortex were largely associated with hormones, specifically thymosin and testosterone, than sex chromosomes.

### Rshiny Resource to Explore for the Community

We developed a web resource to provide the entire analysis for the scientific community and demonstrate the robustness of the analysis by allowing different thresholds for analysis. SGHumanBrainApp (https://joshiapps.cbu.uib.no/GenderRank_app/) is a web application built with R shiny (Chang et al. 2023), an interactive tool for the user to explore the datasets included in this study, together with SG analysis from bulk RNA-seq data in brain from a previous study (Wapeesittipan and Joshi 2023). The application consists of six tabs. The first tab is a general explanatory section that provides an overview of the application and the included studies. The second tab details the datasets used in this study, including the number of samples, ages, number of cells, and other relevant information. The third tab is a SG-biased genes analysis tab that allows users to explore the main results of our analysis on SG-biased genes. Users can select thresholds for adjusted *p*-value and fold change (FC). Once selected, users can view an overview of SG-biased genes (number of SG-biased genes, chromosome fractions, cellular location), heatmaps of genes of interest (most frequent sex-specific DEGs, mitochondrial and X-escaping genes), expression of cell and disease markers, hormone target enrichment, percentage of genes with ARE and ERE sites, and functional enrichment (GO—biological process, cellular components, molecular function- and KEGG pathways). All plots can be individually downloaded. Additionally, users can download all plots and the unfiltered or filtered SG-biased genes together in one ZIP folder. The fourth and fifth tabs contain the SG analysis from the bulk RNA-seq study (Wapeesittipan and Joshi 2023), where the users can either explore SG DEGs in individual brain regions (fourth tab) or across all brain regions included in the bulk RNA-seq study (fifth tab), also using different thresholds. The sixth tab contains information about the source code to generate the application. The source code of the SGHumanBrainApp application is available at https://github.com/aurazelco/SGHumanBrainApp. The scripts used to generate the output files for the single-cell DEGs tab can also be found in the repository.

## Discussion

SG affects many regions of brain. As the cortex is one of the brain regions with most of anatomical SG differences (Arain et al. 2013; Lombardo et al. 2012b; Neufang et al. 2009; Fuhrmann et al. 2022), we collected and analysed publicly available single-nucleus transcriptomic data in the cortical region to characterize SG differences throughout healthy lifespan from *in-utero* to aging, including two diseases, AD and MS, the latter known to be highly SG-biased in incidence and progression (Leffler et al. 2022; Ysrraelit and Correale 2019). Gene expression is highly specific to a cell or tissue type and within a cell or tissue type, age affects gene expression more than sex (Kang et al. 2011). Therefore, it is essential to distinguish sex and gender (SG) differences by stratifying based on age and cell type complexity, particularly in a complex and dynamic organ like the brain. The main goal of this research was to identify small but potentially significant SG differences. Thus, it was crucial to first categorize the samples by age and cell type, as SG differences are smaller in magnitude compared to age and spatial variations and are likely to be overshadowed. Indeed, SG-biased genes were present in most cell populations: neurons (excitatory, interneurons), glial cells (astrocytes, oligodendrocytes, OPCs), astrocytes, and vascular cells in most datasets, and dorsal and ventral progenitors only in the fetal datasets for physiological reasons. Endothelial and T cells either had too few cells or too few differentially expressed genes to be included for further analysis. The SG-biased genes were cell type-specific, developmental stage-specific, and had a low overlap across datasets. A small overlap in SG-biased genes across cell types and developmental stages might partly be because SG differences are small as cell types populations primarily separate according to age (Velmeshev et al. 2023) or disease (Morabito et al. 2021; Schirmer et al. 2019). Despite SG-biased genes being cell type- and developmental stage-specific, the enriched terms for gene ontology, KEGG pathways, and diseases showed significant overlap. Overall, most female-biased genes were enriched for brain-related biological processes, while male-biased genes were more enriched for metabolic functions and pathways. This aligns with previous findings that males generally have a higher metabolic rate (Biskup et al. 2019), while female-biased genes were more enriched for neuronal processes (Wapeesittipan and Joshi 2023; Shi et al. 2016). In this research, where data from original studies were reanalyzed, solely one study (Velmeshev et al. 2023), included a SG-focused analysis, albeit as a minor component of their comprehensive investigation (Table 3). The findings from this analysis, unsurprisingly matched with our findings. e.g. they found that female-biased genes in excitatory neurons and astrocytes were enriched for GO terms of neuronal development, while male-biased genes were linked to protein translation, similarly to our GO enrichment analysis. Additionally, they also found no X chromosome enrichment in the female DEGs.

**Table 3:** Summary of sex/gender-focused analysis in the source datasets

Study ID	Sex/gender analysis
GSE157827	Briefly mentions a similar change in DEGs but to a different degree in the two sexes
GSE174367	Only analyzed as a confounding factor
PRJNA544731	Only analyzed as a confounding factor
Velmeshev	Sex-specific analyses performed concomitantly with trajectory analyses, GO and disease enrichment (ASD, SFARI)

One of the interesting findings was a female bias for mitochondrial genes, across cell types and developmental stages, particularly in astrocytes, excitatory neurons, and interneurons. We investigated in depth whether mitochondrial enrichment could be the result of technical bias or resulting into lower quality of samples. Cell removal based on the percentage of mitochondrial genes, is a common step in single-cell and single-nuclei data analysis. Only one of the original studies completely removed mitochondrial genes (Schirmer et al. 2019), while the others removed cells with mitochondrial gene percentages above 10–20% (Lau et al. 2020; Morabito et al. 2021; Velmeshev et al. 2023) or combined this parameters with others in a quality control score (Li et al. 2022). In our analysis, we did not remove mitochondrial genes but used a stricter threshold (5%) for cell removal. This is a conservative approach despite the higher presence of mitochondrial transcripts in metabolically active tissues like the brain (Ding et al. 2020). Furthermore, mitochondrial read fractions in single nucleus and single cell RNA-seq arelower or comparable to bulk RNA-seq (Ding et al. 2020), suggesting that mitochondrial gene expression is likely not an artifact. The presence of mitochondrial genes indicates cytoplasmic contamination, which ideally should not occur in single-nuclei sequencing studies. However, these signals are likely due to compartmental contamination from the cytoplasm of the same cells from which the nuclei are extracted, and could be biologically relevant. Therefore, they should not be disregarded as irrelevant. We also investigated whether the female-biased mitochondrial enrichment is simply due to compartmental contamination. Given that ribosomal genes (S56, S57, S58) do not exhibit a similar enrichment pattern, it further supports the hypothesis that the female bias of mitochondrial genes might likely be of biological origin. Furthermore, we would not see consistent patterns in female-biased genes. Our additional down-sampling analysis also shows a consistent mitochondrial gene presence in SG-biased down-sampled sets. We hypothesize that such findings highlight potential true biological differences, which may have gone unnoticed until now, due to a common practice of ignoring mitochondrial genes in the down-stream analysis. Mitochondrial gene up-regulation was especially present in neuronal population, as it has shown that mitochondrial metabolism is strictly connected to neuronal development (Iwata et al. 2023), and neurons are among the most energy-demanding cell types in the brain (Frackowiak et al. 2002). Given that neurons are mitochondria-rich and astrocytes are strategically positioned near synapses, it is suggested that mitochondria play a crucial role in neuronal glucose metabolism (Frackowiak et al. 2002) and influence the timing of neuronal maturation (Iwata et al. 2023). A recent study showed significant hypomethylation on mitochondrial DNA on multiple *loci* in females compared to males, likely leading to up-regulation of mitochondrial genes (Devall et al. 2022). Additionally, estrogens have shown to regulate mitochondrial functions, providing support for a female-biased mitochondrial expression (Klinge 2020).

Disease enrichment analysis showed female-biased genes enriched for autism, amyotrophic lateral sclerosis 1 and drug abuse, while male-biased genes enriched for neoplasms. Interestingly, both sexes showed enrichment for epilepsy, albeit slightly more in male-biased genes. Other literature is in agreement with a female bias with ovarian hormone effects on e.g. drug abuse (Moran-Santa Maria et al. 2014; McHugh et al. 2018; Marinelli et al. 2023), smoking (Gritz et al. 1996; Pauly 2008; Schiller et al. 2012) and amyotrophic lateral sclerosis (Trojsi et al. 2020). To note, the neurobiological causes of SG differences might not correlate only with estrogen but other ovarian hormones such as e.g. testosterone in cognitive decline (Gurvich et al. 2018). Previous results indicate that male DEGs showed enrichment for autism-related genes (Shi et al. 2016), however more recent results showed female-biased enrichment for autism-related genes (Wapeesittipan and Joshi 2023; O’Brien et al. 2019). The “Female protective effect” has been shown in autism, where females require more mutations to develop autism (Wigdor et al. 2022). Similar to us, this was proposed as a hypothesis for the female bias in autism-related genes by a previous publication (Velmeshev et al. 2023), whose data is part of this study. We noted highly significant enrichment for autism in female-biased genes, particularly in the earlier ages (second trimester until early childhood). This is in agreement with a recent work (Li et al. 2018), where weighted gene co-expression network analysis (WGCNA) was performed on different brain regions and at different ages. One module of the WGCNA in the excitatory neuronal population, was enriched for autism among other neurological disorders, and also more enriched in the fetal datasets compared to the older ones, albeit no difference between the sexes was observed. Another study also found autism risk gene enrichment in the human fetal brain during the second trimester of gestation, with similar enrichment between males and females (O’Brien et al. 2019).

Sex is thought to affect the brain via gene expression from sex chromosomes and the role of by sex hormones (Arain et al. 2013; Bakker 2021). The SG-biased genes were mainly present on the autosomes, with surprisingly little Y and no X chromosome enrichment. 91% of the SG-biased genes in the dorsolateral prefrontal cortex were indeed autosomal, more than in other brain regions included in the study (Mayne et al. 2016). These results suggest that SG differences, at least in the cortex, might originate mainly from the autosomal genes. Importantly we noted that the most common SG-biased genes across cell types and ages were present on sex chromosomes. This is in agreement with the previous findings that showed X-linked genes most frequently sex differentially expressed across different brain regions (Oliva et al. 2020; Wapeesittipan and Joshi 2023). The current paradigm is that brains are masculinised by the expression of sex-biased genes from conception and pubertal surges in testosterone in males (Forest et al. 1973; Kuiri-Hanninen et al. 2011). Similarly female-bias is manifested by the sex-biased genes and estrogen surge at puberty driving the SG differences (Beking et al. 2018; Bramble et al. 2017; Lai et al. 2017; Lombardo et al. 2012a; Christine Knickmeyer and Baron-Cohen 2006). For instance, it has been suggested that physiological testosterone decline in adult males and its absence in females throughout brain development enhance the risk for AD (Pike 2017). We examined both sex and non-sex hormones to identify potential sources of SG bias. Our enrichment analysis of SG-biased genes for hormone targets revealed a few key hormones. Notably, microglia from female Alzheimer’s disease patients showed enrichment for testosterone, progesterone, and estradiol targets. Additionally, corticotropin-releasing hormone was enriched across multiple cell types in females. Interestingly, sex hormone targets were not highly enriched in the SG-biased genes. A possible reason for this might be that the hormone-target database was rather conservative. Additionally, the expression of hormones varies greatly across brain regions (Quintana et al. 2019). Both estrogen and androgens (testosterone), can influence expression via nuclear receptors and specific response elements, called androgen and estrogen response elements (ARE, ERE) (Claessens et al. 2008; Rettberg et al. 2014). AREs and EREs have documented sex-biased expression in the brain, with females expressing more EREs and males more AREs (Gegenhuber and Tollkuhn 2020), and it has been hypothesized before that estrogen through EREs can influence SG gene expression differences in mice (Gegenhuber et al. 2022). Research on androgen response elements (AREs) in the brain remains limited, with current findings predominantly from non-human species (Gopalakrishnan et al. 2022; Zhang et al. 2012; Lai et al. 2023; Jeong et al. 2006; Xiao et al. 2009). One study demonstrated the role of testosterone via AR in hyper-androgenism during pregnancy and its effects on fetal development (Gopalakrishnan et al. 2022), underscoring the importance of androgen response elements in females. Another study revealed a potential link between age-related decreases in estrogen or androgen and the accumulation of amyloid beta via androgen response elements (Xiao et al. 2009), highlighting the role of androgen response elements in the brain pathologies. Despite these handful of studies, the role of AREs in the human brain remains significantly understudied. We found that almost ubiquitously, the more than 75% of SG-biased genes had ARE binding sites, while the percentage of ERE sites was less than 25%, with no difference between female- and male-biased genes for both AREs and EREs. We also discovered that ARE enrichment was present in SG-biased genes across most tissues using bulk RNA-seq data, although at a lower level compared to our study. Our results largely agree with previous findings, where similar percentages were found across multiple brain regions (Wapeesittipan and Joshi 2023). Testosterone’s bidirectional modulation of glucose and energy balance is a highly sexually dimorphic aspect of metabolic regulation. During the second trimester of pregnancy, increased testicular testosterone leads to brain masculinization, coinciding with hypothalamus development, which controls reproduction and metabolism. In the brain, testosterone can be converted by aromatase into estradiol, acting on estrogen response elements (EREs), or by 5-reductase into dihydrotestosterone (DHT), acting on androgen response elements (AREs). Estradiol is not required for the organizational effects of testosterone in humans, and energy balance is influenced by testosterone. However, it remains unclear whether this influence is mediated by estrogen receptors (ER) or androgen receptors (AR) (Morford and Mauvais-Jarvis 2016). Our analysis suggests that it might be mediated by AR at least in human cortex.

However, the most striking SG bias was observed for thymosin. Thymosin targets were enriched in male SG-biased genes in 9 of 10 cell types, across multiple ages and both in healthy individuals and MS patients. Thymosin is a hormone produced by the thymus, for the maturation process of T cells. However, its products has been found in other tissues, and have been studied in relation to brain pathologies (Kim et al. 2021; Bruschi et al. 2021; Xiong et al. 2022; Zhang et al. 2020). Indeed, TMSB10 and TMSB4X, thymosin products, were among the most frequent male-biased genes in our analysis. Interestingly, TMSB4X is an X-escaping genes (Wainer Katsir and Linial 2019). However, contrary to the other X-escaping genes, which were mostly absent from the SG-biased genes (XIST excluded), it was found more among the male-biased genes. TMSB4X has been studied as a candidate biomarker for childhood brain tumours (Bruschi et al. 2021), and it has been shown to be up-regulated in bipolar disorder and even more in major depressive disorder (Kim et al. 2021). On the other hand, researchers have shown that thymosin $\beta$4, encoded by TMSB4X, has neuroprotective properties, and it has been hypothesized it could a therapeutical target for neurological diseases (Zhang et al. 2020; Morris et al. 2010). However, the SG-specific role of TMSB4X in both physiological and pathological conditions is still not fully characterised. It has been shown recently that TMSB4X acts a a global exerkine and with increased levels in plasma after exercise, independent of exercise mode or resistance to insulin (Gonzalez-Franquesa et al. 2021). This postulates that higher TMSB4X level in cortex of males compared to females might reflect higher metabolic demand of the male brain.

### Limitations of the Study

The lack of sex-stratified studies, partly because of long tradition of male-biased research sampling in pre-clinical studies and clinical trials (Hägg and Jylhava 2021), made difficult to compare some of the novel results, such as ARE sites enrichment in our SG-biased DEGs. We validated the consistency of our finding by applying various thresholds (explorable in the web application). The key discoveries are largely preserved across different developmental stages and cell types. An integrated analysis of SG genes, from data from various studies, similar to our previous study (Aldinger et al. 2021), would have been ideal. Unfortunately, single-cell datasets are not as abundant as bulk datasets, and multiple independent datasets are not available for most developmental categories. This limitation prevented us from using methods like random down-sampling of cell types, as it would have resulted in the identification of very few SG-biased genes in many cell populations. This highlights the need for validation through independent experiments. Another challenge in our analysis was the variability in brain region sampling. Although all datasets and samples were from the cortex, the cortex itself has sub-regions. The lack of detailed sample annotation in the original studies’ metadata was a significant bottleneck. Furthermore, other brain regions, such as the hippocampus, which plays a central role in behavior and exhibits known SG differences in anatomy (Kight and McCarthy 2020; Neufang et al. 2009), or other specific brain regions have shown to regulate signs of aggression and mating dependent on sex or estrous state (Knoedler et al. 2022; Karigo et al. 2021; Bayless et al. 2019; Osakada et al. 2018; Wei et al. 2018; Hashikawa et al. 2017; Yao et al. 2017; Ishii et al. 2017; Unger et al. 2015; Hong et al. 2014; Lee et al. 2014; Yang et al. 2013; Lin et al. 2011; Musatov et al. 2006), were not included.

**Fig. 7:**
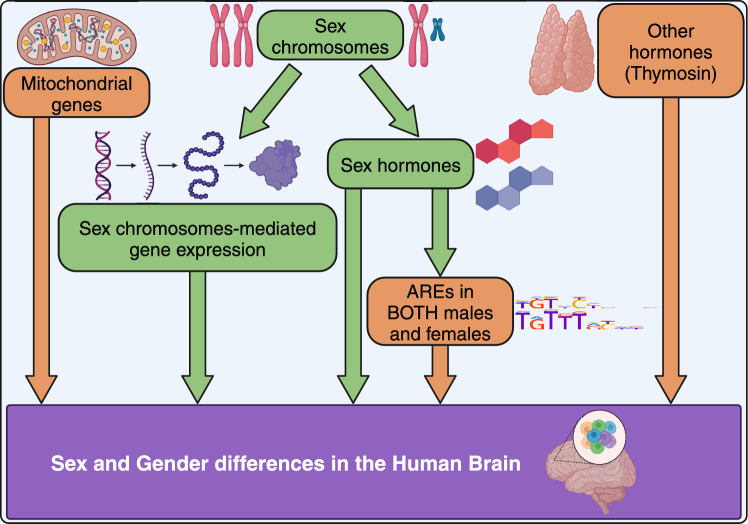
Summary of the sex and gender differences found in the human cortex transcriptome. After stratification by cell type and developmental stage, we identified SG-biased genes in human cortex transcriptome. Comprehensive analysis of SG-biased genes resulted in main findings; the female-biased mitochondrial gene up-regulation, the presence of ARE binding sites at the SG-biased DEGs for both sexes, and the enrichment of hormone targets other than sex hormones, such as thymosin. Our findings (highlighted in orange) further extend the traditional model of sex and gender differences in the human brain (highlighted in green). The ARE motif (split in two lines) was obtained via the HOmo sapiens COmprehensive MOdel COllection (version 12) *ARE* androgen response elements

## Conclusions

In conclusion, we have thoroughly characterized sex and gender (SG) differences in the human brain transcriptome across the entire lifespan, including data from Alzheimer’s disease (AD) and multiple sclerosis (MS) patients. The current study, designed specifically to discern SG difference in the human brain cortex, is significantly more exhaustive than other recent similar publications (e.g. Lopez-Cerdan et al. 2024; Neale et al. 2024; Xia et al. 2024; Pottmeier et al. 2024), and additionally provides a resource for scientists to explore sex and gender transcriptomic differences in the human cortex, at a single-cell level, spanning the whole life-span. We demonstrated that SG-biased gene expression is present not only among different brain cell types but also within the same cell type across all ages. We found a female-biased up-regulation of mitochondrial genes, while male-biased genes were enriched for thymosin hormone targets. Additionally, we observed the enrichment of androgen response elements in SG-biased genes in both males and females. In summary, the three novel and key findings described above in this study (highlighted in orange) further extend the traditional model of sex and gender differences in the human brain (highlighted in green) (Fig. 7). The main results included in this study are publicly available and can be interrogated further at https://joshiapps.cbu.uib.no/GenderRank_app/.

## Supplementary Information

Below is the link to the electronic supplementary material.

Supplementary file 1 (PDF 45016 KB)

Supplementary file 2 (CSV 1 KB)

Supplementary file 3 (CSV 7 KB)

Supplementary file 4 (XLSX 457 KB)

Supplementary file 5 (XLSX 451 KB)

Supplementary file 6 (XLSX 104 KB)

Supplementary file 7 (XLSX 89 KB)

Supplementary file 8 (XLSX 119 KB)

Supplementary file 9 (CSV 1 KB)

Supplementary file 10 (XLSX 96 KB)

Supplementary file 11 (CSV 1 KB)

Supplementary file 12 (XLSX 69 KB)

Supplementary file 13 (XLSX 604 KB)

Supplementary file 14 (XLSX 102 KB)

Supplementary file 15 (XLSX 95 KB)

Supplementary file 16 (CSV 2 KB)

Supplementary file 17 (CSV 7 KB)

Supplementary file 18 (XLSX 124 KB)

Supplementary file 19 (CSV 1 KB)

Supplementary file 20 (CSV 6 KB)

Supplementary file 21 (CSV 5 KB)

## Data Availability

The datasets generated and/or analysed during the current study are available in the DISCO and UCSC Cell Browser repositoris, respectively at https://www.immunesinglecell.org/download and at https://cells.ucsc.edu. The specific projects IDs and authors are reported in the methods. The scripts used for the analysis and for the web application can be found at https://github.com/aurazelco/SGDifferencesHumanBrain and https://github.com/aurazelco/SGHumanBrainApp.
