# Supplementary material for: Single-Cell Analysis of Sex and Gender Differences in the Human Brain During Development and Disease
Source: Cell Mol Neurobiol. 2025 Feb 27;45:20. doi: 10.1007/s10571-025-01536-2 (PMC11868228; doi:10.1007/s10571-025-01536-2)
Supplement: Supplementary file 1 — (PDF 45016 KB) [file 10571_2025_1536_MOESM1_ESM.pdf]

Supplementary figures

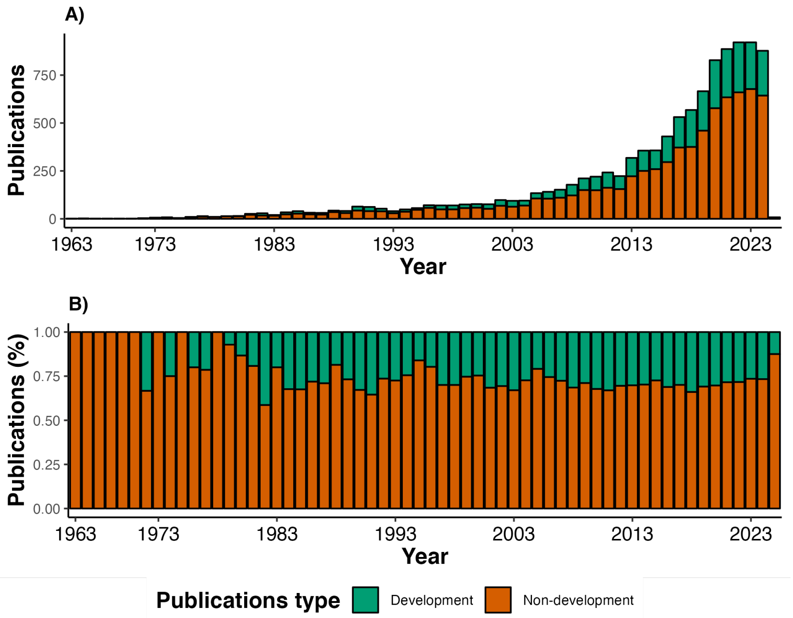

Fig. S1: **PubMed results including keywords for sex differences and brain development.** Results over the years for PubMed search: ”sex differences” AND ”brain development” as of late 2024. While the number of publications investigating brain development and sex differences has increased, it is still the minority of publications.

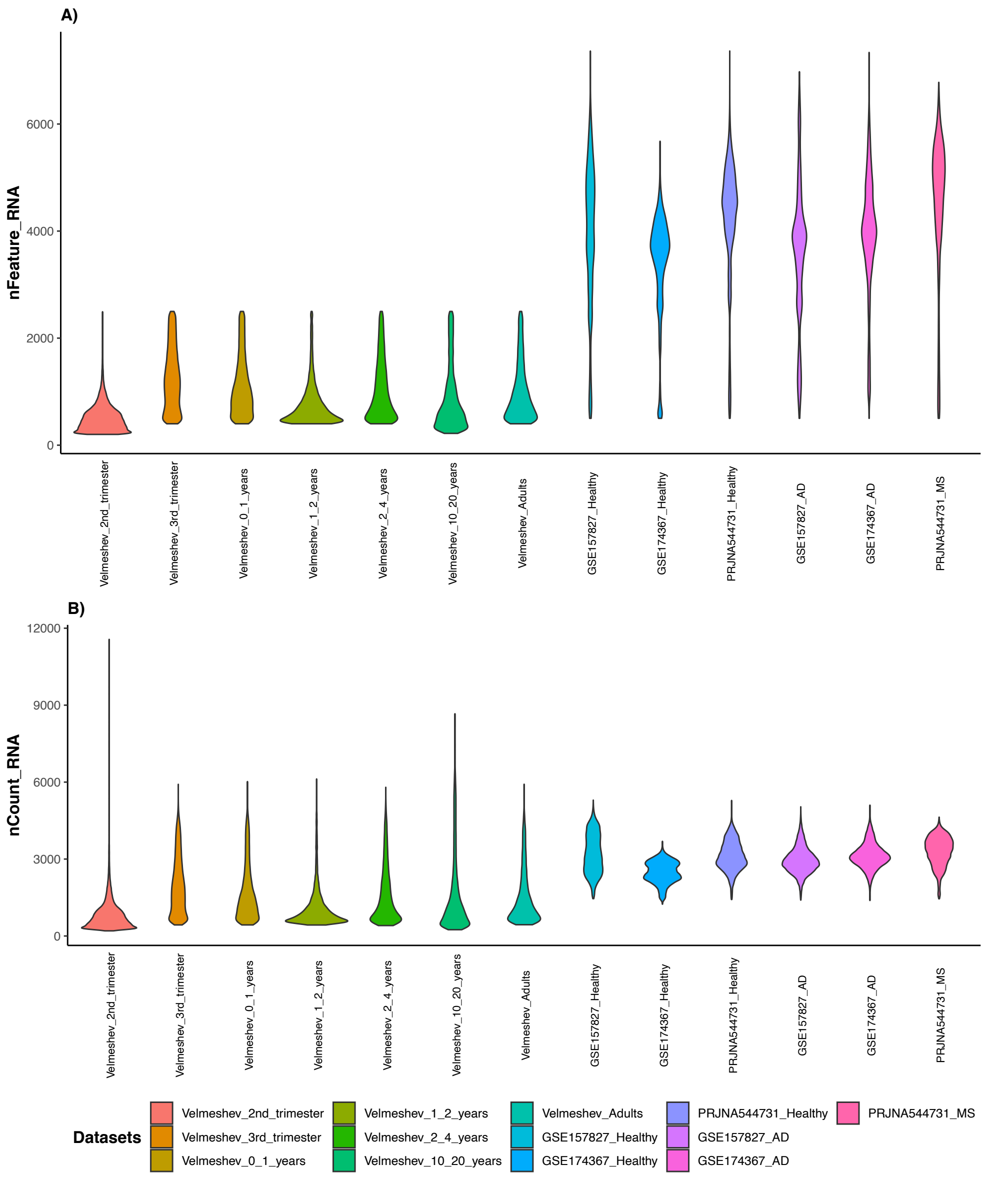

Fig. S2: **Summary figures of gene coverage parameters in the data sources.** A) Genes detected per cells (nFeature_RNA) and B) nCount_RNA distributions in the data sources.

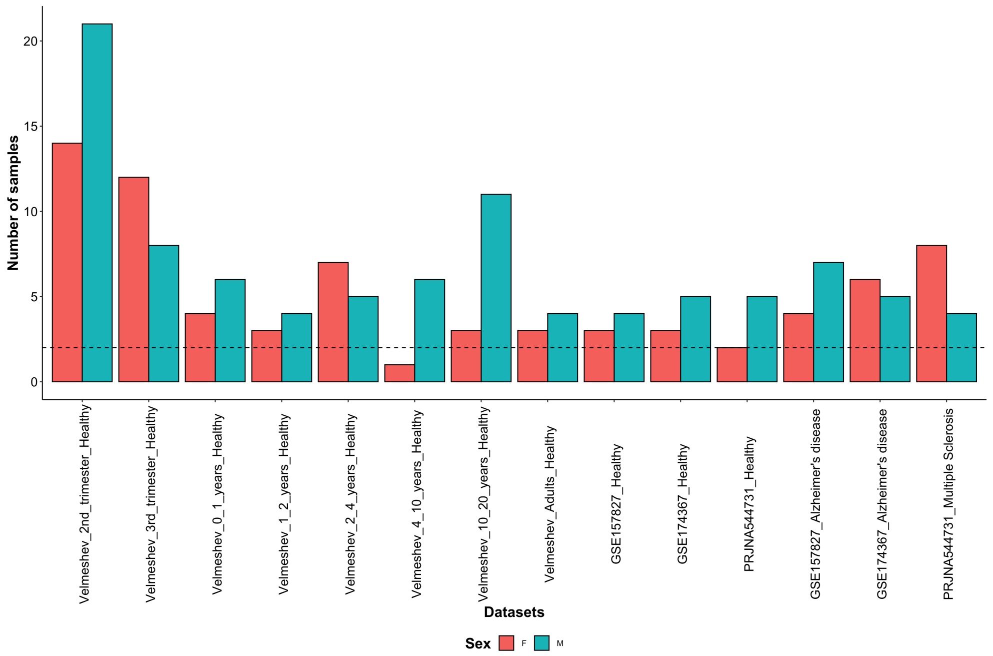

Fig. S3: **Number of samples in each dataset analyzed, separated by sex.** All datasets but one - Velmeshev 4-10 years - had at least n=2 (dashed line) of both female and male samples.

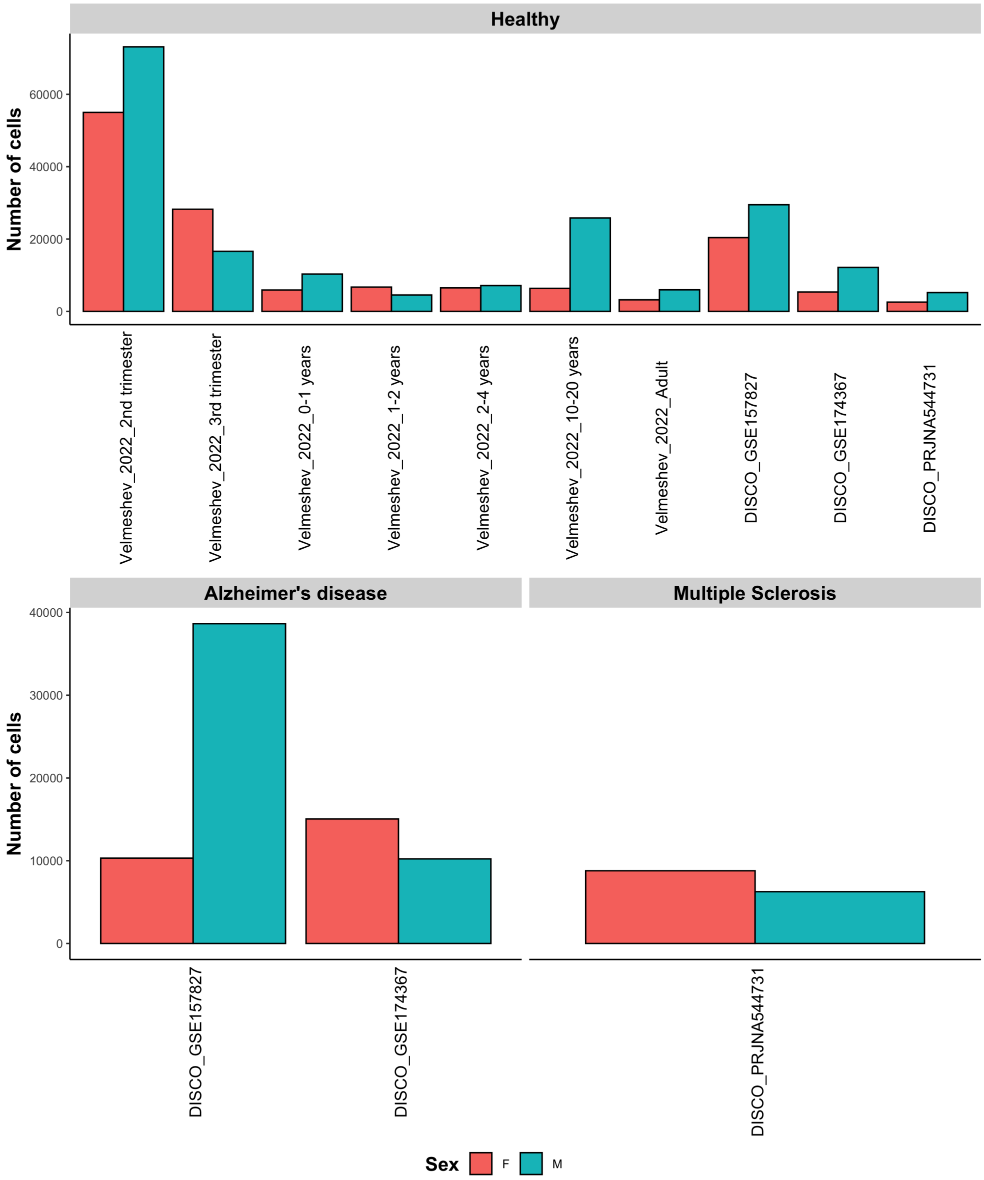

Fig. S4: **Number of cells in each dataset analyzed, separated by sex.**

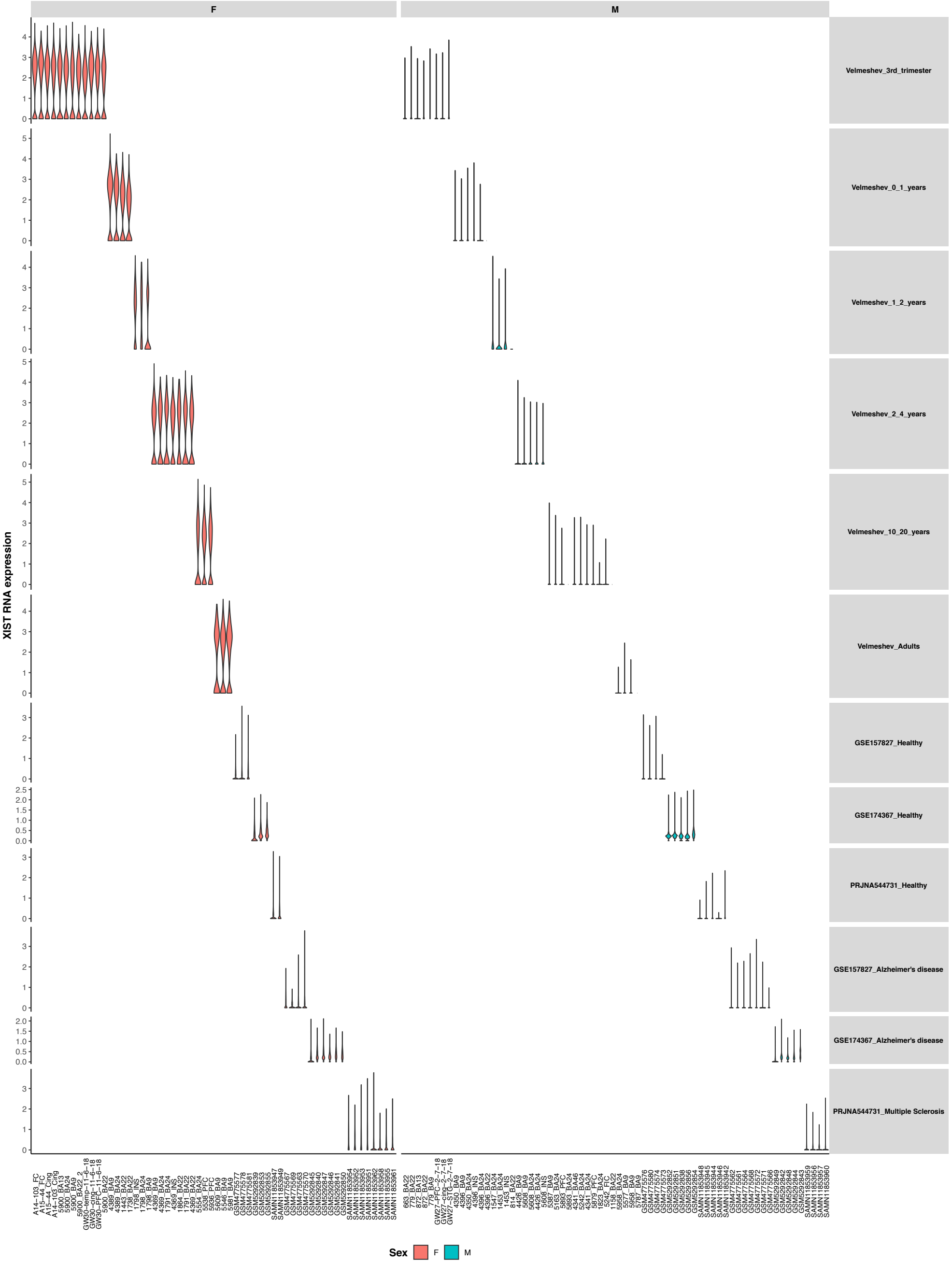

Fig. S5: XIST expression in the individual samples analyzed, organized by sex and dataset. Overall, all females samples showed a higher expression of XIST compared to males, as expected.

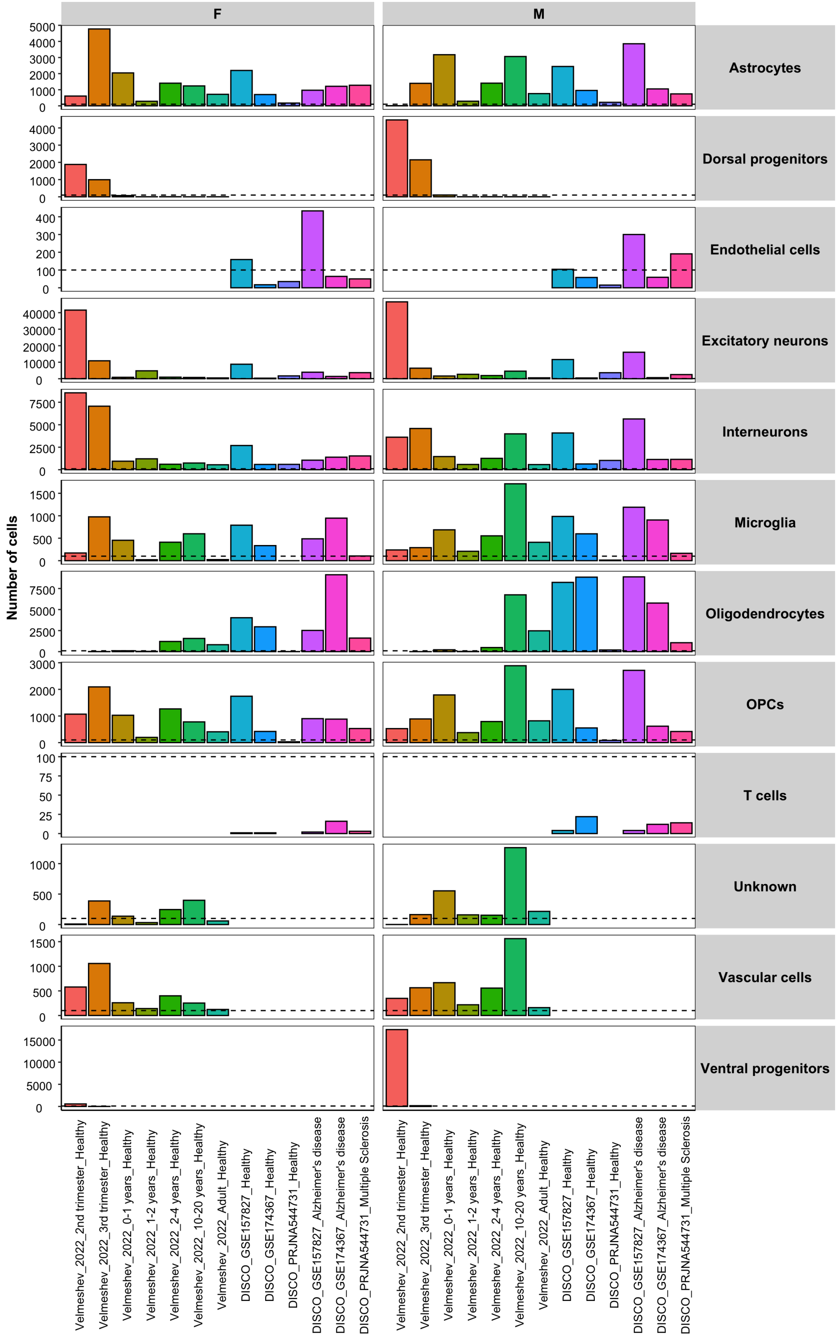

F
M
Astrocytes
Dorsal progenitors
Endothelial cells
Excitatory neurons
Interneurons
Microglia
Oligodendrocytes
OPCs
T cells
Unknown
Vascular cells
Ventral progenitors
Number of cells
Velmeshev_2022_2nd trimester_Healthy
Velmeshev_2022_3rd trimester_Healthy
Velmeshev_2022_0-1 years_Healthy
Velmeshev_2022_1-2 years_Healthy
Velmeshev_2022_2-4 years_Healthy
Velmeshev_2022_10-20 years_Healthy
Velmeshev_2022_Adult_Healthy
DISCO_GSE157827_Healthy
DISCO_GSE174367_Healthy
DISCO_PRJNA544731_Healthy
DISCO_GSE157827_Alzheimer's disease
DISCO_GSE174367_Alzheimer's disease
DISCO_PRJNA544731_Multiple Sclerosis

Fig. S6: **Number of cells in each dataset and cell type analyzed, separated by sex.** Most cell types had 100 or more cells (dashed line: 100 cells) in both female and male samples. Some cell types, e.g. T cells, were not high enough in numbers to be included in the downstream analysis.

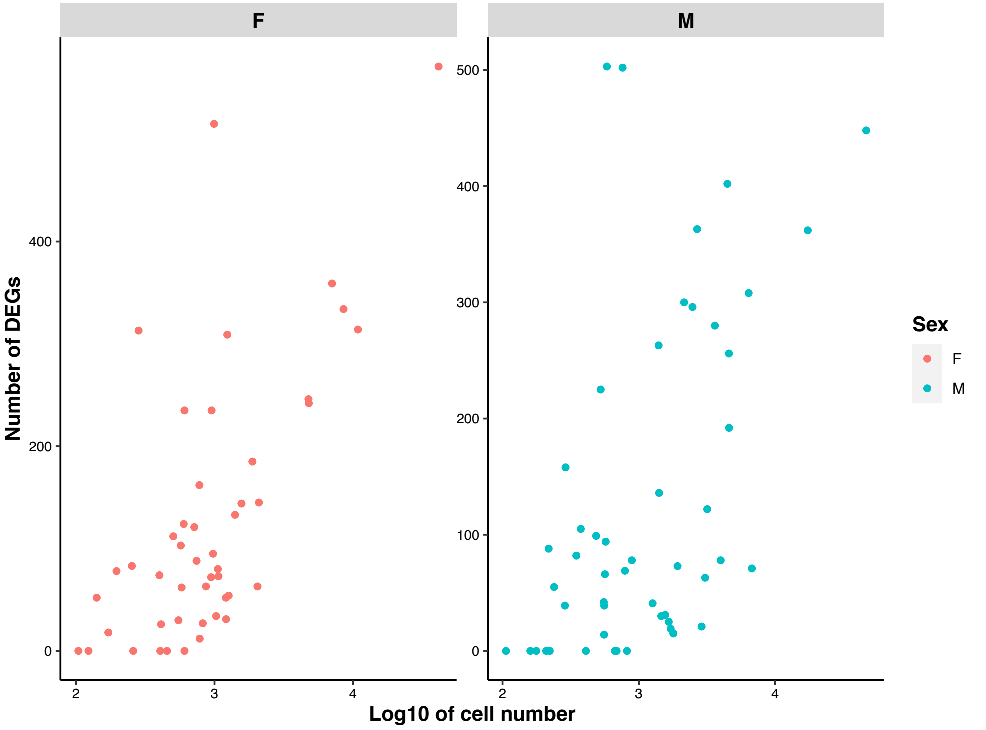

Fig. S7: **Number of cells in log10 scale** ***versus*** **number of DEGs, split by sex.** As seen from the plot, the higher the number of cells (x-axis), the higher the number of SG-biased genes (y-axis), in either sex.

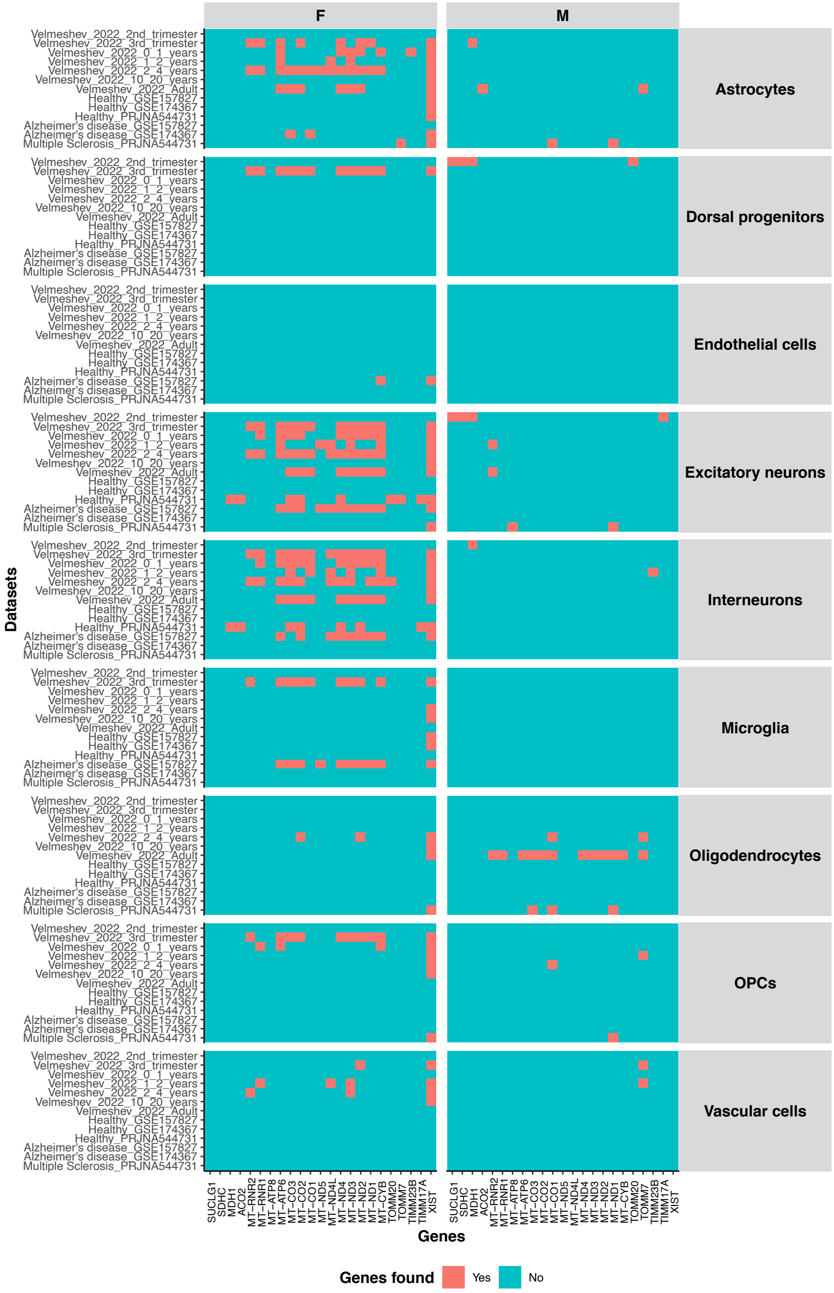
F
M
Astrocytes
Dorsal progenitors
Endothelial cells
Excitatory neurons
Interneurons
Microglia
Oligodendrocytes
OPCs
Vascular cells
Datasets
Velmeshev_2022_2nd_trimester
Velmeshev_2022_3rd_trimester
Velmeshev_2022_0_1_years
Velmeshev_2022_1_2_years
Velmeshev_2022_2_4_years
Velmeshev_2022_10_20_years
Velmeshev_2022_Adult
Healthy_GSE157827
Healthy_GSE174367
Healthy_PRJNA544731
Alzheimer's disease_GSE157827
Alzheimer's disease_GSE174367
Multiple Sclerosis_PRJNA544731
SUCLG1
SDHC
MDH1
ACO2
MT−RNR2
MT−RNR1
MT−ATP8
MT−ATP6
MT−CO3
MT−CO2
MT−CO1
MT−ND5
MT−ND4L
MT−ND4
MT−ND3
MT−ND2
MT−ND1
MT−CYB
TOMM20
TOMM7
TIMM23B
TIMM17A
XIST
Genes
Genes found
Yes
No

Fig. S8: **The mitochondrial (MT) and tricarboxylic acid (TCA) cycle genes are expressed between sexes in a sex-biased manner.** The heatmap displays the presence of MT and TCA genes in SG-biased genes lists from each cell type and dataset. MT, but not TCA or MT housekeeping genes (TIMM, TOMM) are more present in female-biased genes than in males.

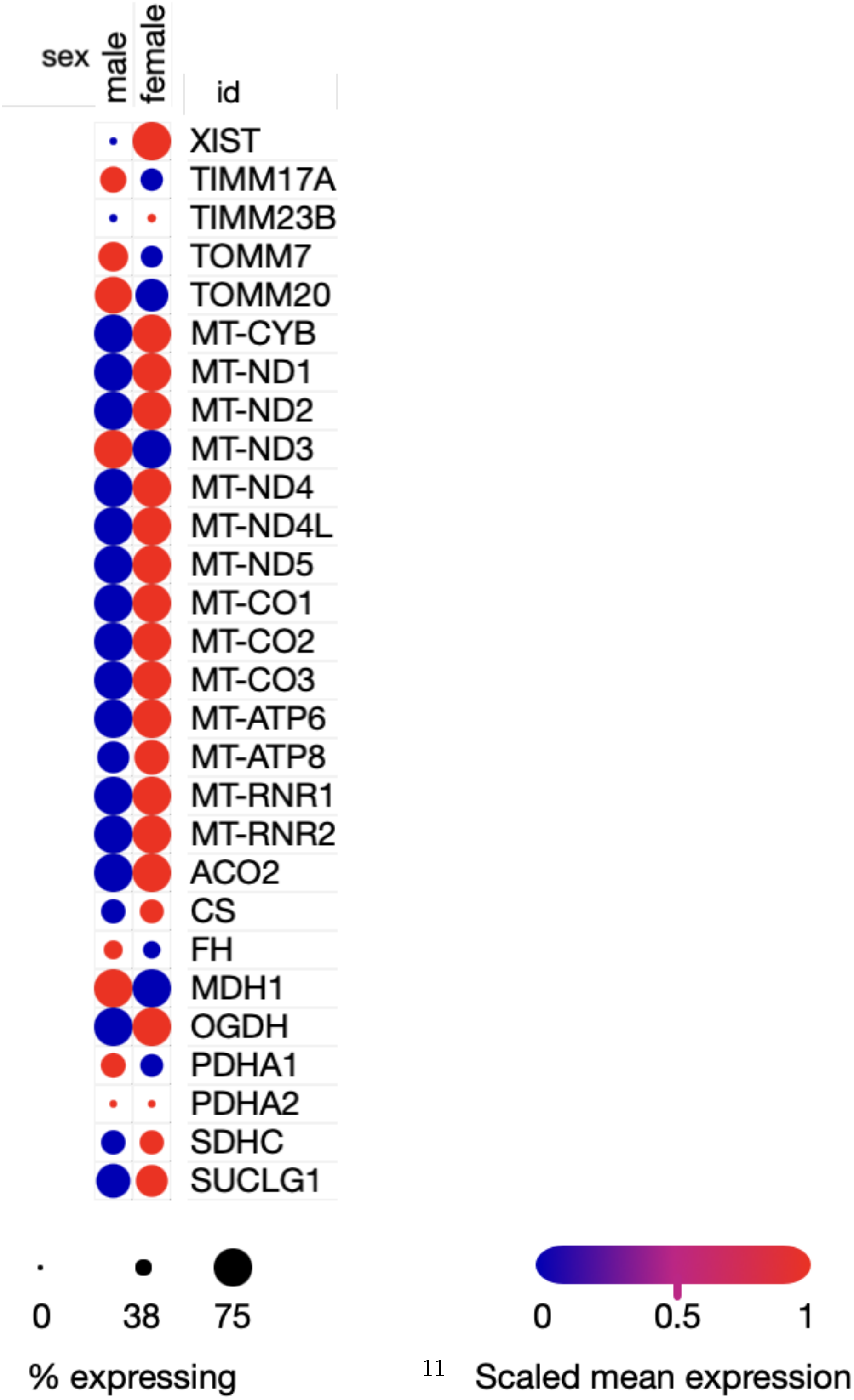
sex
male
female
id
XIST
TIMM17A
TIMM23B
TOMM7
TOMM20
MT-CYB
MT-ND1
MT-ND2
MT-ND3
MT-ND4
MT-ND4L
MT-ND5
MT-CO1
MT-CO2
MT-CO3
MT-ATP6
MT-ATP8
MT-RNR1
MT-RNR2
ACO2
CS
FH
MDH1
OGDH
PDHA1
PDHA2
SDHC
SUCLG1
0
38
75
% expressing
0
0.5
1
Scaled mean expression

Fig. S9: **Mitochondrial genes and the associated nuclear genes are more expressed in females than in males.** This dot plot represents the expression of the same genes we explored regarding mitochondrial female bias in another single-cell study about dopaminergic neurons from Parkinson's disease patients and healthy controls (Kamath et al., 2022), using the Broad Institute Single-Cell Portal.

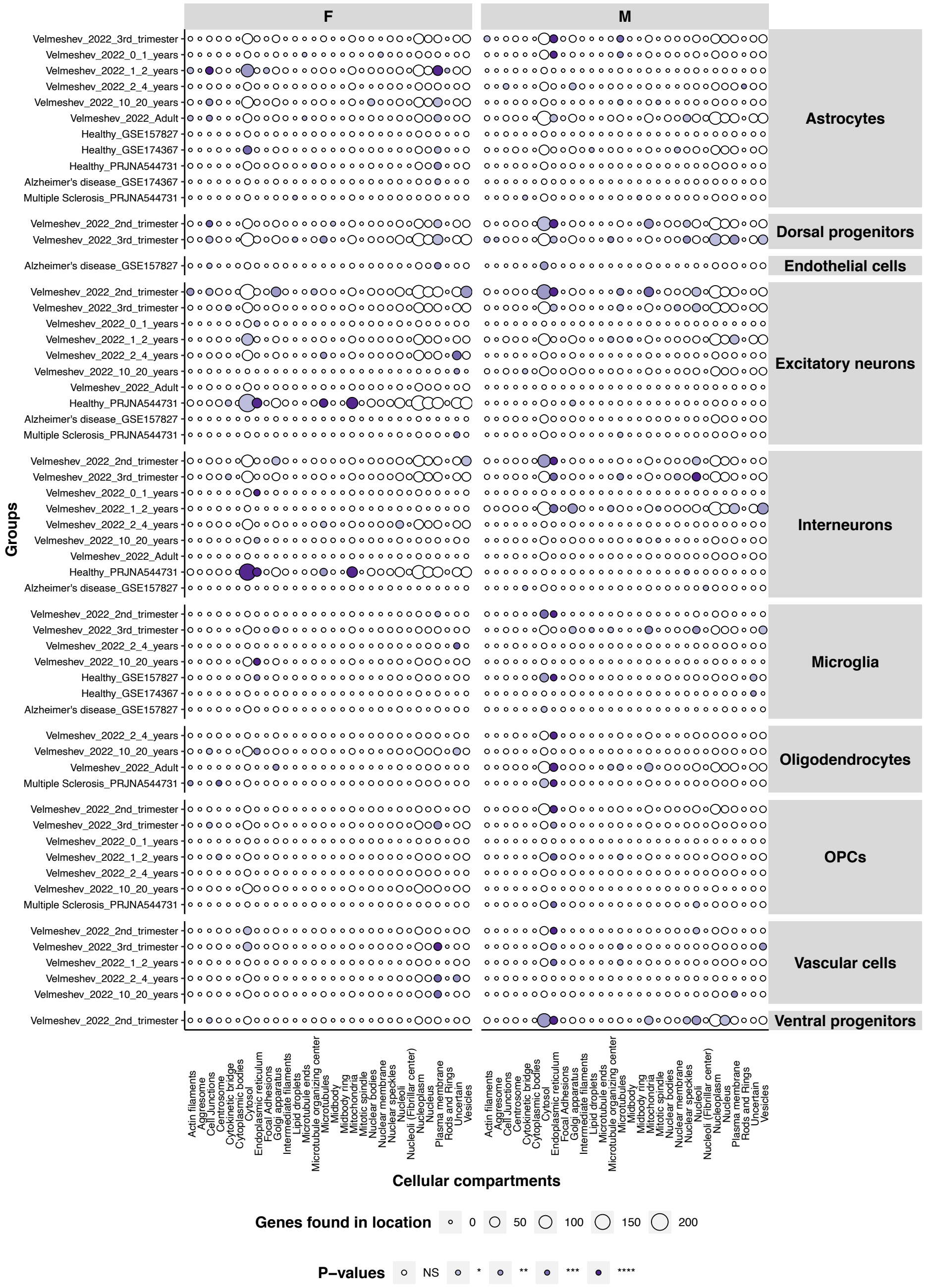
F
M
Groups
Astrocytes
Dorsal progenitors
Endothelial cells
Excitatory neurons
Interneurons
Microglia
Oligodendrocytes
OPCs
Vascular cells
Ventral progenitors
Velmeshev_2022_3rd_trimester
Velmeshev_2022_0_1_years
Velmeshev_2022_1_2_years
Velmeshev_2022_2_4_years
Velmeshev_2022_10_20_years
Velmeshev_2022_Adult
Healthy_GSE157827
Healthy_GSE174367
Healthy_PRJNA544731
Alzheimer's disease_GSE174367
Multiple Sclerosis_PRJNA544731
Velmeshev_2022_2nd_trimester
Alzheimer's disease_GSE157827
Actin filaments
Aggresome
Cell Junctions
Centrosome
Cytokinetic bridge
Cytoplasmic bodies
Cytosol
Endoplasmic reticulum
Focal Adhesions
Golgi apparatus
Intermediate filaments
Lipid droplets
Microtubule ends
Microtubule organizing center
Microtubules
Midbody
Midbody ring
Mitochondria
Mitotic spindle
Nuclear bodies
Nuclear membrane
Nuclear speckles
Nucleoli
Nucleoli (Fibrillar center)
Nucleoplasm
Nucleus
Plasma membrane
Rods and Rings
Uncertain
Vesicles
Cellular compartments
Genes found in location
0
50
100
150
200
P−values
NS
*
**
***
****

Fig. S10: **SG-biased genes are mainly expressed in few cellular compartments, and the distribution is quite similar between the sexes.** This dot plot represents the hyper-geometric enrichment of cellular compartments in each dataset, cell type and sex. The size of the dots indicates the number of SG-biased in each cellular compartment, and the color indicates the p-values after hyper-geometric enrichment. **Legend:** NS: not significant; *: $p<0.05$; **: $p<0.01$; ***: $p<0.001$; ****: $p<0.0001$.

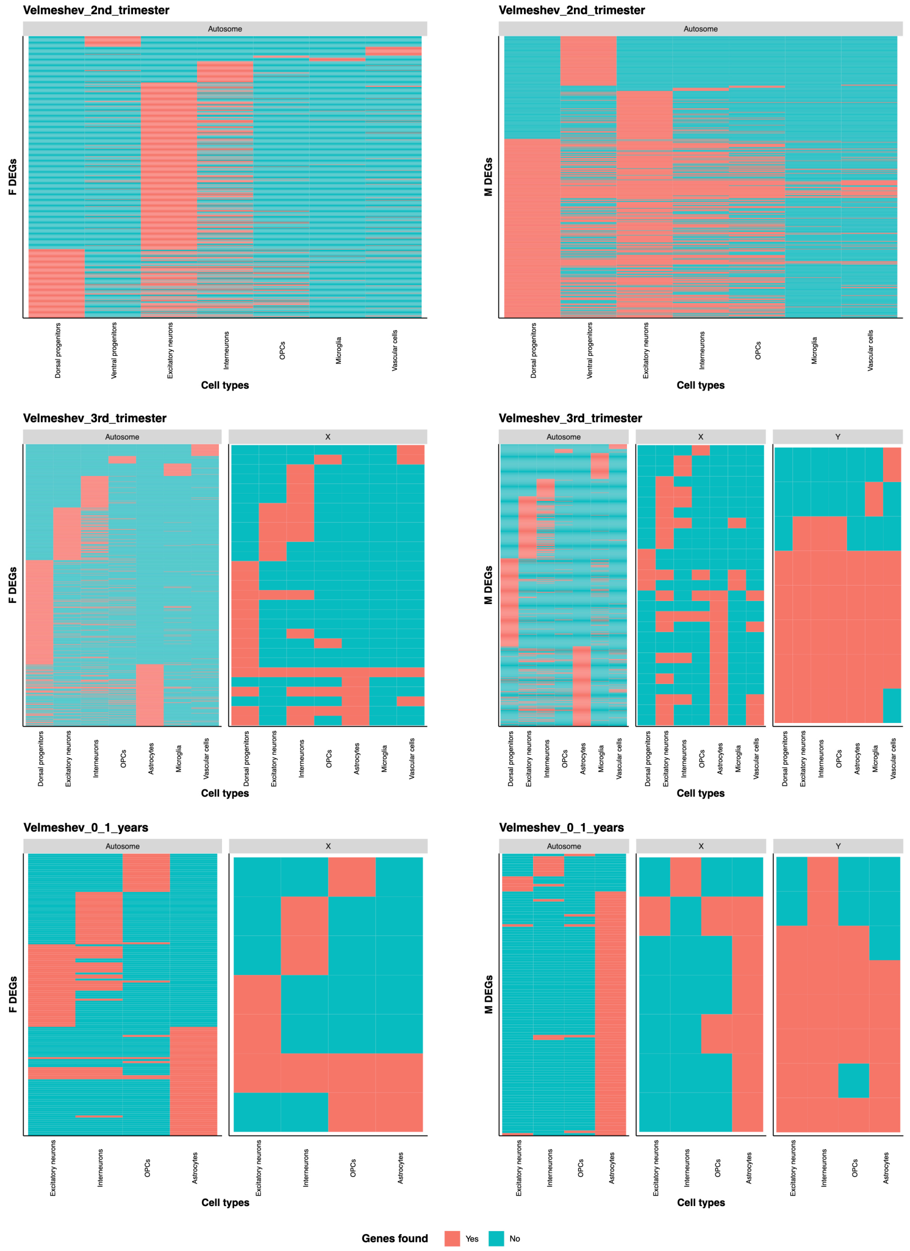
Velmeshev_2nd_trimester
Autosome
F DEGs
Dorsal progenitors
Ventral progenitors
Excitatory neurons
Interneurons
OPCs
Microglia
Vascular cells
Cell types
Velmeshev_2nd_trimester
Autosome
M DEGs
Cell types
Velmeshev_3rd_trimester
Autosome
X
F DEGs
Astrocytes
Cell types
Velmeshev_3rd_trimester
Autosome
X
Y
M DEGs
Cell types
Velmeshev_0_1_years
Autosome
X
F DEGs
Cell types
Velmeshev_0_1_years
Autosome
X
Y
M DEGs
Cell types
Genes found
Yes
No

Fig. S11: **The SG-biased genes are mostly autosomal and cell type-specific**. Heatmaps showing the distribution of SG-biased genes across cell types and sex within each dataset, with the DEGs grouped by chromosome (autosome, X or Y). Presence indicates whether the gene is found in each specific dataset-sex-cell type combination.

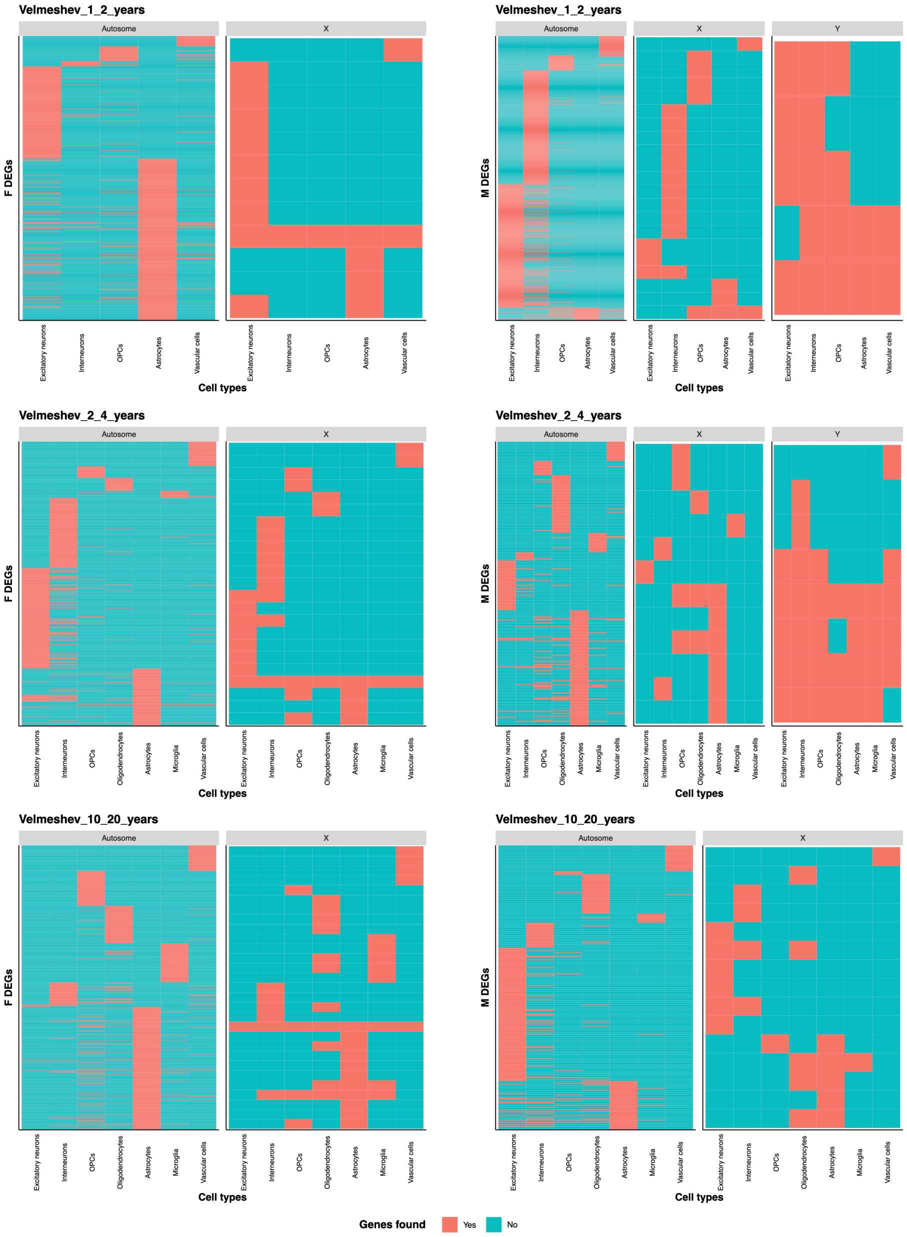

Fig. S12: **The SG-biased genes are mostly autosomal and cell type-specific**. Heatmaps showing the distribution of SG-biased genes across cell types and sex within each dataset, with the DEGs grouped by chromosome (autosome, X or Y). Presence indicates whether the gene is found in each specific dataset-sex-cell type combination.

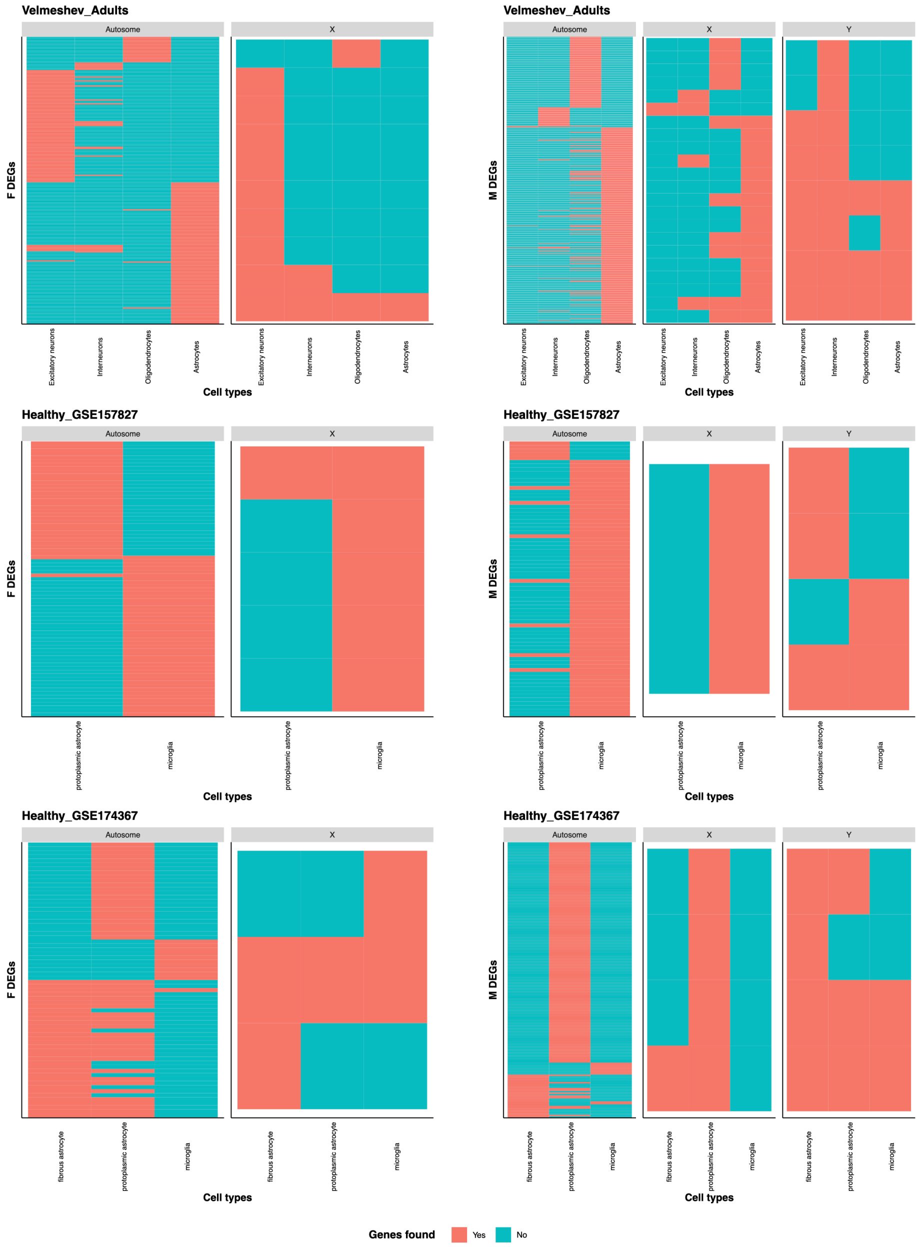

Fig. S13: **The SG-biased genes are mostly autosomal and cell type-specific.** Heatmaps showing the distribution of SG-biased genes across cell types and sex within each dataset, with the DEGs grouped by chromosome (autosome, X or Y). Presence indicates whether the gene is found in each specific dataset-sex-cell type combination.

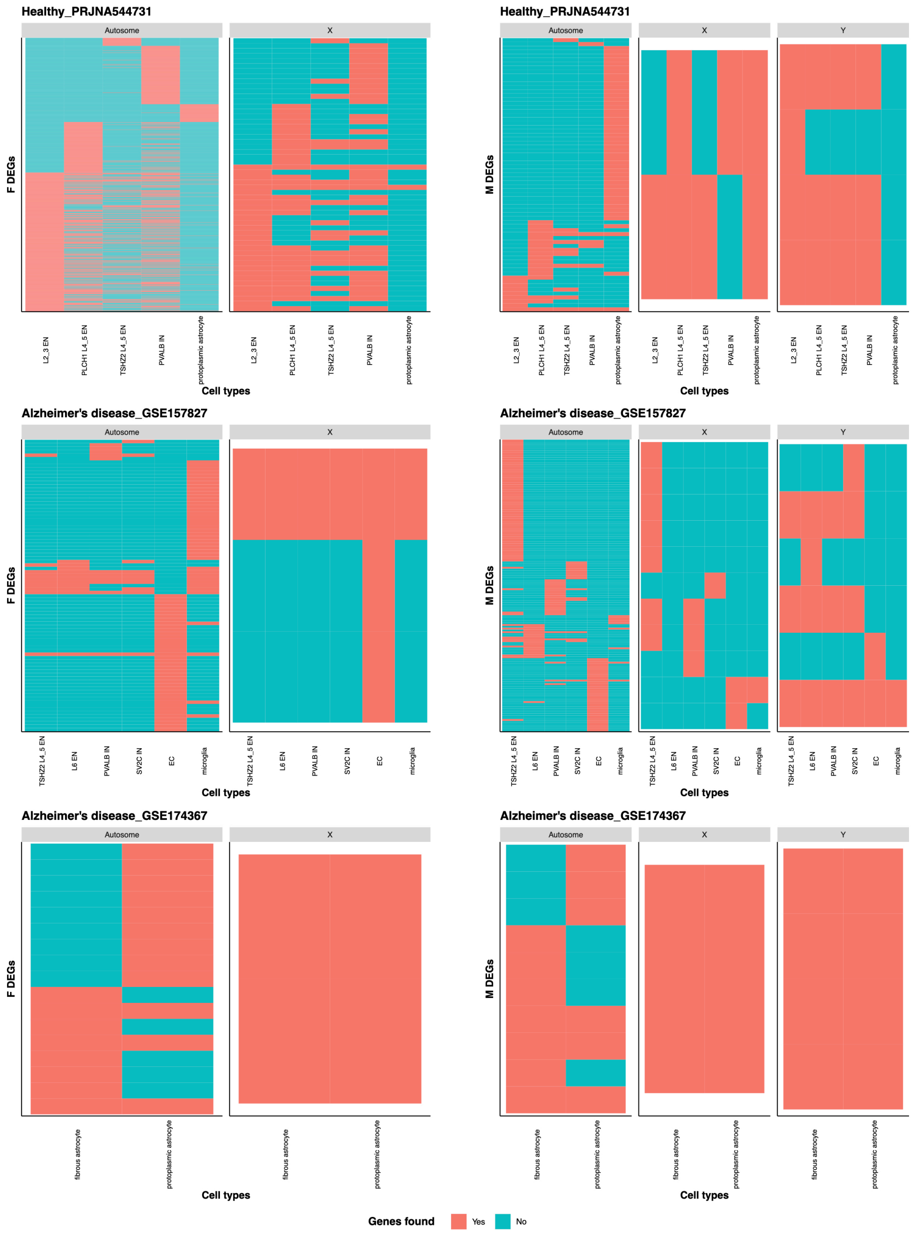

Fig. S14: **The SG-biased genes are mostly autosomal and cell type-specific**. Heatmaps showing the distribution of SG-biased genes across cell types and sex within each dataset, with the DEGs grouped by chromosome (autosome, X or Y). Presence indicates whether the gene is found in each specific dataset-sex-cell type combination.

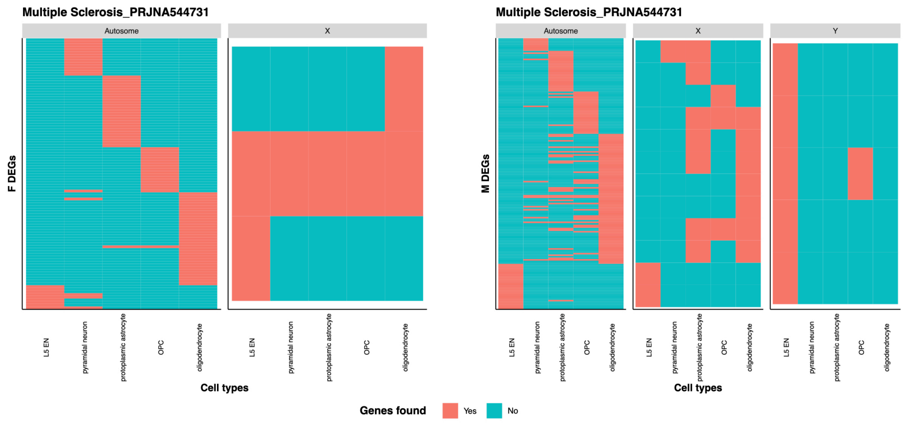

Fig. S15: **The SG-biased genes are mostly autosomal and cell type-specific**. Heatmaps showing the distribution of SG-biased genes across cell types and sex within each dataset, with the DEGs grouped by chromosome (autosome, X or Y). Presence indicates whether the gene is found in each specific dataset-sex-cell type combination.

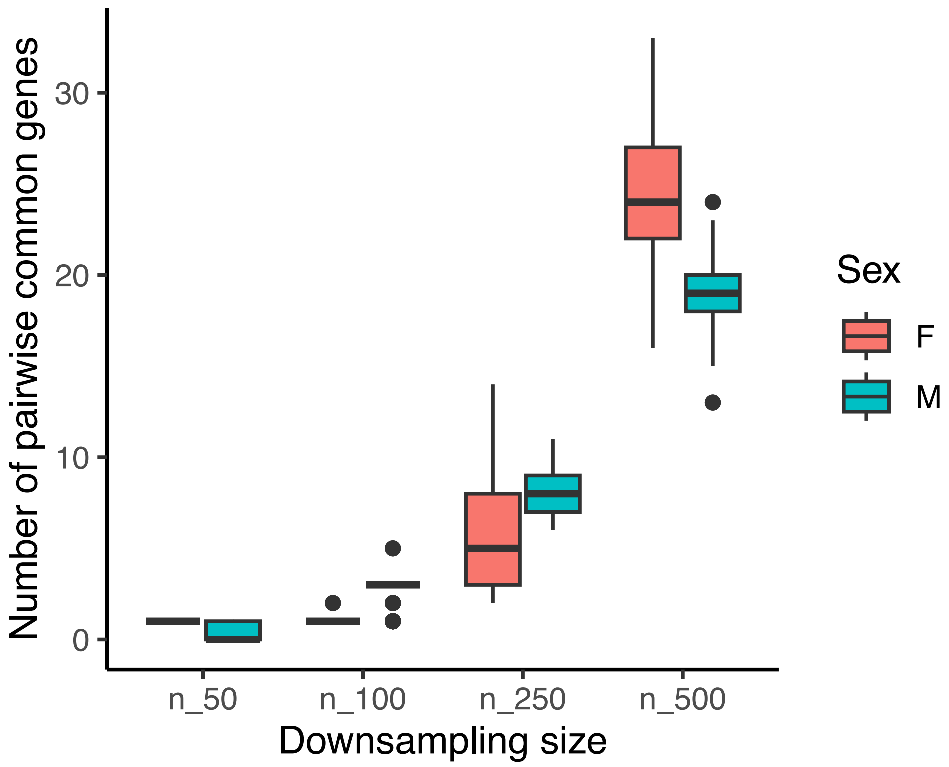

Fig. S16: **Down-sampling of cell populations shows consistency in the found differential genes**. Multiple rounds of down-sampling of excitatory neurons, using of different sizes of sampling, show that while the sampling size affects the number of pairwise common DEGs found, it does not affect the distribution of such comparisons.

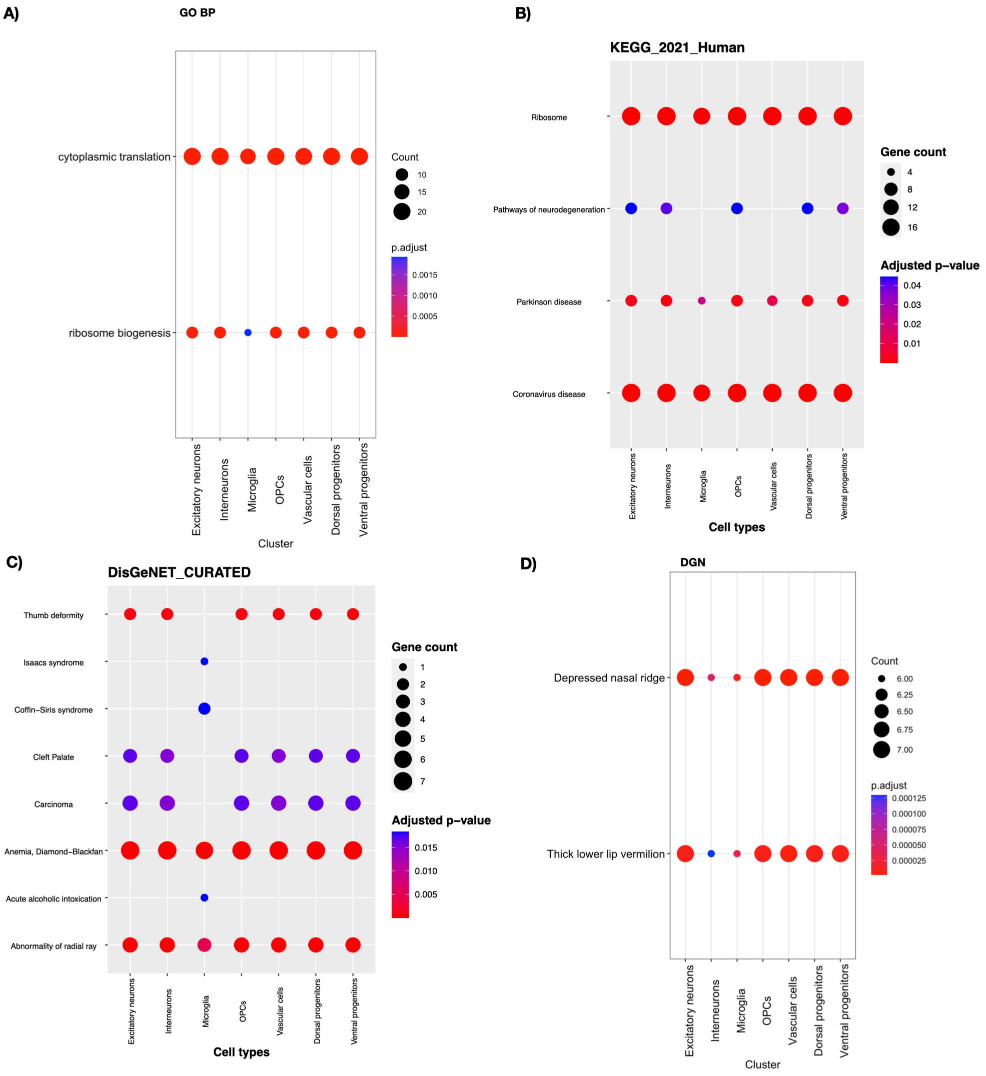

Fig. S17: **Shared male-biased genes in the second trimester showed enrichment for ribosomial and developmental processes**. A-D) Gene ontology biological processes (A), KEGG pathways (B), DisGeNET diseases CURATED (C) and `clusterProfiler` (D) enriched terms in the second trimester in the male-biased genes. The dot size indicate show many genes were found to belong to each GO BP term, and the color is the adjusted p-value (Benjamini-Hochberg correction).

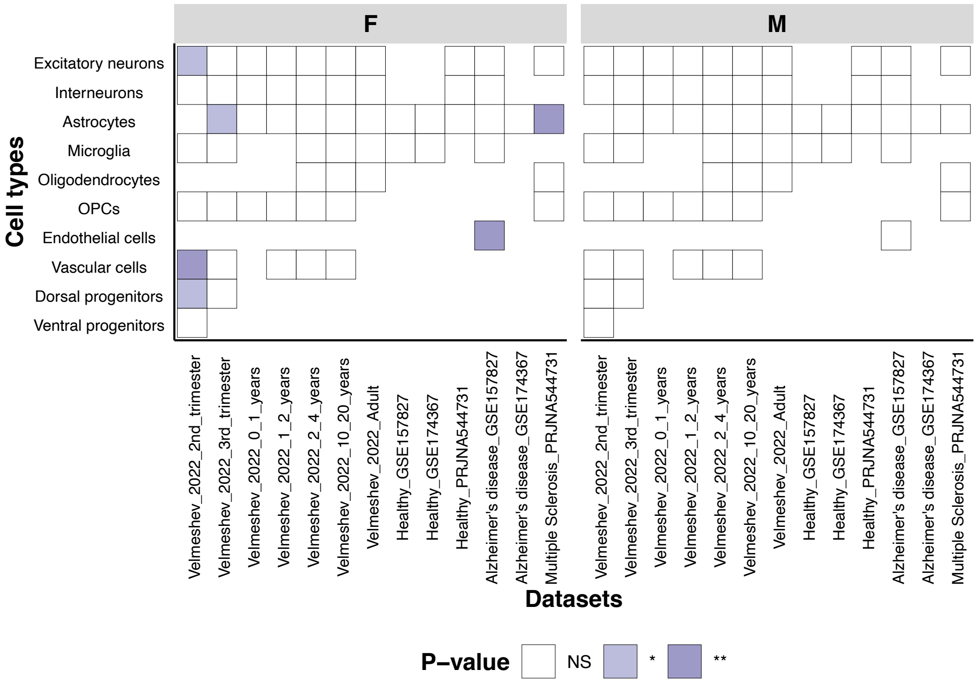

Fig. S18: **Overall, the female-biased genes were more enriched in brain-specific sex-biased DEGs than in the male-biased genes.** This heatmap represents the significant hyper-geometric enrichment of sex-biased DEGs from a bulk RNA-seq study in our SG-biased genes. **Legend**: *: p<0.05; **: p<0.01.

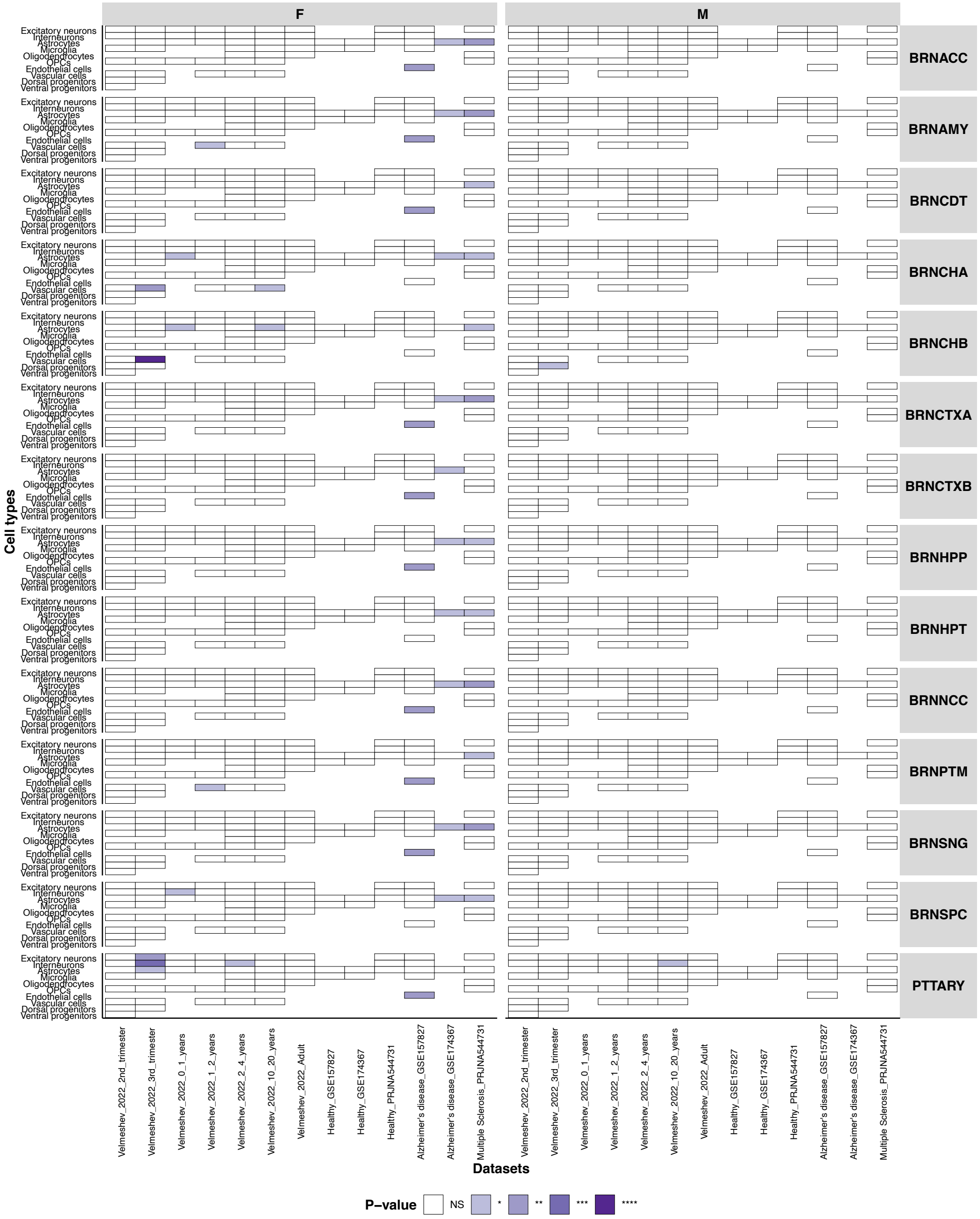
F
M
Cell types
Excitatory neurons
Interneurons
Astrocytes
Microglia
Oligodendrocytes
OPCs
Endothelial cells
Vascular cells
Dorsal progenitors
Ventral progenitors
BRNACC
BRNAMY
BRNCDT
BRNCHA
BRNCHB
BRNCTXA
BRNCTXB
BRNHPP
BRNHPT
BRNNCC
BRNPTM
BRNSNG
BRNSPC
PTTARY
Velmeshev_2022_2nd_trimester
Velmeshev_2022_3rd_trimester
Velmeshev_2022_0_1_years
Velmeshev_2022_1_2_years
Velmeshev_2022_2_4_years
Velmeshev_2022_10_20_years
Velmeshev_2022_Adult
Healthy_GSE157827
Healthy_GSE174367
Healthy_PRJNA544731
Alzheimer's disease_GSE157827
Alzheimer's disease_GSE174367
Multiple Sclerosis_PRJNA544731
Datasets
P-value
NS
*
**
***
****

Fig. S19: **The female-biased genes were more enriched in brain-specific sex-biased DEGs than in the male-biased genes, but no region specificity was observed.** This heatmap represents the significant hyper-geometric enrichment of sex-biased DEGs from a bulk RNA-seq study in our SG-biased genes, divided by brain region. **Legend:** BRNAMY: amygdala; BRNACC: anterior cingulate cortex; BRNCDT: caudate (basal ganglia); BRNCHB: cerebellum; BRNCHA: cerebellum; BRNCTXA: cortex/frontal cortex; BRNCTXB: cortex/frontal cortex; BRNHPP: hippocampus; BRNHPT: hypothalamus; BRNNCC: nucleus accumbens (basal ganglia); BRNPTM: putamen (basal ganglia); BRNSPC: spinal cord; BRNSNG: substantia nigra; PTTARY: pituitary; NS: not significant; *: p<0.05; **: p<0.01; ***: p<0.001; ****: p<0.0001.

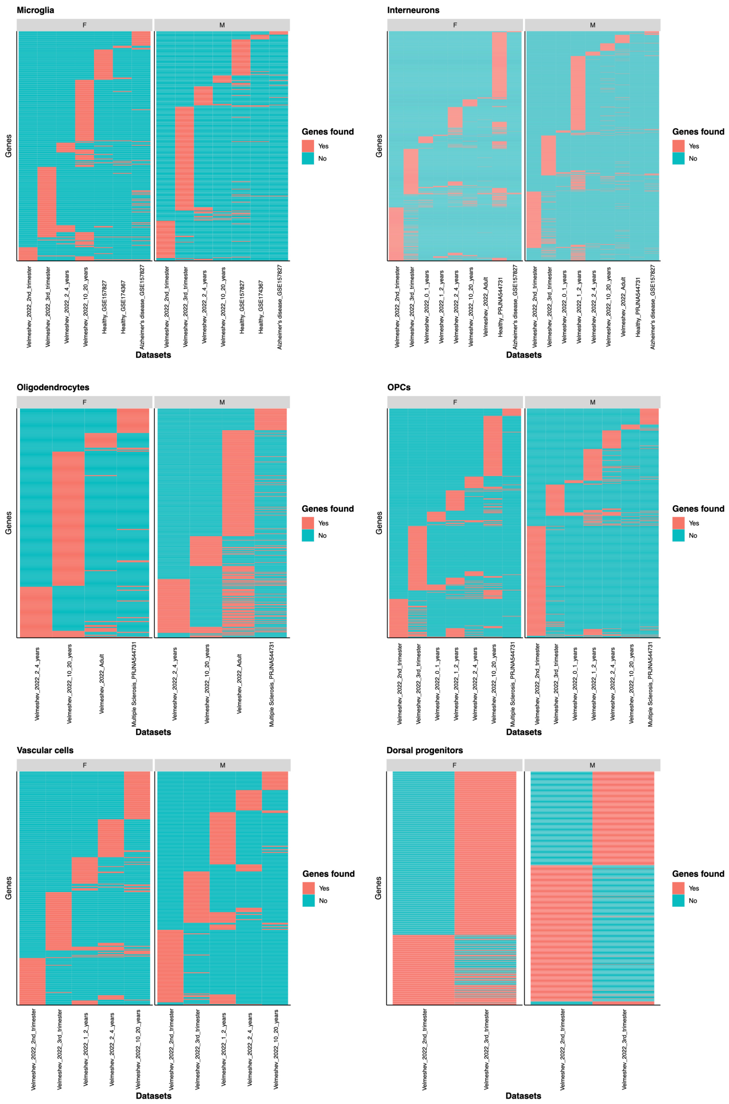

Fig. S20: **SG-biased genes are developmental stage specific, with little overlap between datasets.** This presence heatmap shows whether the SG-biased genes are found in the groups within the same cell type, divided by sex. The heatmaps for endothelial cells and ventral progenitors are missing due to them being present only in one group, AD GSE157827 and third trimester respectively.

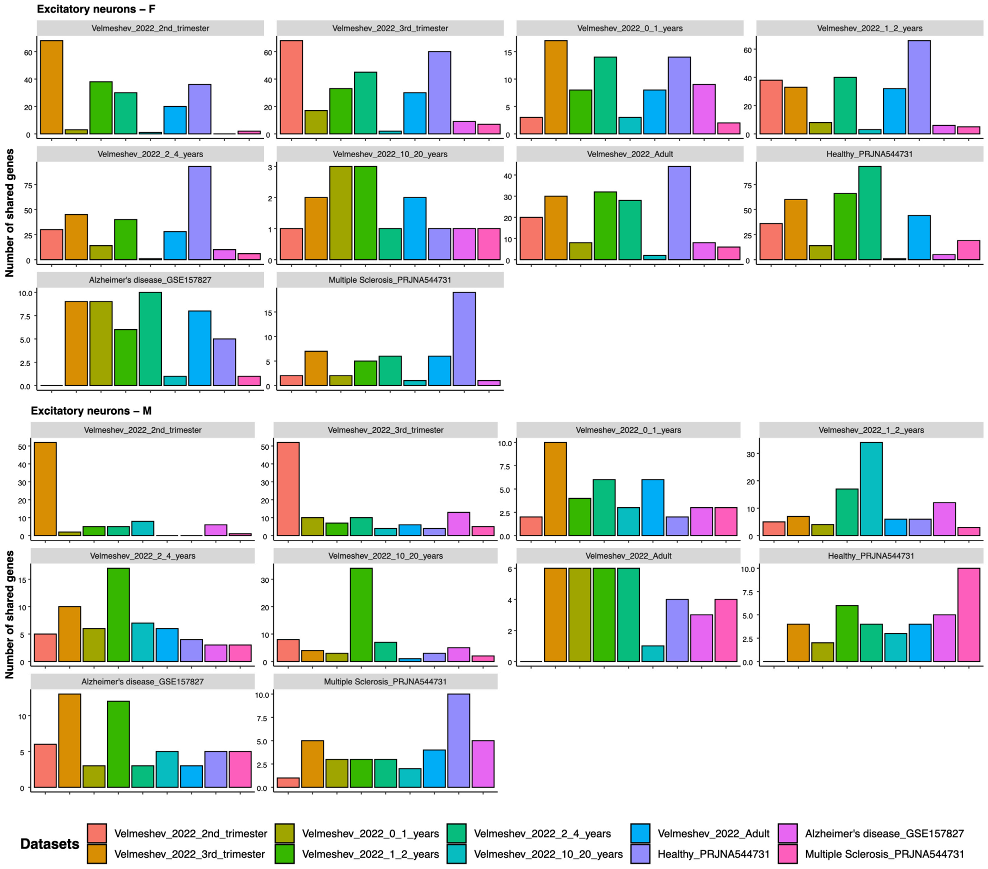

Fig. S21: **SG-biased genes are developmental stage specific, with little overlap between datasets.** The bar plots indicate the number of common SG-biased genes between the reference dataset (facet labels) and all other datasets where the cell type is found.

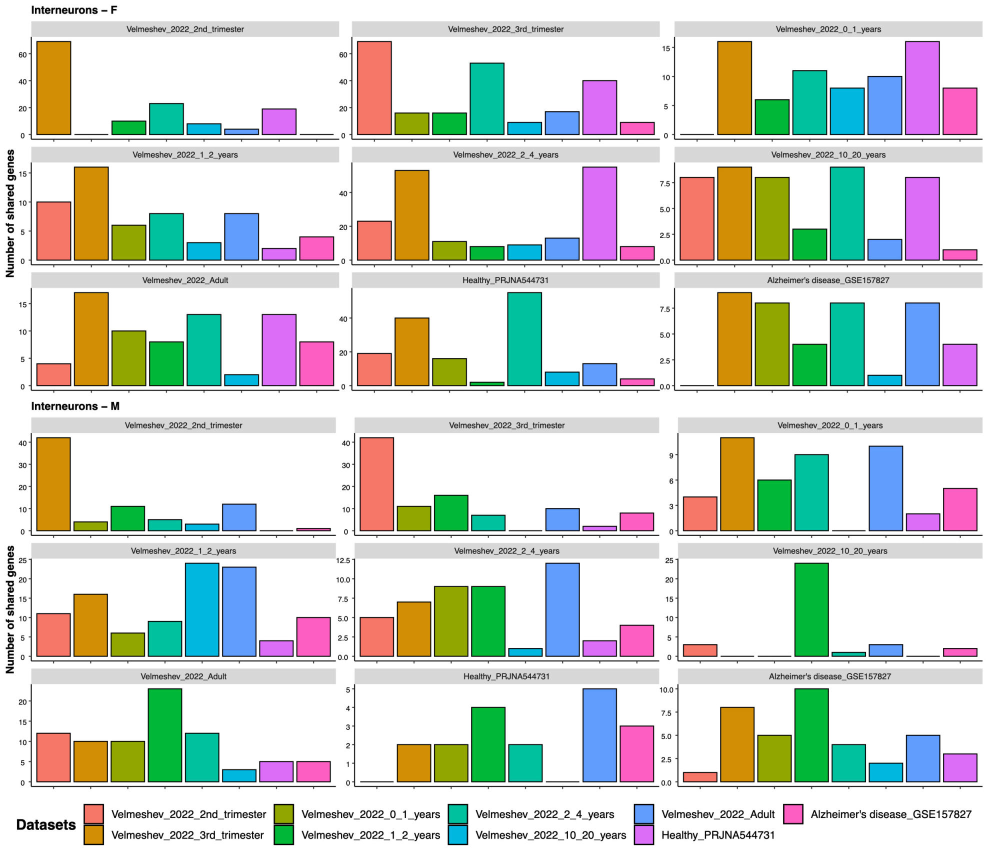

Fig. S22: **SG-biased genes are developmental stage specific, with little overlap between datasets.** The bar plots indicate the number of common SG-biased genes between the reference dataset (facet labels) and all other datasets where the cell type is found.

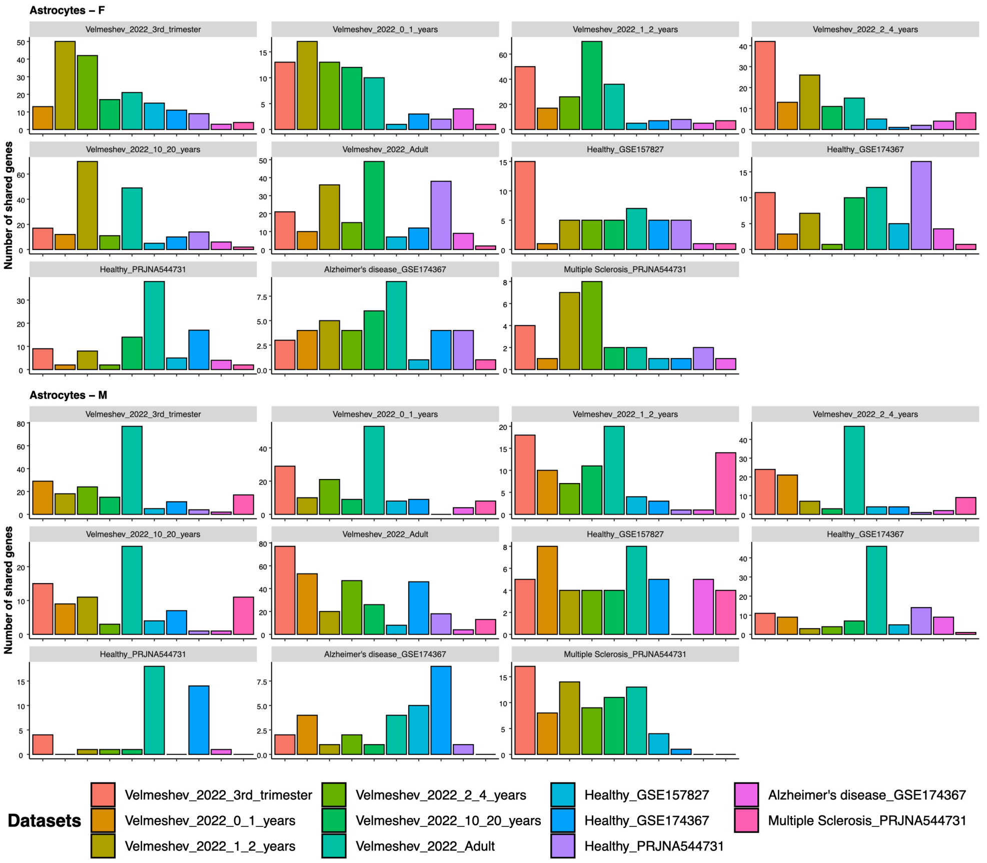

Fig. S23: **SG-biased genes are developmental stage specific, with little overlap between datasets.** The bar plots indicate the number of common SG-biased genes between the reference dataset (facet labels) and all other datasets where the cell type is found.

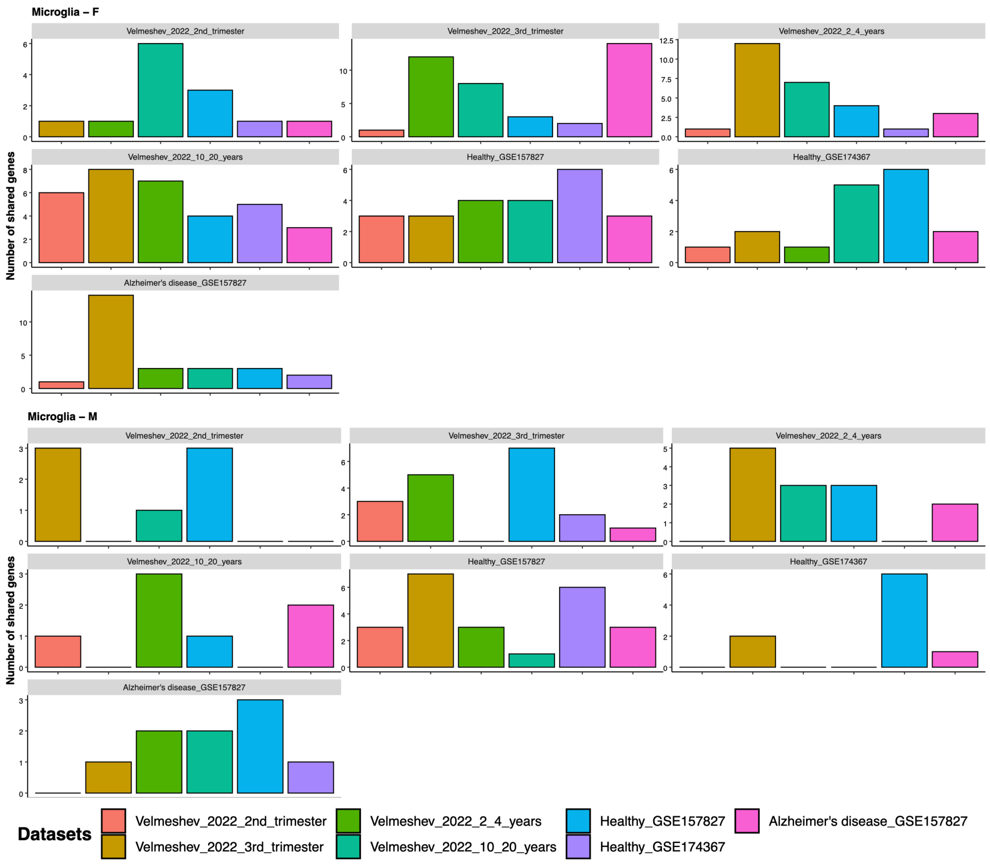

Fig. S24: **SG-biased genes are developmental stage specific, with little overlap between datasets.** The bar plots indicate the number of common SG-biased genes between the reference dataset (facet labels) and all other datasets where the cell type is found.

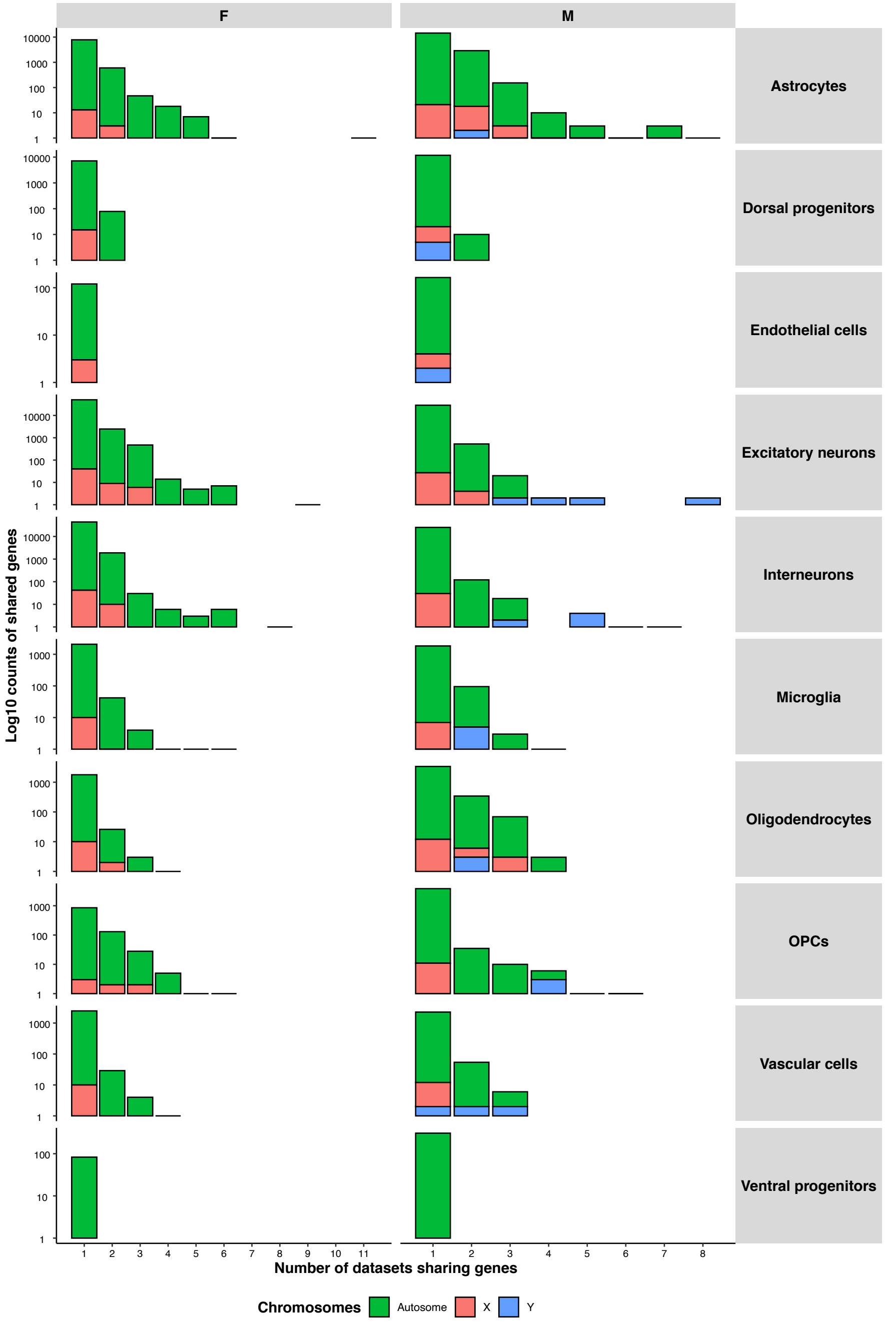
F
M
Astrocytes
Dorsal progenitors
Endothelial cells
Excitatory neurons
Interneurons
Microglia
Oligodendrocytes
OPCs
Vascular cells
Ventral progenitors
Log10 counts of shared genes
Number of datasets sharing genes
Chromosomes
Autosome
X
Y

Fig. S25: **Most of the SG-biased genes belong to the autosome, while the most shared SG-biased genes belong to the sex chromosome instead.** This bar plot shows the distribution of shared SG-biased genes among groups for each cell type, expressed as log10 absolute counts and grouped by chromosome (autosome, X or Y). As the number of groups sharing the SG-biased genes increases, the number of SG-biased genes decreases, and the fraction of these genes which belong to the sex chromosome increases as well.

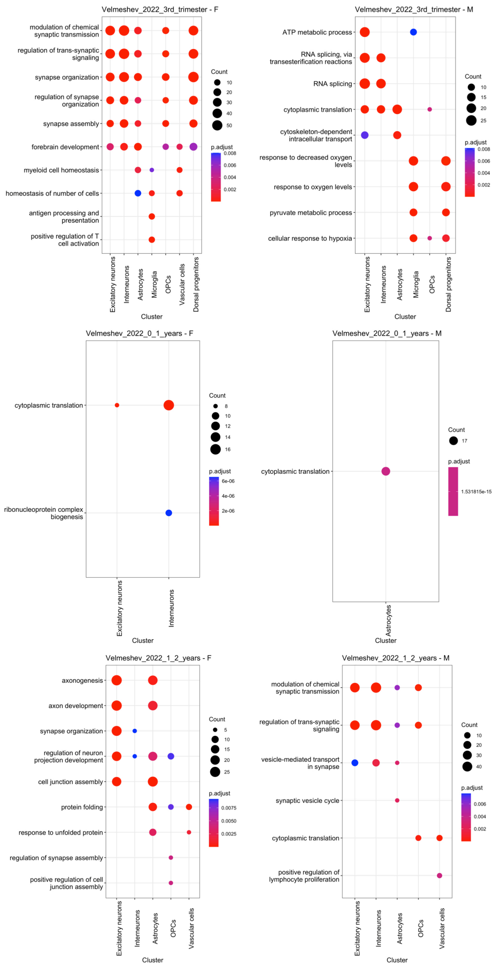

Fig. S26: **Most gene ontology biological processes (GO BPs) were shared across cell types in each dataset-sex combination.** These plots show the GO BPs enrichment in each dataset, in the different cell types and for each sex. The dot size indicate show many genes were found to belong to each GO BP term, and the color is the adjusted p-value (Benjamini-Hochberg correction).

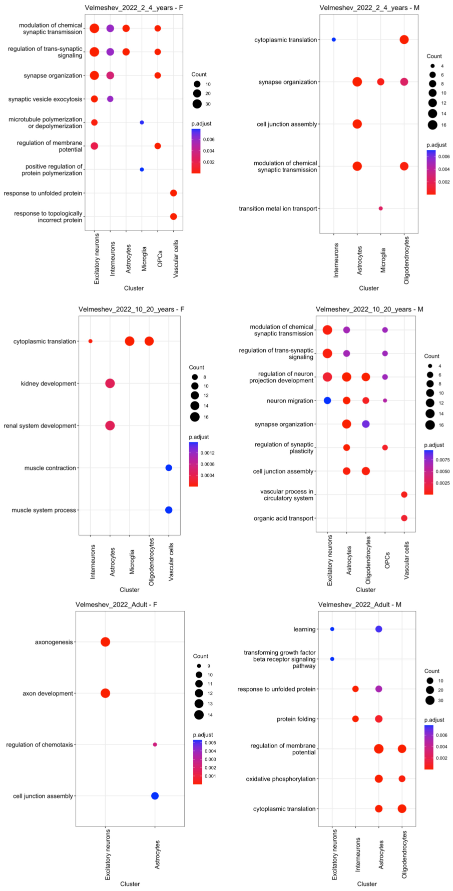

Fig. S27: **Most gene ontology biological processes (GO BPs) were shared across cell types in each dataset-sex combination.** These plots show the GO BPs enrichment in each dataset, in the different cell types and for each sex. The dot size indicate show many genes were found to belong to each GO BP term, and the color is the adjusted p-value (Benjamini-Hochberg correction).

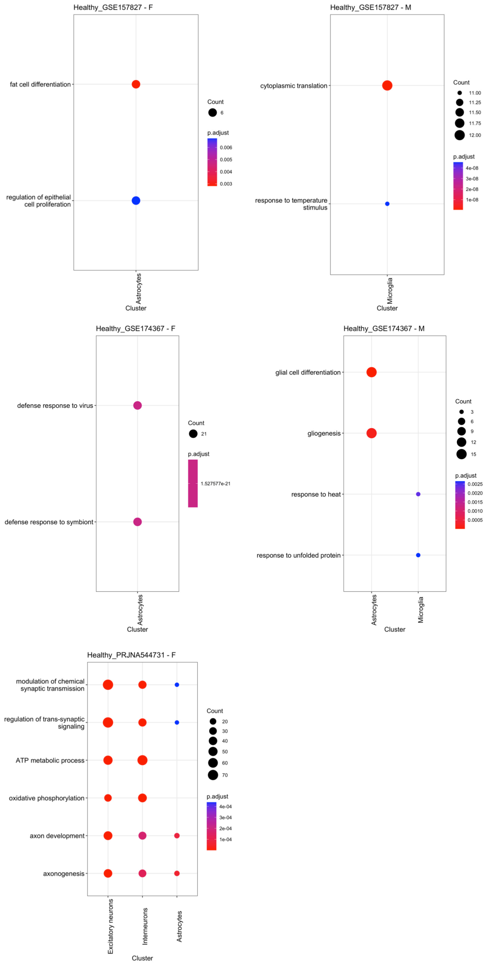

Fig. S28: **Most gene ontology biological processes (GO BPs) were shared across cell types in each dataset-sex combination.** These plots show the GO BPs enrichment in each dataset, in the different cell types and for each sex. The dot size indicate show many genes were found to belong to each GO BP term, and the color is the adjusted p-value (Benjamini-Hochberg correction).

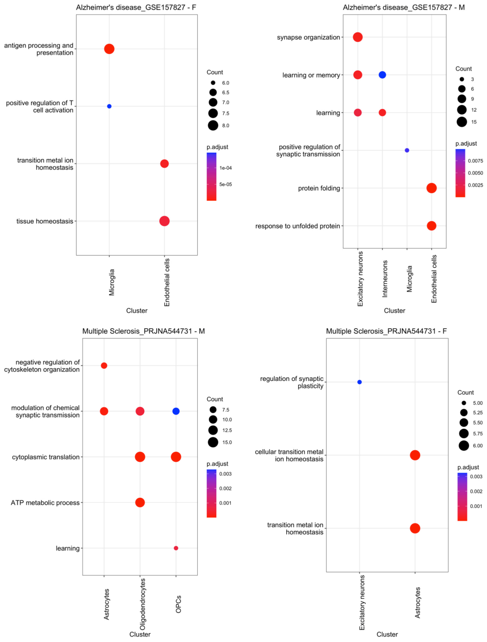

Fig. S29: **Most gene ontology biological processes (GO BPs) were shared across cell types in each dataset-sex combination.** These plots show the GO BPs enrichment in each dataset, in the different cell types and for each sex. The dot size indicate show many genes were found to belong to each GO BP term, and the color is the adjusted p-value (Benjamini-Hochberg correction).

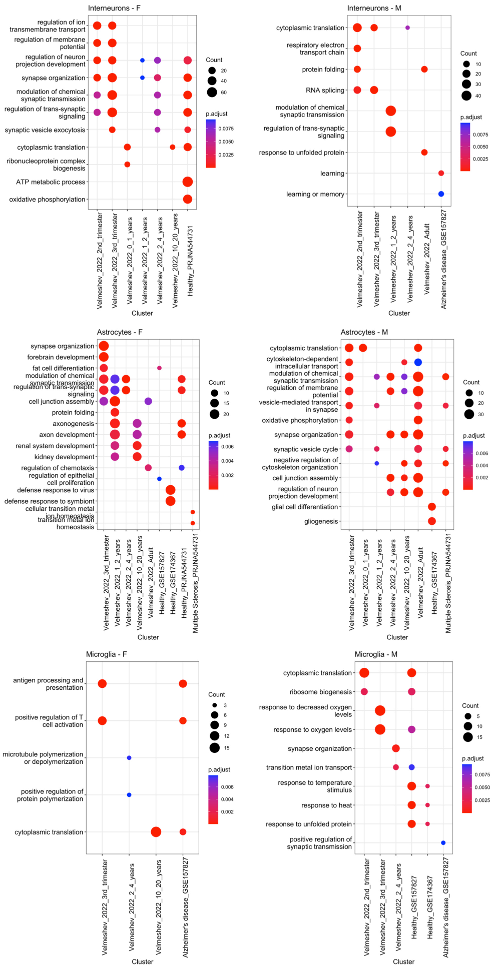

Fig. S30: **Most gene ontology biological processes (GO BPs) were shared across datasets in each cell type-sex combination.** These plots show the GO BPs enrichment in each cell type, across datasets and for each sex. The dot size indicate show many genes were found to belong to each GO BP term, and the color is the adjusted p-value (Benjamini-Hochberg correction).

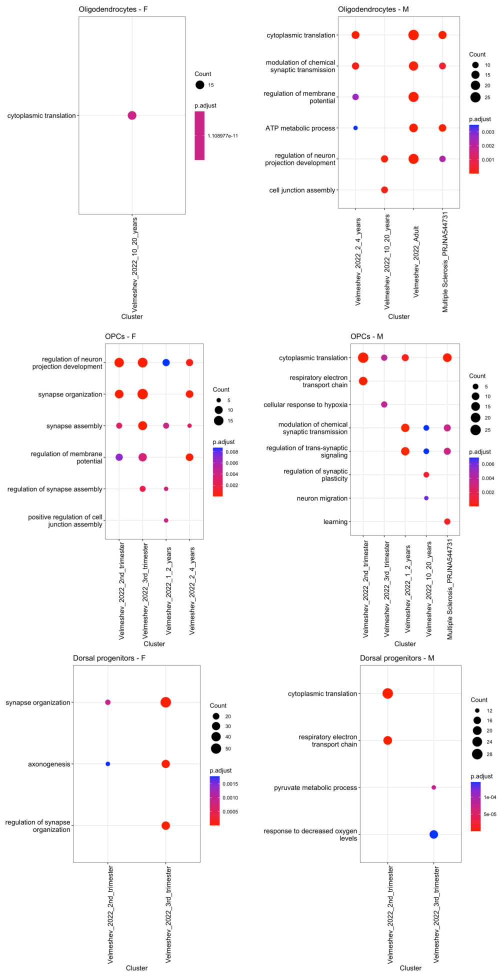

Fig. S31: **Most gene ontology biological processes (GO BPs) were shared across datasets in each cell type-sex combination.** These plots show the GO BPs enrichment in each cell type, across datasets and for each sex. The dot size indicate show many genes were found to belong to each GO BP term, and the color is the adjusted p-value (Benjamini-Hochberg correction).

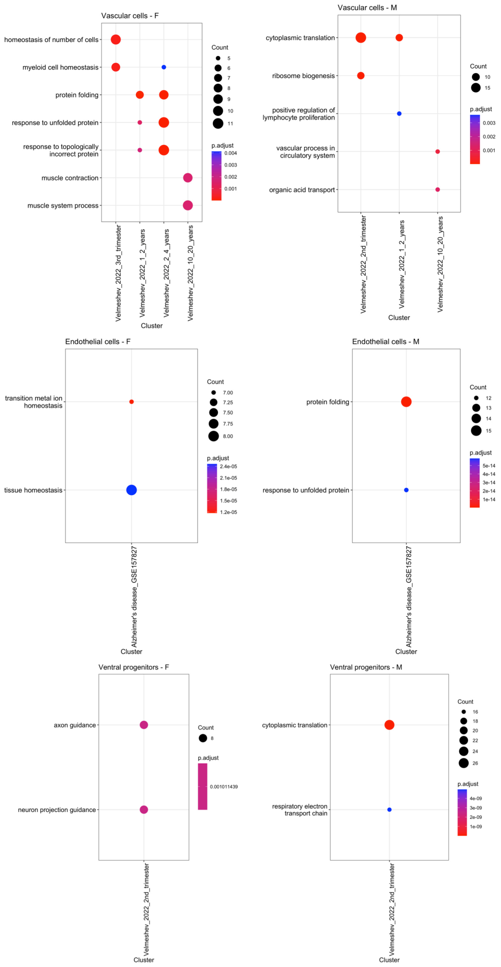

Fig. S32: **Most gene ontology biological processes (GO BPs) were shared across datasets in each cell type-sex combination.** These plots show the GO BPs enrichment in each cell type, across datasets and for each sex. The dot size indicate show many genes were found to belong to each GO BP term, and the color is the adjusted p-value (Benjamini-Hochberg correction).

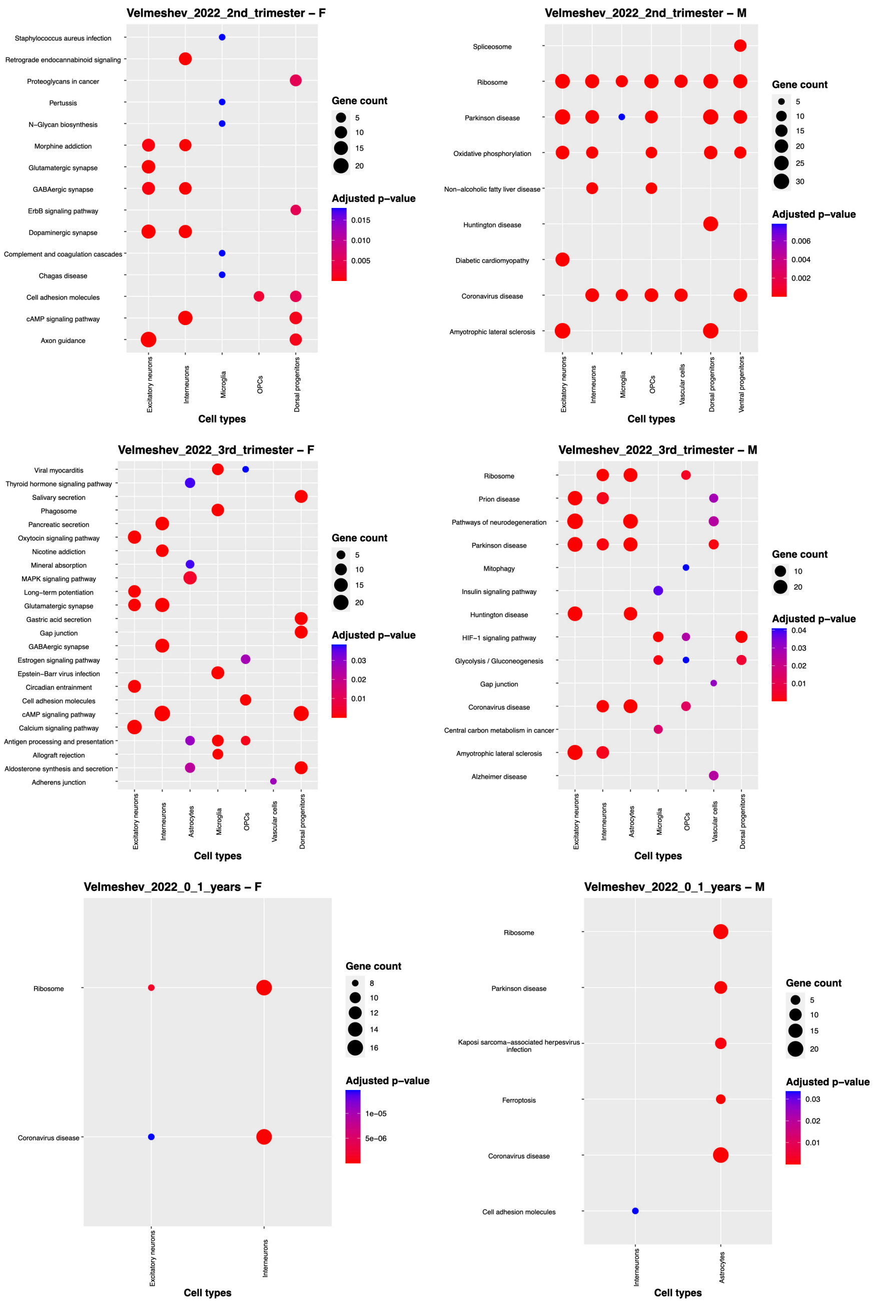
Velmeshev_2022_2nd_trimester – F
Staphylococcus aureus infection
Retrograde endocannabinoid signaling
Proteoglycans in cancer
Pertussis
N−Glycan biosynthesis
Morphine addiction
Glutamatergic synapse
GABAergic synapse
ErbB signaling pathway
Dopaminergic synapse
Complement and coagulation cascades
Chagas disease
Cell adhesion molecules
cAMP signaling pathway
Axon guidance
Excitatory neurons
Interneurons
Microglia
OPCs
Dorsal progenitors
Cell types
Gene count
5
10
15
20
Adjusted p−value
0.015
0.010
0.005
Velmeshev_2022_2nd_trimester – M
Spliceosome
Ribosome
Parkinson disease
Oxidative phosphorylation
Non−alcoholic fatty liver disease
Huntington disease
Diabetic cardiomyopathy
Coronavirus disease
Amyotrophic lateral sclerosis
Excitatory neurons
Interneurons
Microglia
OPCs
Vascular cells
Dorsal progenitors
Ventral progenitors
Cell types
Gene count
5
10
15
20
25
30
Adjusted p−value
0.006
0.004
0.002
Velmeshev_2022_3rd_trimester – F
Viral myocarditis
Thyroid hormone signaling pathway
Salivary secretion
Phagosome
Pancreatic secretion
Oxytocin signaling pathway
Nicotine addiction
Mineral absorption
MAPK signaling pathway
Long−term potentiation
Glutamatergic synapse
Gastric acid secretion
Gap junction
GABAergic synapse
Estrogen signaling pathway
Epstein−Barr virus infection
Circadian entrainment
Cell adhesion molecules
cAMP signaling pathway
Calcium signaling pathway
Antigen processing and presentation
Allograft rejection
Aldosterone synthesis and secretion
Adherens junction
Excitatory neurons
Interneurons
Astrocytes
Microglia
OPCs
Vascular cells
Dorsal progenitors
Cell types
Gene count
5
10
15
20
Adjusted p−value
0.03
0.02
0.01
Velmeshev_2022_3rd_trimester – M
Ribosome
Prion disease
Pathways of neurodegeneration
Parkinson disease
Mitophagy
Insulin signaling pathway
Huntington disease
HIF−1 signaling pathway
Glycolysis / Gluconeogenesis
Gap junction
Coronavirus disease
Central carbon metabolism in cancer
Amyotrophic lateral sclerosis
Alzheimer disease
Excitatory neurons
Interneurons
Astrocytes
Microglia
OPCs
Vascular cells
Dorsal progenitors
Cell types
Gene count
10
20
Adjusted p−value
0.04
0.03
0.02
0.01
Velmeshev_2022_0_1_years – F
Ribosome
Coronavirus disease
Excitatory neurons
Interneurons
Cell types
Gene count
8
10
12
14
16
Adjusted p−value
1e−05
5e−06
Velmeshev_2022_0_1_years – M
Ribosome
Parkinson disease
Kaposi sarcoma−associated herpesvirus infection
Ferroptosis
Coronavirus disease
Cell adhesion molecules
Interneurons
Astrocytes
Cell types
Gene count
5
10
15
20
Adjusted p−value
0.03
0.02
0.01

Fig. S33: **Most Kyoto Encyclopedia of Genes and Genomes (KEGG) pathways were shared across cell types in each dataset-sex combination.** These plots show the KEGG pathways enrichment in each dataset, across cell types and for each sex. The dot size indicate show many genes were found to belong to each pathway, and the color is the adjusted p-value (Benjamini-Hochberg correction).

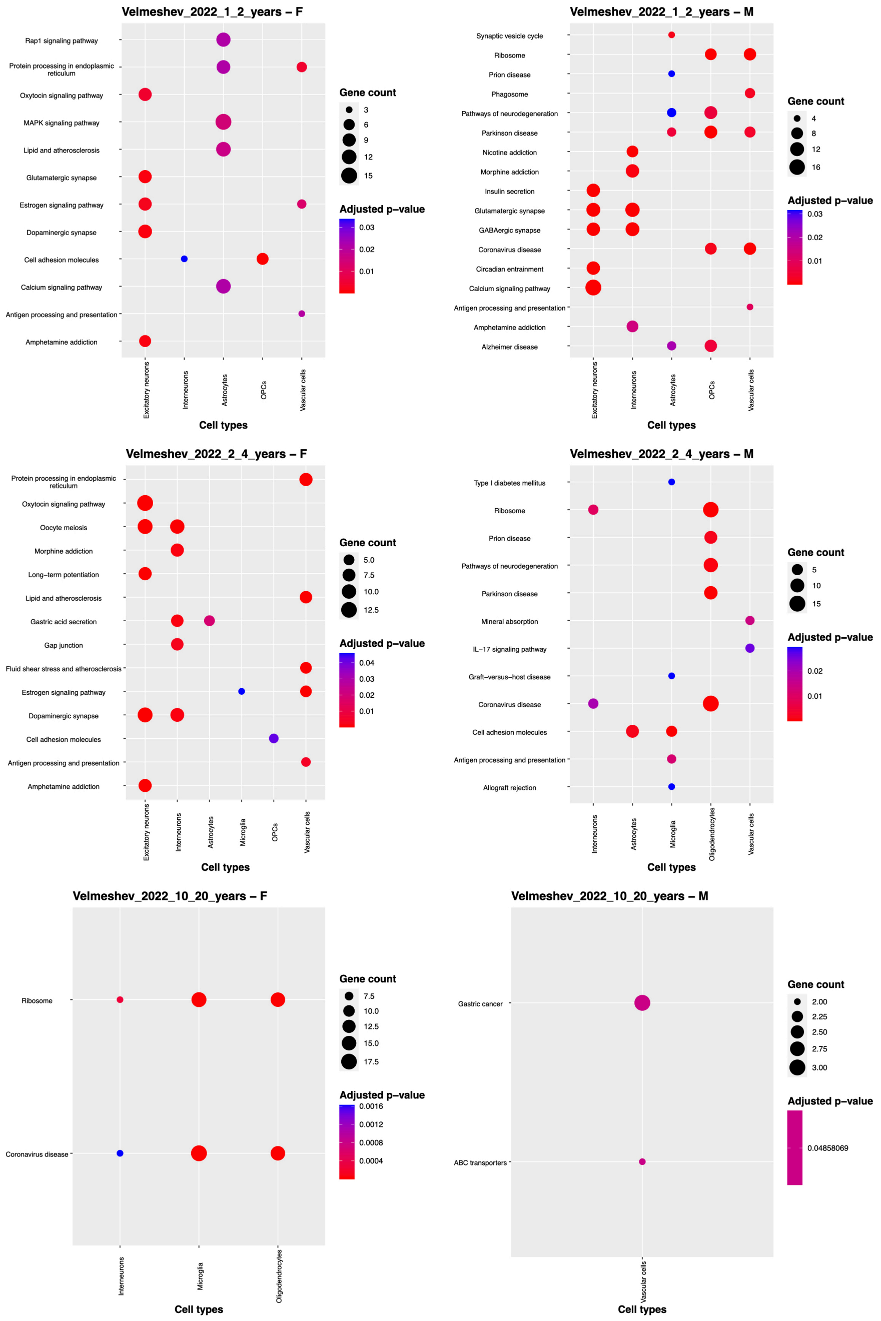
Velmeshev_2022_1_2_years – F
Rap1 signaling pathway
Protein processing in endoplasmic reticulum
Oxytocin signaling pathway
MAPK signaling pathway
Lipid and atherosclerosis
Glutamatergic synapse
Estrogen signaling pathway
Dopaminergic synapse
Cell adhesion molecules
Calcium signaling pathway
Antigen processing and presentation
Amphetamine addiction
Excitatory neurons
Interneurons
Astrocytes
OPCs
Vascular cells
Cell types
Gene count
3
6
9
12
15
Adjusted p−value
0.03
0.02
0.01
Velmeshev_2022_1_2_years – M
Synaptic vesicle cycle
Ribosome
Prion disease
Phagosome
Pathways of neurodegeneration
Parkinson disease
Nicotine addiction
Morphine addiction
Insulin secretion
Glutamatergic synapse
GABAergic synapse
Coronavirus disease
Circadian entrainment
Calcium signaling pathway
Antigen processing and presentation
Amphetamine addiction
Alzheimer disease
Excitatory neurons
Interneurons
Astrocytes
OPCs
Vascular cells
Cell types
Gene count
4
8
12
16
Adjusted p−value
0.03
0.02
0.01
Velmeshev_2022_2_4_years – F
Protein processing in endoplasmic reticulum
Oxytocin signaling pathway
Oocyte meiosis
Morphine addiction
Long−term potentiation
Lipid and atherosclerosis
Gastric acid secretion
Gap junction
Fluid shear stress and atherosclerosis
Estrogen signaling pathway
Dopaminergic synapse
Cell adhesion molecules
Antigen processing and presentation
Amphetamine addiction
Excitatory neurons
Interneurons
Astrocytes
Microglia
OPCs
Vascular cells
Cell types
Gene count
5.0
7.5
10.0
12.5
Adjusted p−value
0.04
0.03
0.02
0.01
Velmeshev_2022_2_4_years – M
Type I diabetes mellitus
Ribosome
Prion disease
Pathways of neurodegeneration
Parkinson disease
Mineral absorption
IL−17 signaling pathway
Graft−versus−host disease
Coronavirus disease
Cell adhesion molecules
Antigen processing and presentation
Allograft rejection
Interneurons
Astrocytes
Microglia
Oligodendrocytes
Vascular cells
Cell types
Gene count
5
10
15
Adjusted p−value
0.02
0.01
Velmeshev_2022_10_20_years – F
Ribosome
Coronavirus disease
Interneurons
Microglia
Oligodendrocytes
Cell types
Gene count
7.5
10.0
12.5
15.0
17.5
Adjusted p−value
0.0016
0.0012
0.0008
0.0004
Velmeshev_2022_10_20_years – M
Gastric cancer
ABC transporters
Vascular cells
Cell types
Gene count
2.00
2.25
2.50
2.75
3.00
Adjusted p−value
0.04858069

Fig. S34: **Most Kyoto Encyclopedia of Genes and Genomes (KEGG) pathways were shared across cell types in each dataset-sex combination.** These plots show the KEGG pathways enrichment in each dataset, across cell types and for each sex. The dot size indicate show many genes were found to belong to each pathway, and the color is the adjusted p-value (Benjamini-Hochberg correction).

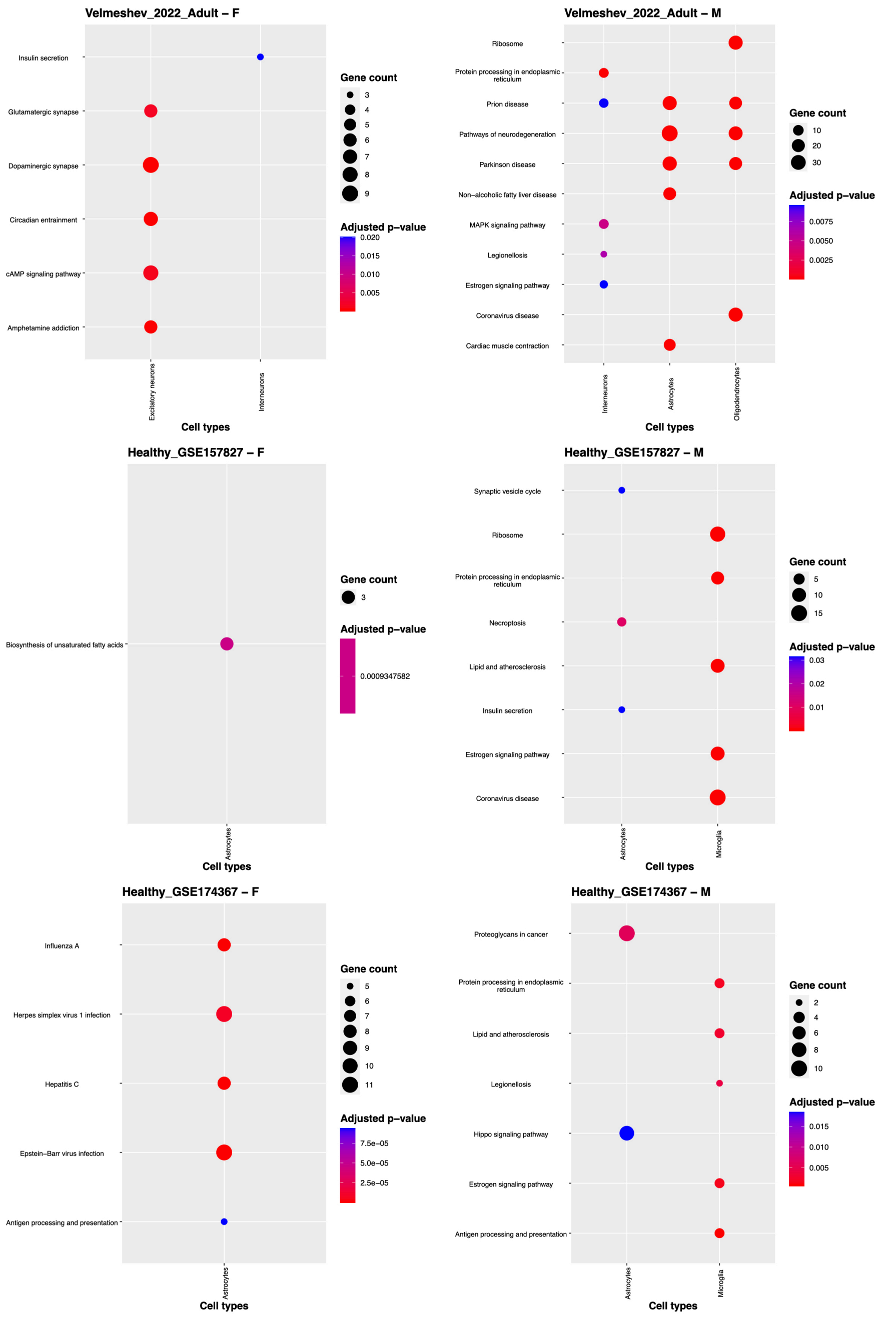
Velmeshev_2022_Adult – F
Insulin secretion
Glutamatergic synapse
Dopaminergic synapse
Circadian entrainment
cAMP signaling pathway
Amphetamine addiction
Excitatory neurons
Interneurons
Cell types
Gene count
Adjusted p–value
Velmeshev_2022_Adult – M
Ribosome
Protein processing in endoplasmic reticulum
Prion disease
Pathways of neurodegeneration
Parkinson disease
Non–alcoholic fatty liver disease
MAPK signaling pathway
Legionellosis
Estrogen signaling pathway
Coronavirus disease
Cardiac muscle contraction
Interneurons
Astrocytes
Oligodendrocytes
Cell types
Gene count
Adjusted p–value
Healthy_GSE157827 – F
Biosynthesis of unsaturated fatty acids
Astrocytes
Cell types
Gene count
Adjusted p–value
0.0009347582
Healthy_GSE157827 – M
Synaptic vesicle cycle
Ribosome
Protein processing in endoplasmic reticulum
Necroptosis
Lipid and atherosclerosis
Insulin secretion
Estrogen signaling pathway
Coronavirus disease
Astrocytes
Microglia
Cell types
Gene count
Adjusted p–value
Healthy_GSE174367 – F
Influenza A
Herpes simplex virus 1 infection
Hepatitis C
Epstein–Barr virus infection
Antigen processing and presentation
Astrocytes
Cell types
Gene count
Adjusted p–value
7.5e–05
5.0e–05
2.5e–05
Healthy_GSE174367 – M
Proteoglycans in cancer
Protein processing in endoplasmic reticulum
Lipid and atherosclerosis
Legionellosis
Hippo signaling pathway
Estrogen signaling pathway
Antigen processing and presentation
Astrocytes
Microglia
Cell types
Gene count
Adjusted p–value

Fig. S35: **Most Kyoto Encyclopedia of Genes and Genomes (KEGG) pathways were shared across cell types in each dataset-sex combination.** These plots show the KEGG pathways enrichment in each dataset, across cell types and for each sex. The dot size indicate show many genes were found to belong to each pathway, and the color is the adjusted p-value (Benjamini-Hochberg correction).

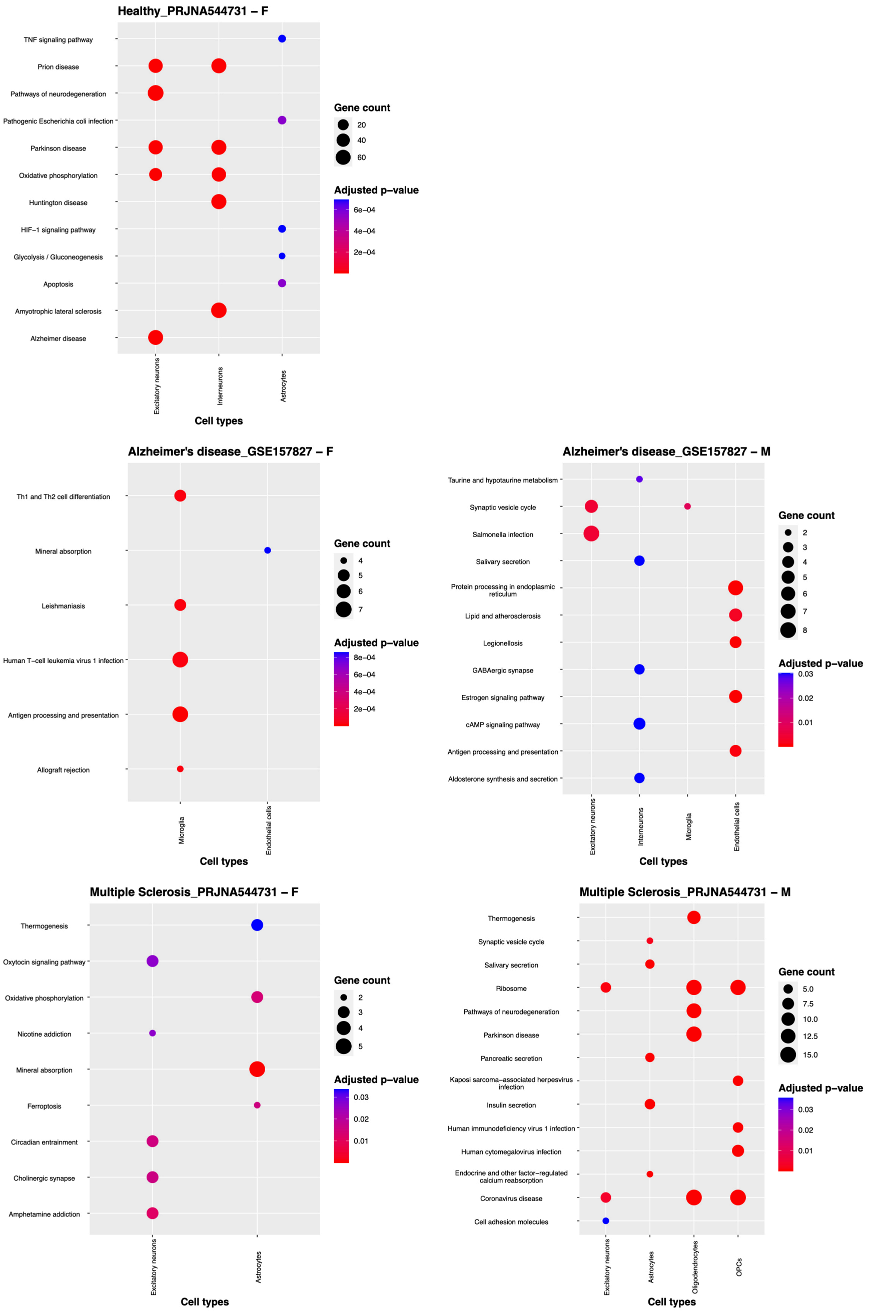
Healthy_PRJNA544731 – F
TNF signaling pathway
Prion disease
Pathways of neurodegeneration
Pathogenic Escherichia coli infection
Parkinson disease
Oxidative phosphorylation
Huntington disease
HIF−1 signaling pathway
Glycolysis / Gluconeogenesis
Apoptosis
Amyotrophic lateral sclerosis
Alzheimer disease
Excitatory neurons
Interneurons
Astrocytes
Cell types
Gene count
20
40
60
Adjusted p−value
6e−04
4e−04
2e−04
Alzheimer's disease_GSE157827 – F
Th1 and Th2 cell differentiation
Mineral absorption
Leishmaniasis
Human T−cell leukemia virus 1 infection
Antigen processing and presentation
Allograft rejection
Microglia
Endothelial cells
Cell types
Gene count
4
5
6
7
Adjusted p−value
8e−04
6e−04
4e−04
2e−04
Alzheimer's disease_GSE157827 – M
Taurine and hypotaurine metabolism
Synaptic vesicle cycle
Salmonella infection
Salivary secretion
Protein processing in endoplasmic reticulum
Lipid and atherosclerosis
Legionellosis
GABAergic synapse
Estrogen signaling pathway
cAMP signaling pathway
Antigen processing and presentation
Aldosterone synthesis and secretion
Excitatory neurons
Interneurons
Microglia
Endothelial cells
Cell types
Gene count
2
3
4
5
6
7
8
Adjusted p−value
0.03
0.02
0.01
Multiple Sclerosis_PRJNA544731 – F
Thermogenesis
Oxytocin signaling pathway
Oxidative phosphorylation
Nicotine addiction
Mineral absorption
Ferroptosis
Circadian entrainment
Cholinergic synapse
Amphetamine addiction
Excitatory neurons
Astrocytes
Cell types
Gene count
2
3
4
5
Adjusted p−value
0.03
0.02
0.01
Multiple Sclerosis_PRJNA544731 – M
Thermogenesis
Synaptic vesicle cycle
Salivary secretion
Ribosome
Pathways of neurodegeneration
Parkinson disease
Pancreatic secretion
Kaposi sarcoma−associated herpesvirus infection
Insulin secretion
Human immunodeficiency virus 1 infection
Human cytomegalovirus infection
Endocrine and other factor−regulated calcium reabsorption
Coronavirus disease
Cell adhesion molecules
Excitatory neurons
Astrocytes
Oligodendrocytes
OPCs
Cell types
Gene count
5.0
7.5
10.0
12.5
15.0
Adjusted p−value
0.03
0.02
0.01

Fig. S36: **Most Kyoto Encyclopedia of Genes and Genomes (KEGG) pathways were shared across cell types in each dataset-sex combination.** These plots show the KEGG pathways enrichment in each dataset, across cell types and for each sex. The dot size indicate show many genes were found to belong to each pathway, and the color is the adjusted p-value (Benjamini-Hochberg correction).

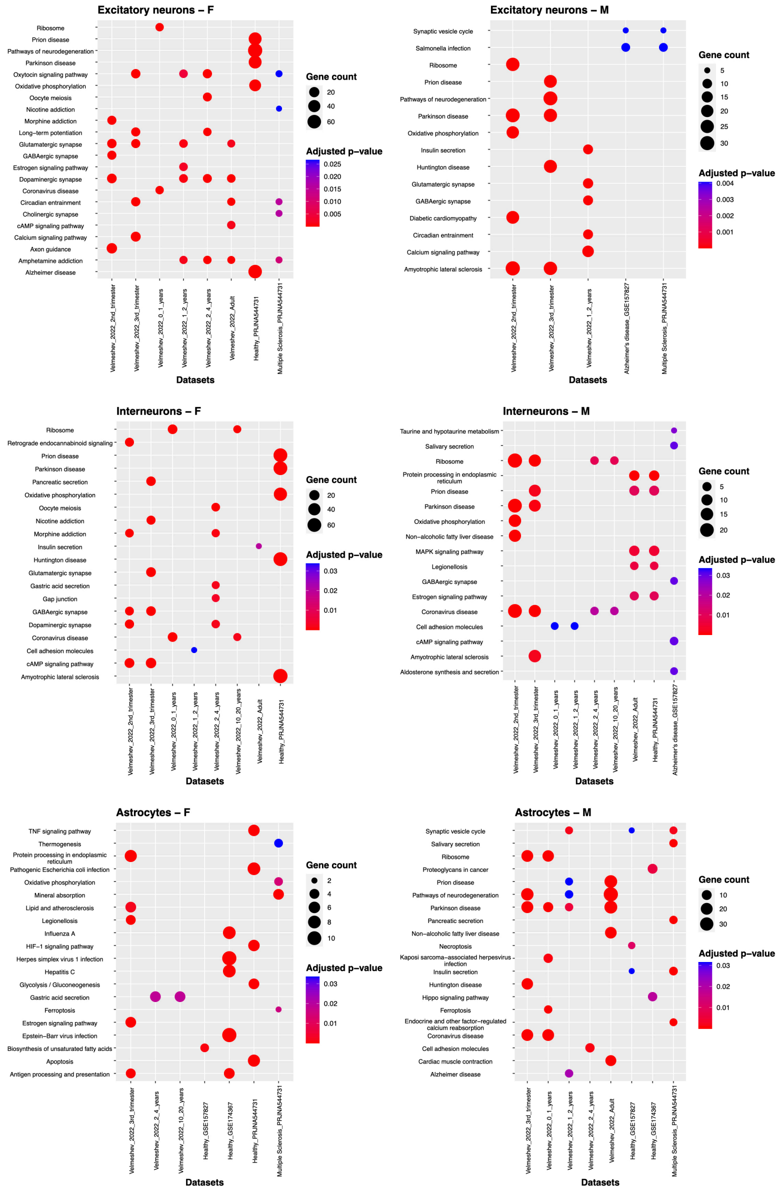

Fig. S37: **Most Kyoto Encyclopedia of Genes and Genomes (KEGG) pathways were shared across datasets in each cell type-sex combination.** These plots show the KEGG pathways enrichment in each cell type, across datasets and for each sex. The dot size indicate show many genes were found to belong to each pathway, and the color is the adjusted p-value (Benjamini-Hochberg correction).

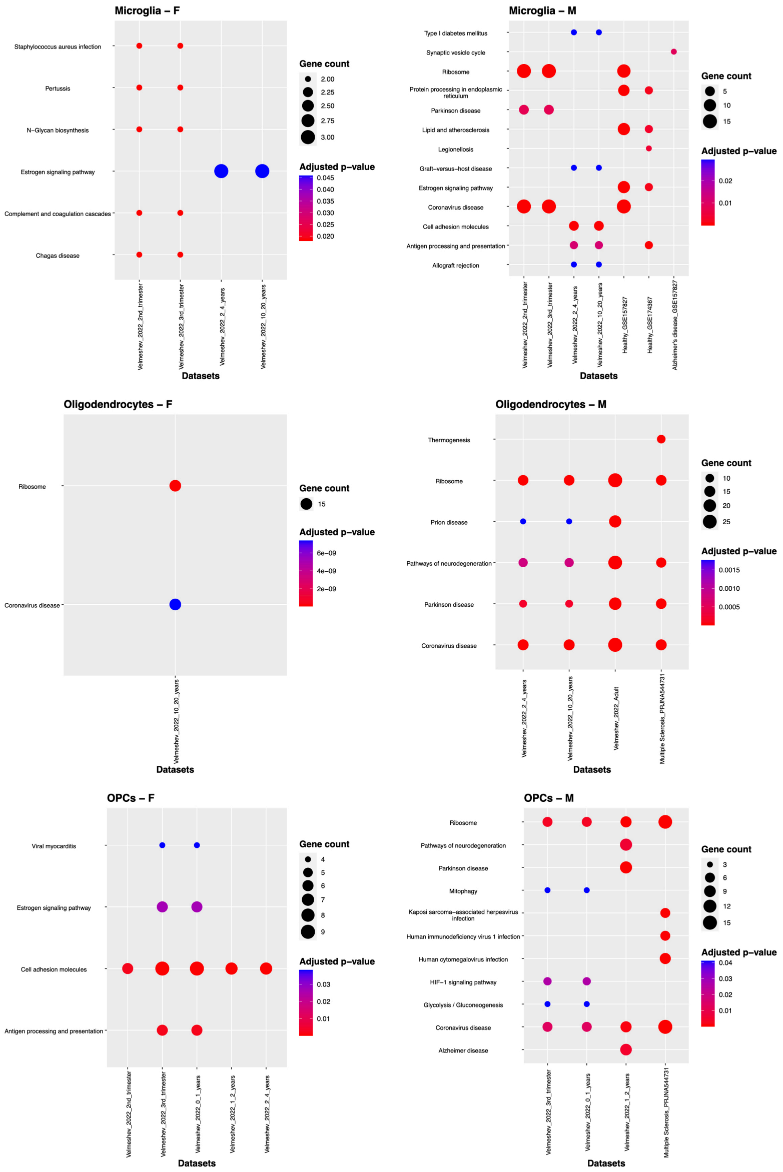

Fig. S38: **Most Kyoto Encyclopedia of Genes and Genomes (KEGG) pathways were shared across datasets in each cell type-sex combination.** These plots show the KEGG pathways enrichment in each cell type, across datasets and for each sex. The dot size indicate show many genes were found to belong to each pathway, and the color is the adjusted p-value (Benjamini-Hochberg correction).

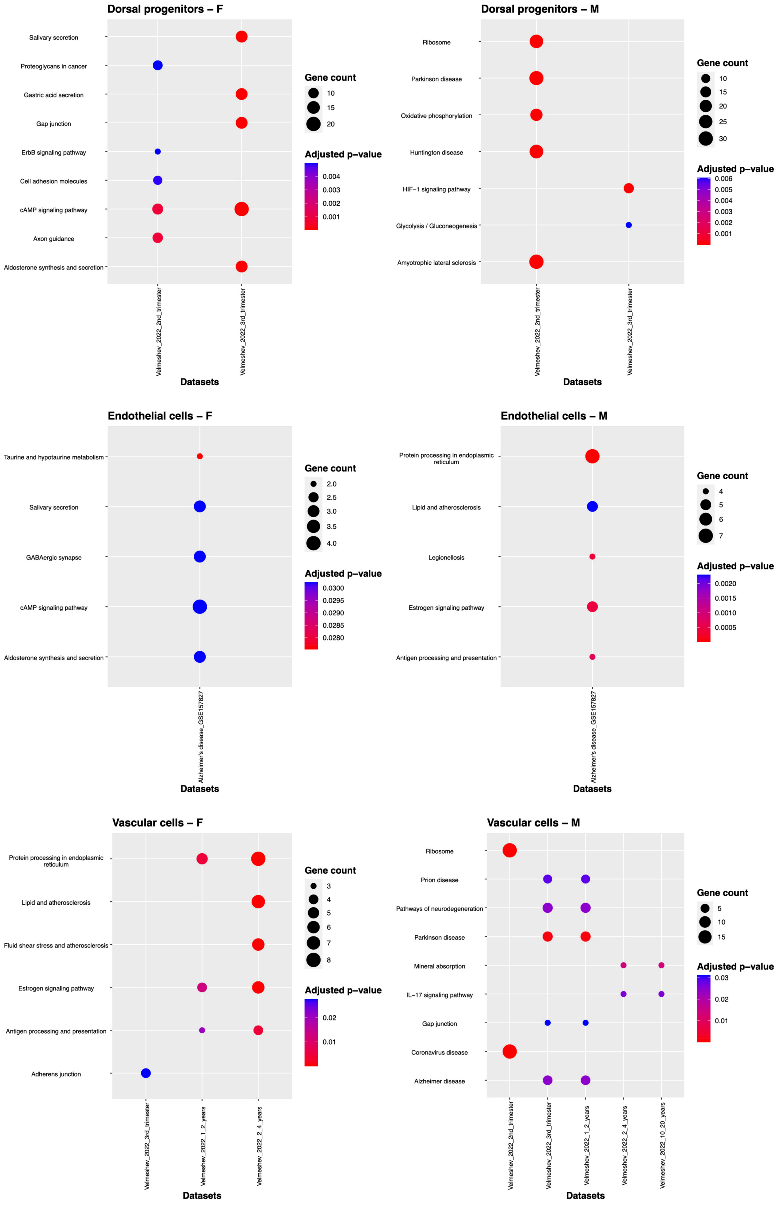

Fig. S39: **Most Kyoto Encyclopedia of Genes and Genomes (KEGG) pathways were shared across datasets in each cell type-sex combination.** These plots show the KEGG pathways enrichment in each cell type, across datasets and for each sex. The dot size indicate show many genes were found to belong to each pathway, and the color is the adjusted p-value (Benjamini-Hochberg correction).

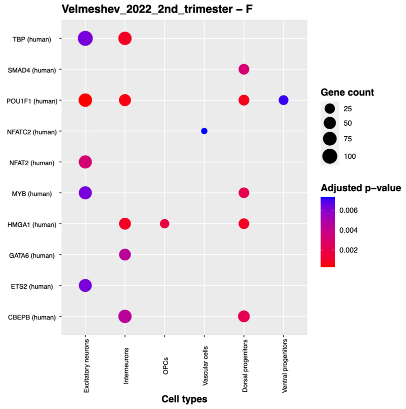
Velmeshev_2022_2nd_trimester – F
TBP (human)
SMAD4 (human)
POU1F1 (human)
NFATC2 (human)
NFAT2 (human)
MYB (human)
HMGA1 (human)
GATA6 (human)
ETS2 (human)
CBEPB (human)
Excitatory neurons
Interneurons
OPCs
Vascular cells
Dorsal progenitors
Ventral progenitors
Cell types
Gene count
25
50
75
100
Adjusted p–value
0.006
0.004
0.002

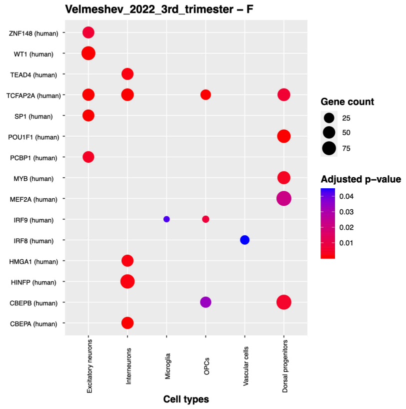
Velmeshev_2022_3rd_trimester – F
ZNF148 (human)
WT1 (human)
TEAD4 (human)
TCFAP2A (human)
SP1 (human)
POU1F1 (human)
PCBP1 (human)
MYB (human)
MEF2A (human)
IRF9 (human)
IRF8 (human)
HMGA1 (human)
HINFP (human)
CBEPB (human)
CBEPA (human)
Excitatory neurons
Interneurons
Microglia
OPCs
Vascular cells
Dorsal progenitors
Cell types
Gene count
25
50
75
Adjusted p–value
0.04
0.03
0.02
0.01

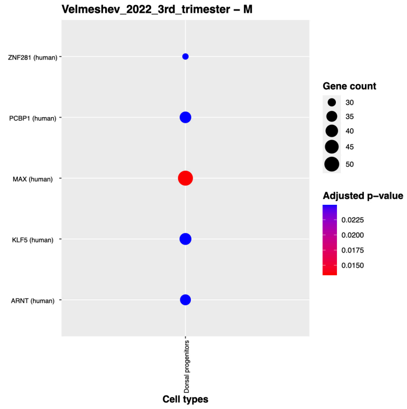
Velmeshev_2022_3rd_trimester – M
ZNF281 (human)
PCBP1 (human)
MAX (human)
KLF5 (human)
ARNT (human)
Dorsal progenitors
Cell types
Gene count
30
35
40
45
50
Adjusted p–value
0.0225
0.0200
0.0175
0.0150

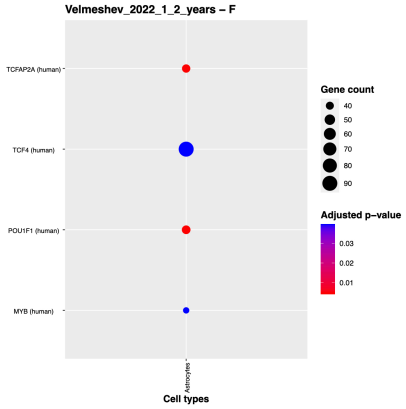
Velmeshev_2022_1_2_years – F
TCFAP2A (human)
TCF4 (human)
POU1F1 (human)
MYB (human)
Astrocytes
Cell types
Gene count
40
50
60
70
80
90
Adjusted p–value
0.03
0.02
0.01

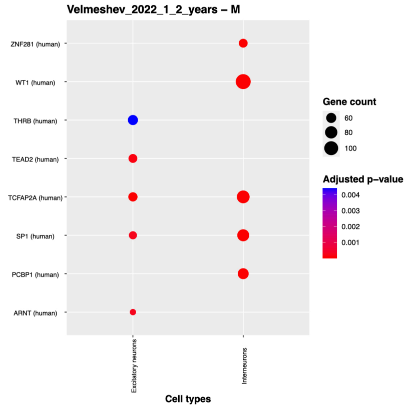
Velmeshev_2022_1_2_years – M
ZNF281 (human)
WT1 (human)
THRB (human)
TEAD2 (human)
TCFAP2A (human)
SP1 (human)
PCBP1 (human)
ARNT (human)
Excitatory neurons
Interneurons
Cell types
Gene count
60
80
100
Adjusted p–value
0.004
0.003
0.002
0.001

Fig. S40: **TRANSFAC and JASPAR PWMs binding sites enrichment showed some overlap across cell types.** These plots show the TRANSFAC and JASPAR PWMs binding sites enrichment in each dataset, across cell types and for each sex. The dot size indicate show many genes were found to belong to each pathway, and the color is the adjusted p-value (Benjamini-Hochberg correction). TCFAP2A was the only TF binding site to be enriched in at least half of the datasets in females.

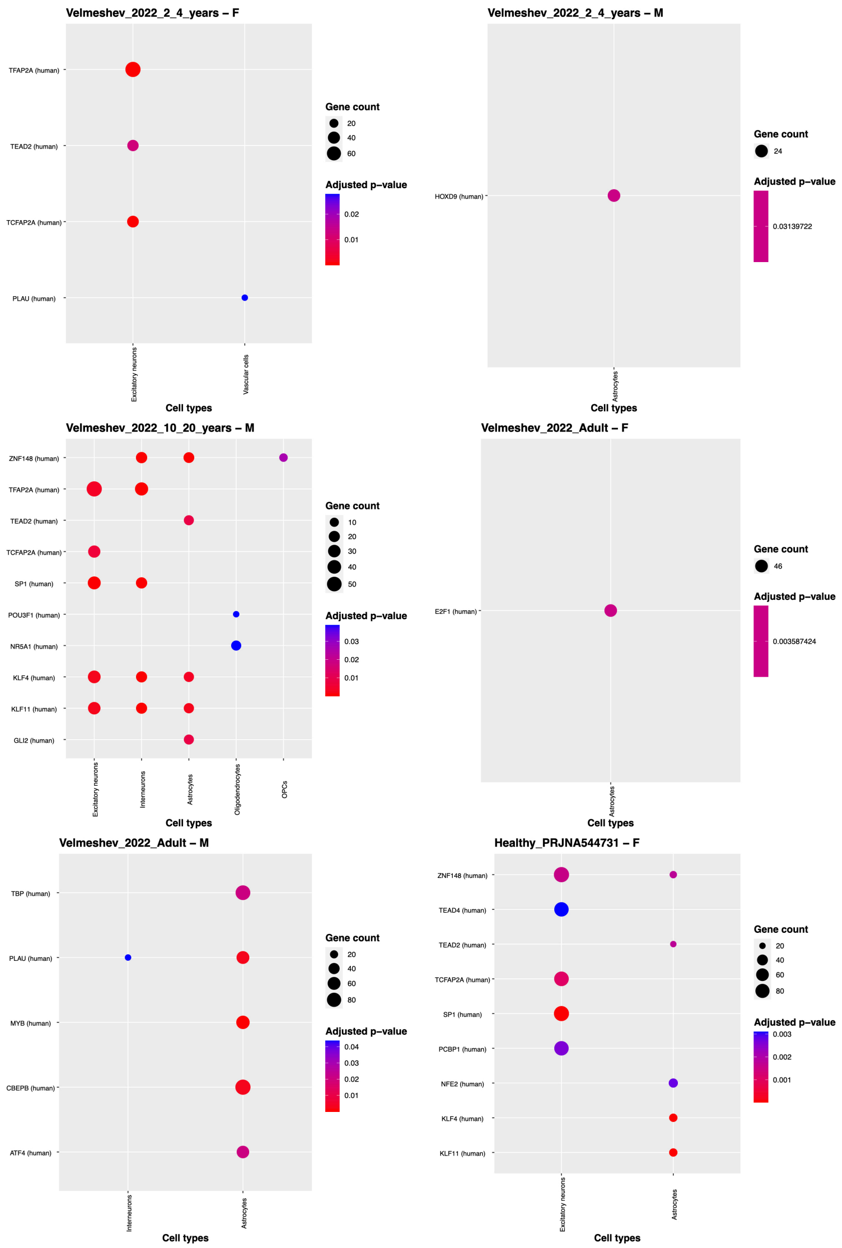

Fig. S41: **TRANSFAC and JASPAR PWMs binding sites enrichment showed some overlap across cell types.** These plots show the TRANSFAC and JASPAR PWMs binding sites enrichment in each dataset, across cell types and for each sex. The dot size indicate show many genes were found to belong to each pathway, and the color is the adjusted p-value (Benjamini-Hochberg correction). TCFAP2A was the only TF binding site to be enriched in at least half of the datasets in females.

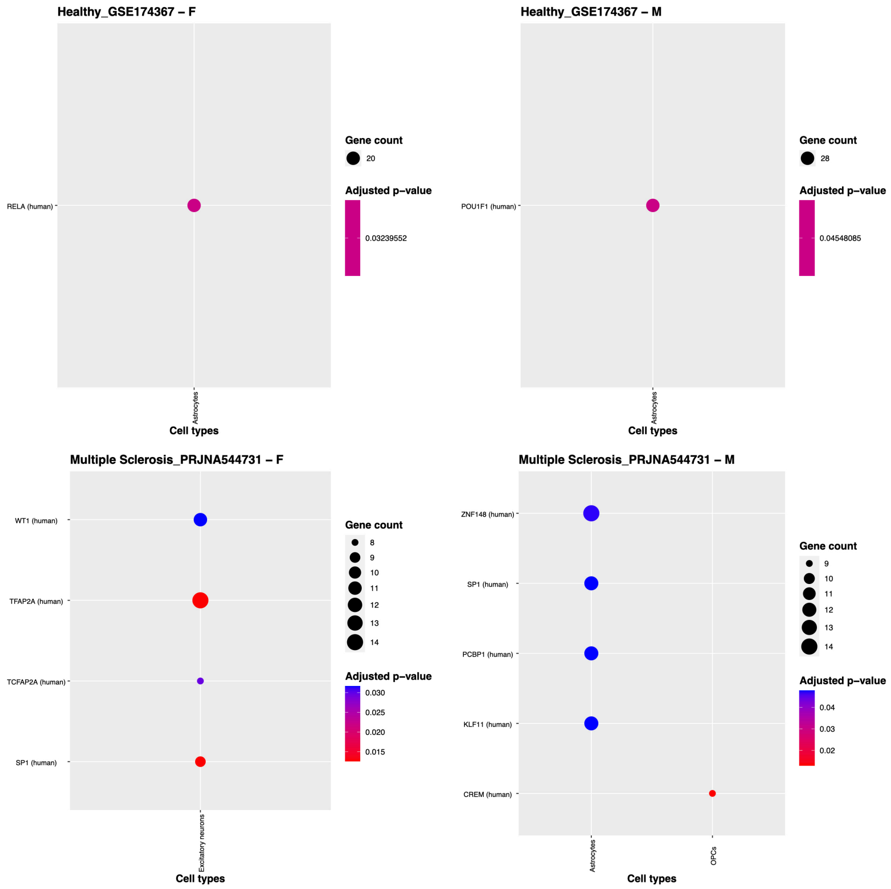

Fig. S42: **TRANSFAC and JASPAR PWMs binding sites enrichment showed some overlap across cell types.** These plots show the TRANSFAC and JASPAR PWMs binding sites enrichment in each dataset, across cell types and for each sex. The dot size indicate show many genes were found to belong to each pathway, and the color is the adjusted p-value (Benjamini-Hochberg correction). TCFAP2A was the only TF binding site to be enriched in at least half of the datasets in females.

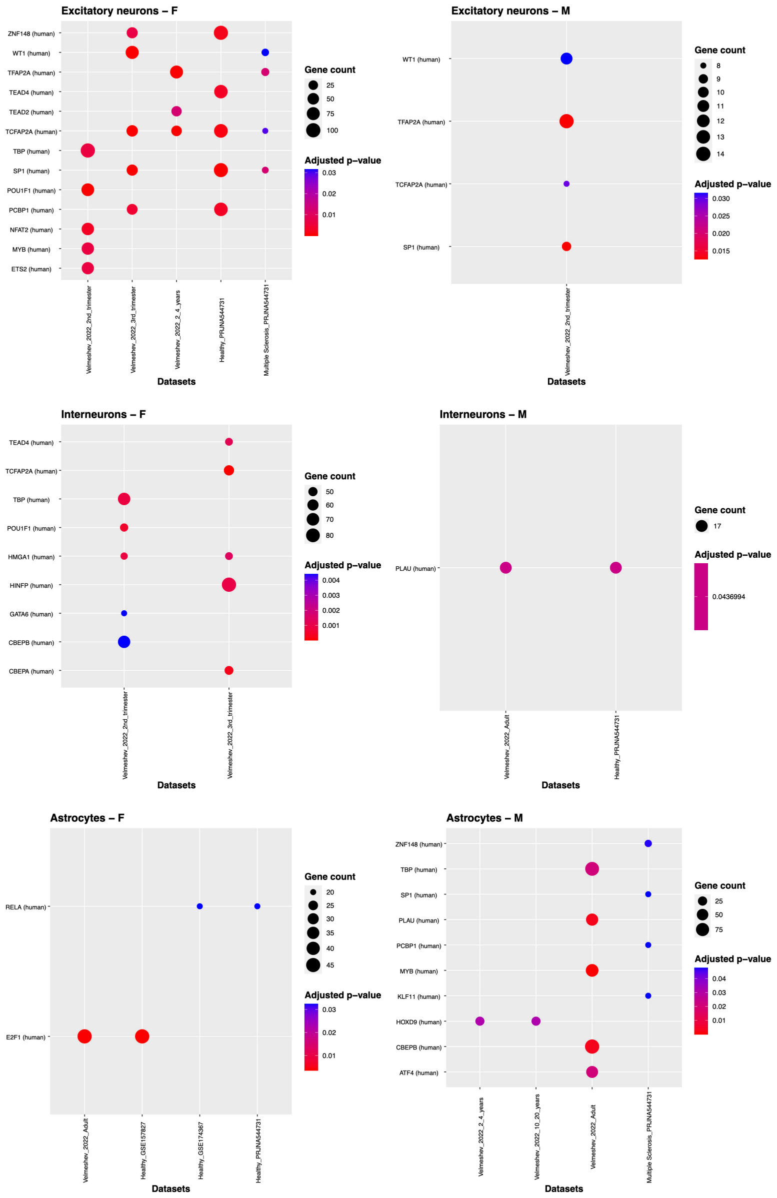

Fig. S43: **TRANSFAC and JASPAR PWMs binding sites enrichment showed some overlap across datasets.** These plots show the TRANSFAC and JASPAR PWMs binding sites enrichment in each cell type, across datasets and for each sex. The dot size indicate show many genes were found to belong to each pathway, and the color is the adjusted p-value (Benjamini-Hochberg correction). TCFAP2A and POU1F1 were the only TF binding sites to be enriched in at least half of the datasets in females.

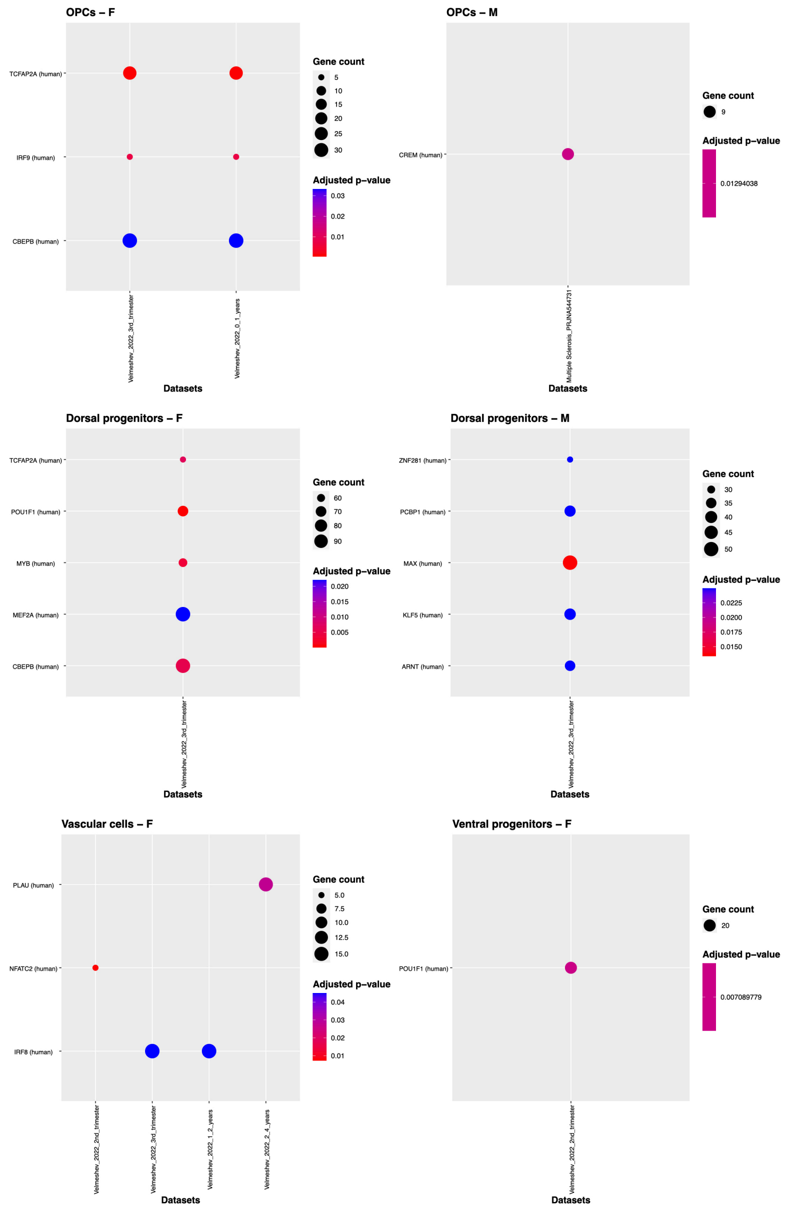

Fig. S44: **TRANSFAC and JASPAR PWMs binding sites enrichment showed some overlap across datasets.** These plots show the TRANSFAC and JASPAR PWMs binding sites enrichment in each cell type, across datasets and for each sex. The dot size indicate show many genes were found to belong to each pathway, and the color is the adjusted p-value (Benjamini-Hochberg correction). TCFAP2A and POU1F1 were the only TF binding sites to be enriched in at least half of the datasets in females.

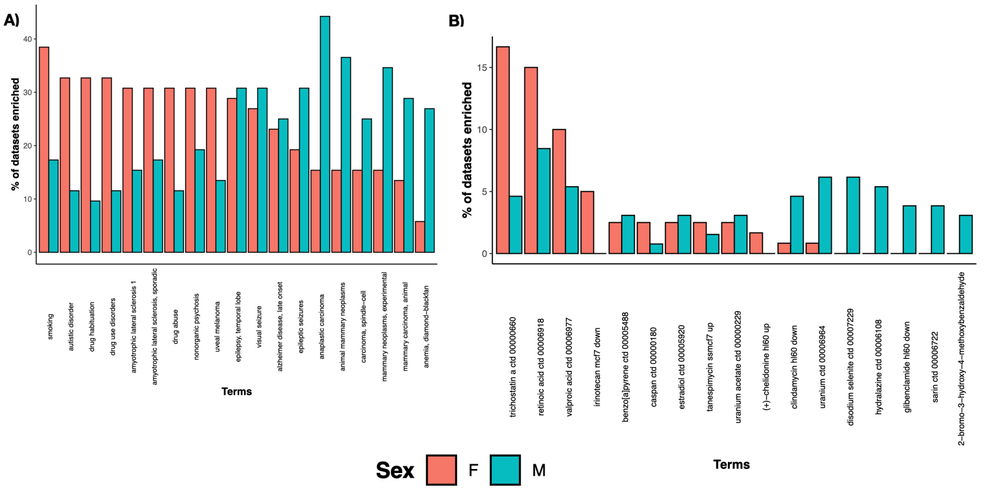

Fig. S45: **SG-biased genes show biased enrichment for diseases (including autism) and drugs.** A) The 10 most frequent disease-related terms in each sex, regardless of dataset, cell type and database used, is represented in this bar plot, shown as percentage of how many datasets were enriched for each term. B) This bar plot shows the 10 most frequent drug-related terms in each sex, regardless of dataset and cell type. Females show higher percentages of datasets sharing the same drug terms, although most of the same terms could be found in males. On the other hand, male-biased drug terms seemed to show a specificity, with some of them found solely in males.

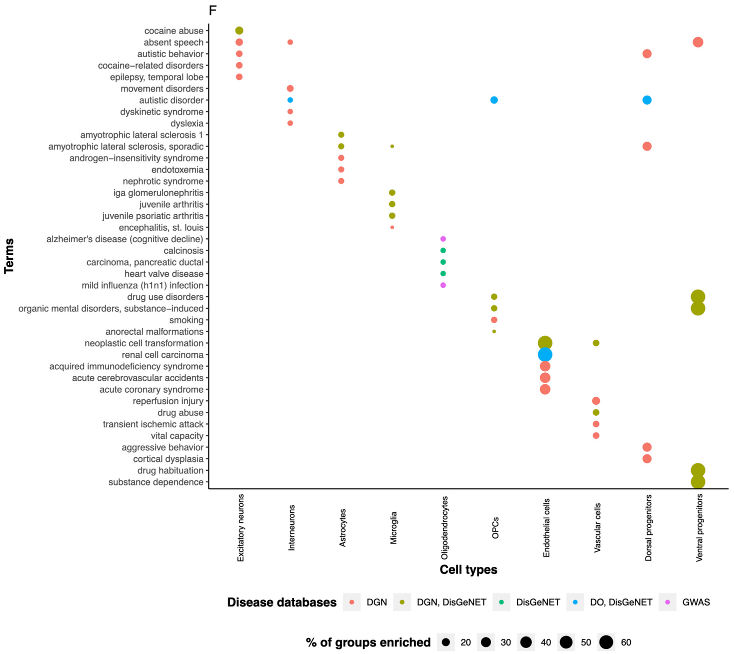
F
Terms
cocaine abuse
absent speech
autistic behavior
cocaine−related disorders
epilepsy, temporal lobe
movement disorders
autistic disorder
dyskinetic syndrome
dyslexia
amyotrophic lateral sclerosis 1
amyotrophic lateral sclerosis, sporadic
androgen−insensitivity syndrome
endotoxemia
nephrotic syndrome
iga glomerulonephritis
juvenile arthritis
juvenile psoriatic arthritis
encephalitis, st. louis
alzheimer's disease (cognitive decline)
calcinosis
carcinoma, pancreatic ductal
heart valve disease
mild influenza (h1n1) infection
drug use disorders
organic mental disorders, substance−induced
smoking
anorectal malformations
neoplastic cell transformation
renal cell carcinoma
acquired immunodeficiency syndrome
acute cerebrovascular accidents
acute coronary syndrome
reperfusion injury
drug abuse
transient ischemic attack
vital capacity
aggressive behavior
cortical dysplasia
drug habituation
substance dependence
Excitatory neurons
Interneurons
Astrocytes
Microglia
Oligodendrocytes
OPCs
Endothelial cells
Vascular cells
Dorsal progenitors
Ventral progenitors
Cell types
Disease databases
DGN
DGN, DisGeNET
DisGeNET
DO, DisGeNET
GWAS
% of groups enriched
20
30
40
50
60

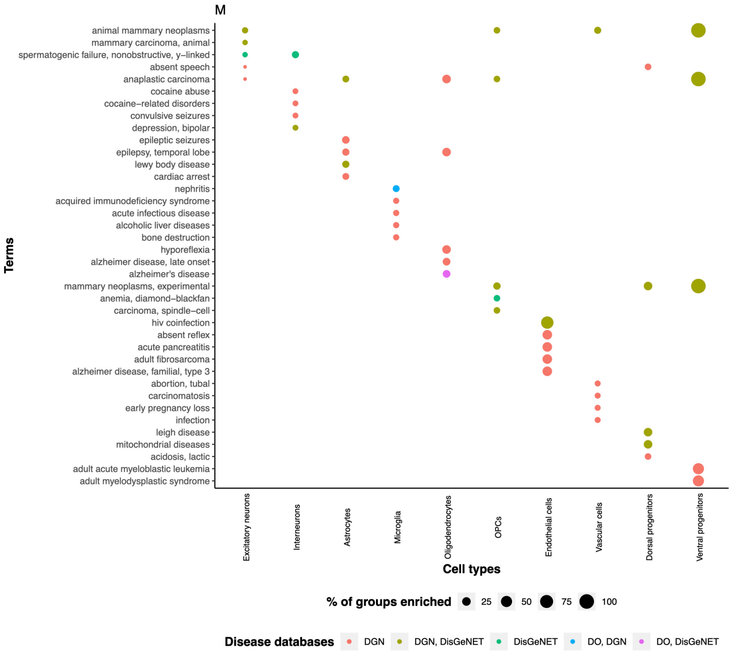
M
Terms
animal mammary neoplasms
mammary carcinoma, animal
spermatogenic failure, nonobstructive, y−linked
absent speech
anaplastic carcinoma
cocaine abuse
cocaine−related disorders
convulsive seizures
depression, bipolar
epileptic seizures
epilepsy, temporal lobe
lewy body disease
cardiac arrest
nephritis
acquired immunodeficiency syndrome
acute infectious disease
alcoholic liver diseases
bone destruction
hyporeflexia
alzheimer disease, late onset
alzheimer's disease
mammary neoplasms, experimental
anemia, diamond−blackfan
carcinoma, spindle−cell
hiv coinfection
absent reflex
acute pancreatitis
adult fibrosarcoma
alzheimer disease, familial, type 3
abortion, tubal
carcinomatosis
early pregnancy loss
infection
leigh disease
mitochondrial diseases
acidosis, lactic
adult acute myeloblastic leukemia
adult myelodysplastic syndrome
Excitatory neurons
Interneurons
Astrocytes
Microglia
Oligodendrocytes
OPCs
Endothelial cells
Vascular cells
Dorsal progenitors
Ventral progenitors
Cell types
% of groups enriched
25
50
75
100
Disease databases
DGN
DGN, DisGeNET
DisGeNET
DO, DGN
DO, DisGeNET

Fig. S46: **The disease-related term were mostly sex-biased, and found in more than one disease database.** These dot plots show the top 5 disease-related enriched terms, divided by sex, cell type and database which showed the enrichment in the terms. The dot size indicates how many datasets presented each term, and the color indicates in which combination of databases was the term found.

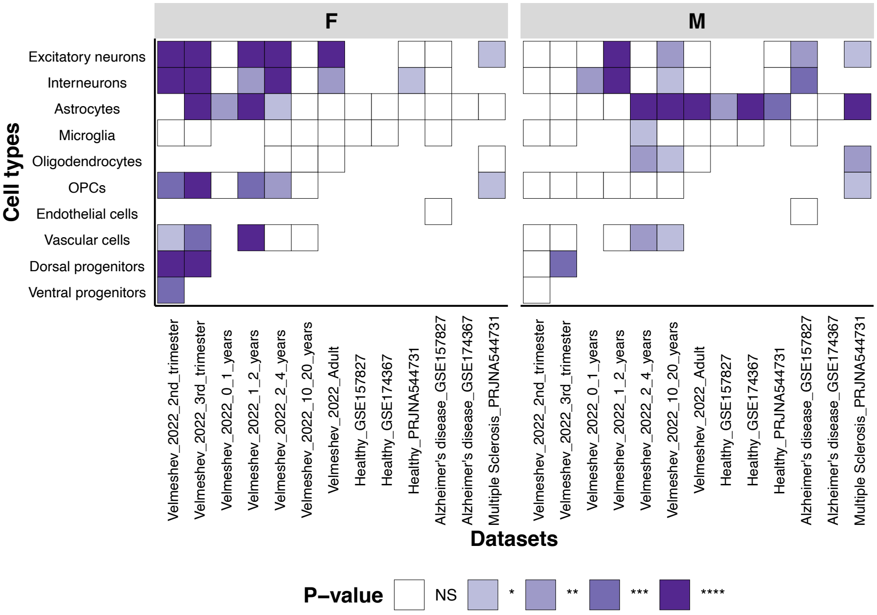

Fig. S47: **Female-biased genes showed enrichment in the early age groups for autism-related genes.** This heatmap shows the hyper-geometric enrichment of SFARI genes in the SG-biased DEGs. Most of the SFARI genes enrichment was found in females, an in earlier age datasets. **Legend:** NS: not significant; *: p<0.05; **: p<0.01; ***: p<0.001; ****: p<0.0001.

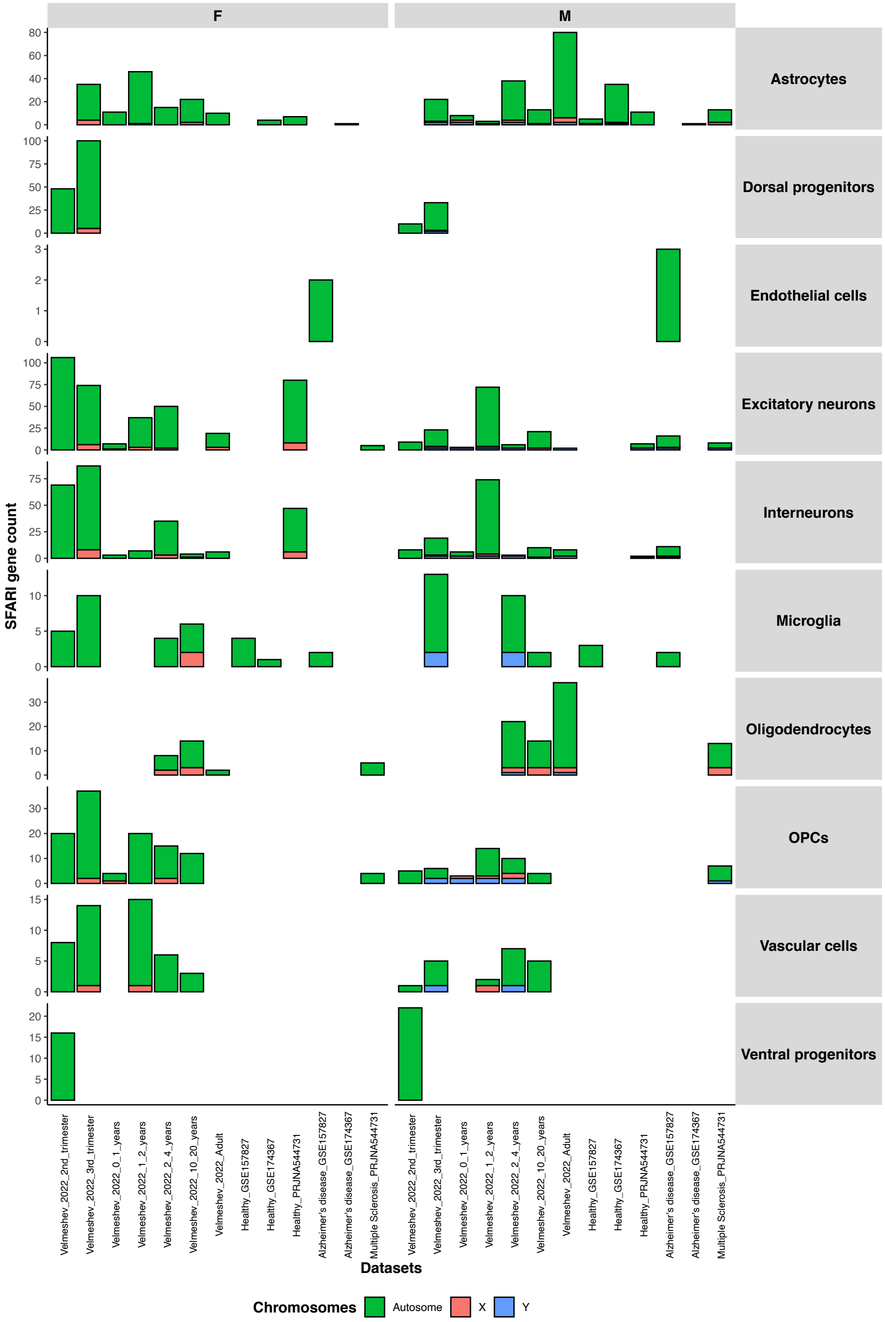

F
M
Astrocytes
Dorsal progenitors
Endothelial cells
Excitatory neurons
Interneurons
Microglia
Oligodendrocytes
OPCs
Vascular cells
Ventral progenitors
SFARI gene count
Velmeshev_2022_2nd_trimester
Velmeshev_2022_3rd_trimester
Velmeshev_2022_0_1_years
Velmeshev_2022_1_2_years
Velmeshev_2022_2_4_years
Velmeshev_2022_10_20_years
Velmeshev_2022_Adult
Healthy_GSE157827
Healthy_GSE174367
Healthy_PRJNA544731
Alzheimer's disease_GSE157827
Alzheimer's disease_GSE174367
Multiple Sclerosis_PRJNA544731
Datasets
Chromosomes
Autosome
X
Y

Fig. S48: **Most SG-biased DEG lists contained genes related to autism, based on the SFARI reference database, and such genes were mostly autosomal.** These bar plots show the SFARI absolute gene count found in each SG-biased DEG lists, divided by sex, cell type and dataset. Overall, the gene counts were the highest in female-biased genes in the fetal datasets, while the male-biased genes showed higher counts in later datasets. Additionally, most of the SFARI genes were autosomal, and only few SFARI genes belonged to the X or Y chromosomes.

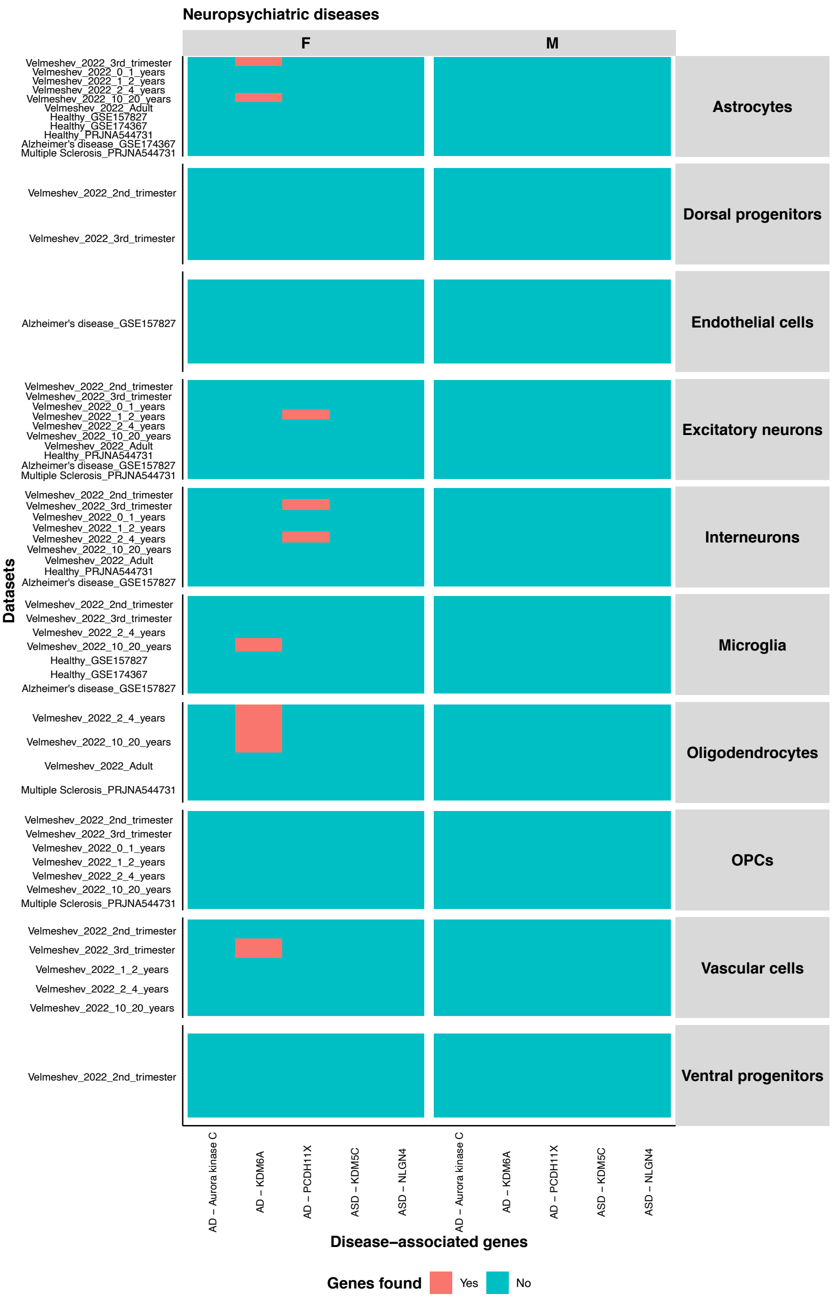
Neuropsychiatric diseases
F
M
Datasets
Astrocytes
Velmeshev_2022_3rd_trimester
Velmeshev_2022_0_1_years
Velmeshev_2022_1_2_years
Velmeshev_2022_2_4_years
Velmeshev_2022_10_20_years
Velmeshev_2022_Adult
Healthy_GSE157827
Healthy_GSE174367
Healthy_PRJNA544731
Alzheimer's disease_GSE174367
Multiple Sclerosis_PRJNA544731
Dorsal progenitors
Velmeshev_2022_2nd_trimester
Velmeshev_2022_3rd_trimester
Endothelial cells
Alzheimer's disease_GSE157827
Excitatory neurons
Velmeshev_2022_2nd_trimester
Velmeshev_2022_3rd_trimester
Velmeshev_2022_0_1_years
Velmeshev_2022_1_2_years
Velmeshev_2022_2_4_years
Velmeshev_2022_10_20_years
Velmeshev_2022_Adult
Healthy_PRJNA544731
Alzheimer's disease_GSE157827
Multiple Sclerosis_PRJNA544731
Interneurons
Velmeshev_2022_2nd_trimester
Velmeshev_2022_3rd_trimester
Velmeshev_2022_0_1_years
Velmeshev_2022_1_2_years
Velmeshev_2022_2_4_years
Velmeshev_2022_10_20_years
Velmeshev_2022_Adult
Healthy_PRJNA544731
Alzheimer's disease_GSE157827
Microglia
Velmeshev_2022_2nd_trimester
Velmeshev_2022_3rd_trimester
Velmeshev_2022_2_4_years
Velmeshev_2022_10_20_years
Healthy_GSE157827
Healthy_GSE174367
Alzheimer's disease_GSE157827
Oligodendrocytes
Velmeshev_2022_2_4_years
Velmeshev_2022_10_20_years
Velmeshev_2022_Adult
Multiple Sclerosis_PRJNA544731
OPCs
Velmeshev_2022_2nd_trimester
Velmeshev_2022_3rd_trimester
Velmeshev_2022_0_1_years
Velmeshev_2022_1_2_years
Velmeshev_2022_2_4_years
Velmeshev_2022_10_20_years
Multiple Sclerosis_PRJNA544731
Vascular cells
Velmeshev_2022_2nd_trimester
Velmeshev_2022_3rd_trimester
Velmeshev_2022_1_2_years
Velmeshev_2022_2_4_years
Velmeshev_2022_10_20_years
Ventral progenitors
Velmeshev_2022_2nd_trimester
AD – Aurora kinase C
AD – KDM6A
AD – PCDH11X
ASD – KDM5C
ASD – NLGN4
AD – Aurora kinase C
AD – KDM6A
AD – PCDH11X
ASD – KDM5C
ASD – NLGN4
Disease–associated genes
Genes found
Yes
No

Fig. S49: **Neuropsychiatric disease-related genes were mainly found in female-biased genes.** This heatmap shows the presence of neuropsychiatric disease-associated genes (DAGs) among the SG-biased genes. Only two genes were found among the DAGs, KDM6A and PCDH11X, both related to AD. Interestingly, both were found mainly from fetal to young adulthood datasets, but not in the disease datasets. **Abbreviations:** AD: Alzheimer's disease; ASD: autism spectrum disorder.

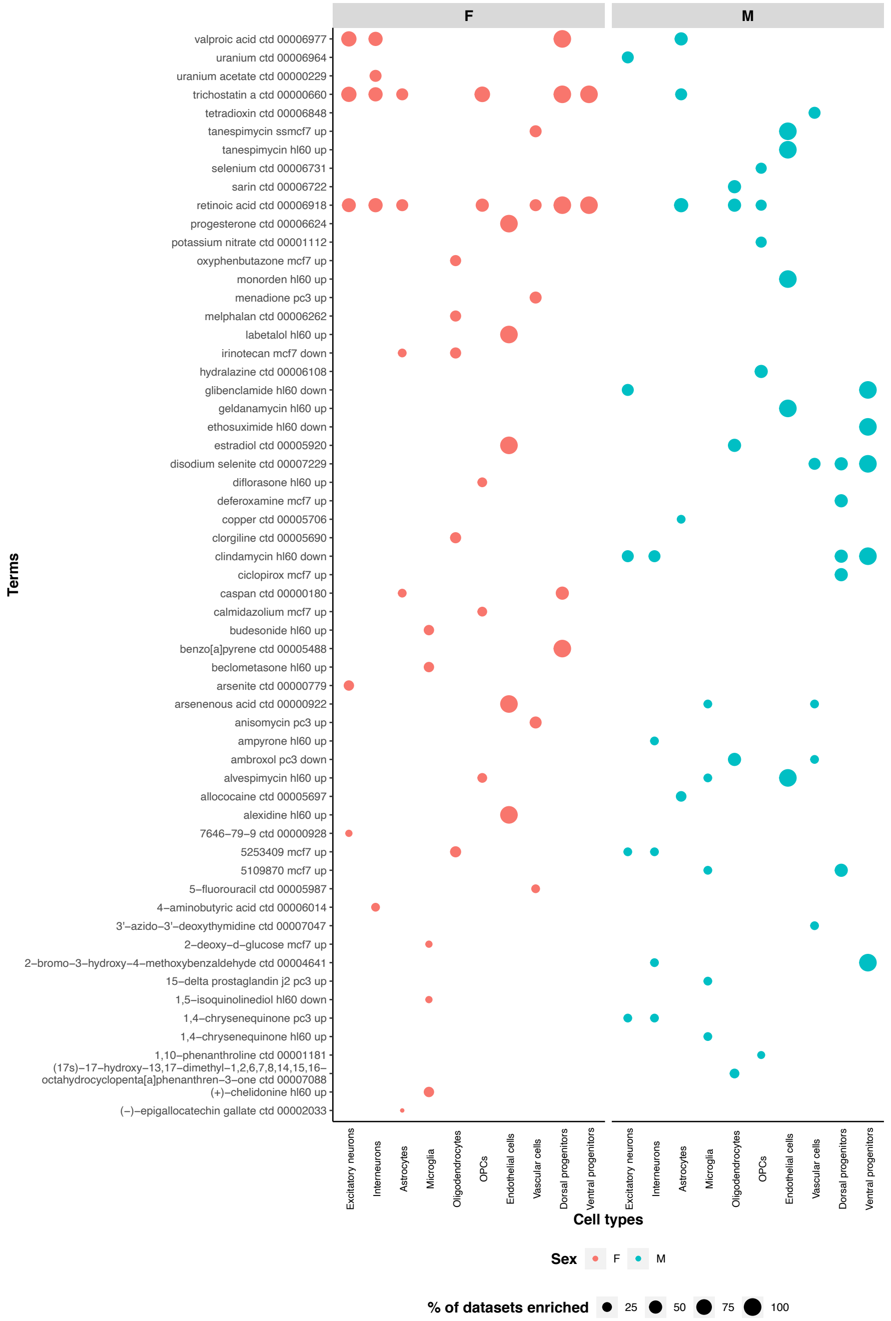
F
M
valproic acid ctd 00006977
uranium ctd 00006964
uranium acetate ctd 00000229
trichostatin a ctd 00000660
tetradioxin ctd 00006848
tanespimycin ssmcf7 up
tanespimycin hl60 up
selenium ctd 00006731
sarin ctd 00006722
retinoic acid ctd 00006918
progesterone ctd 00006624
potassium nitrate ctd 00001112
oxyphenbutazone mcf7 up
monorden hl60 up
menadione pc3 up
melphalan ctd 00006262
labetalol hl60 up
irinotecan mcf7 down
hydralazine ctd 00006108
glibenclamide hl60 down
geldanamycin hl60 up
ethosuximide hl60 down
estradiol ctd 00005920
disodium selenite ctd 00007229
diflorasone hl60 up
deferoxamine mcf7 up
copper ctd 00005706
clorgiline ctd 00005690
clindamycin hl60 down
ciclopirox mcf7 up
caspan ctd 00000180
calmidazolium mcf7 up
budesonide hl60 up
benzo[a]pyrene ctd 00005488
beclometasone hl60 up
arsenite ctd 00000779
arsenous acid ctd 00000922
anisomycin pc3 up
ampyrone hl60 up
ambroxol pc3 down
alvespimycin hl60 up
allococaine ctd 00005697
alexidine hl60 up
7646−79−9 ctd 00000928
5253409 mcf7 up
5109870 mcf7 up
5−fluorouracil ctd 00005987
4−aminobutyric acid ctd 00006014
3'−azido−3'−deoxythymidine ctd 00007047
2−deoxy−d−glucose mcf7 up
2−bromo−3−hydroxy−4−methoxybenzaldehyde ctd 00004641
15−delta prostaglandin j2 pc3 up
1,5−isoquinolinediol hl60 down
1,4−chrysenequinone pc3 up
1,4−chrysenequinone hl60 up
1,10−phenanthroline ctd 00001181
(17s)−17−hydroxy−13,17−dimethyl−1,2,6,7,8,14,15,16−octahydrocyclopenta[a]phenanthren−3−one ctd 00007088
(+)−chelidonine hl60 up
(−)−epigallocatechin gallate ctd 00002033
Terms
Excitatory neurons
Interneurons
Astrocytes
Microglia
Oligodendrocytes
OPCs
Endothelial cells
Vascular cells
Dorsal progenitors
Ventral progenitors
Cell types
Sex
F
M
% of datasets enriched
25
50
75
100

Fig. S50: **Most enriched drug terms were sex-specific, with some exceptions.** These dot plots show the top 5 enriched drugs, divided by sex and cell type. The dot size indicates how many datasets were enriched for each term, and the color the sex where the term was found to be enriched in.

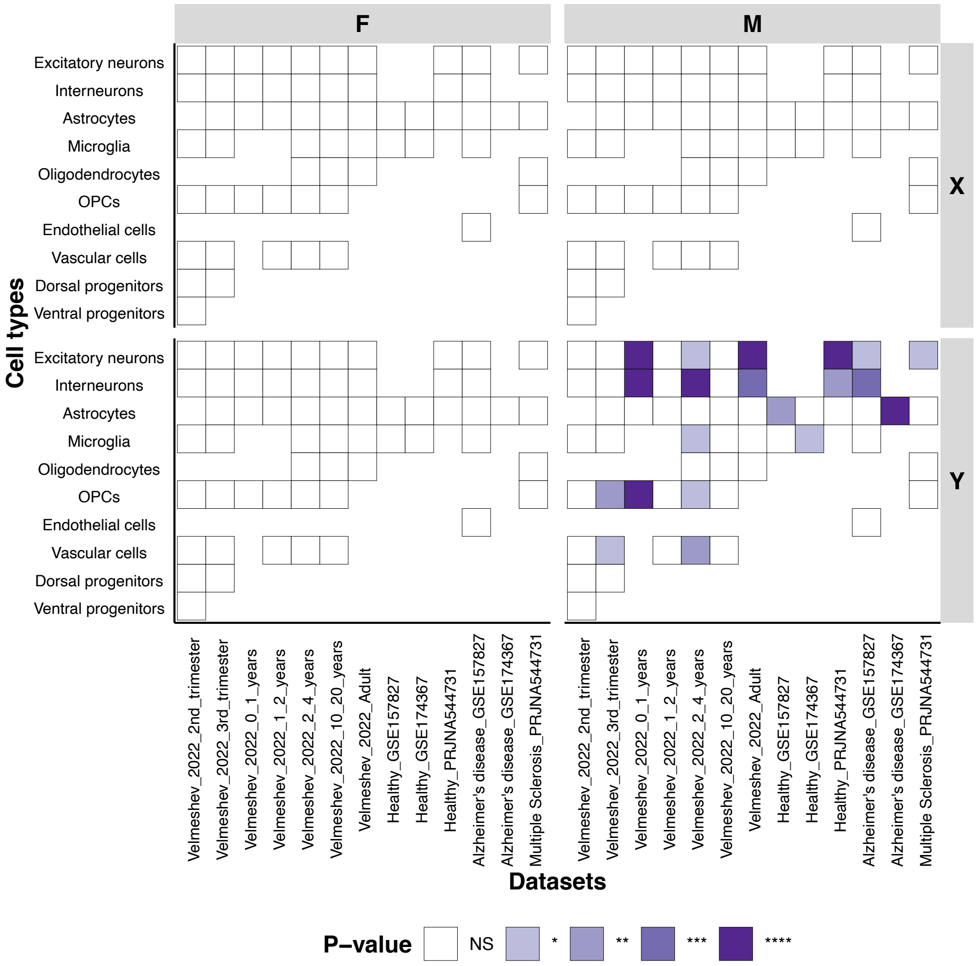

Fig. S51: **There was little Y chromosome enrichment in the male-biased genes, and no enrichment for X in female-biased genes.** This heatmap represents the significant hyper-geometric enrichment of sex chromosomes in the SG-biased genes. Grey cells represent cell types not analyzed in the datasets. The only enrichment we could find was for Y in males, as expected, although we found enrichment specifically in neuronal and glial populations. **Legend:** NS: not significant; *: $p<0.05$; **: $p<0.01$; ***: $p<0.001$; ****: $p<0.0001$.

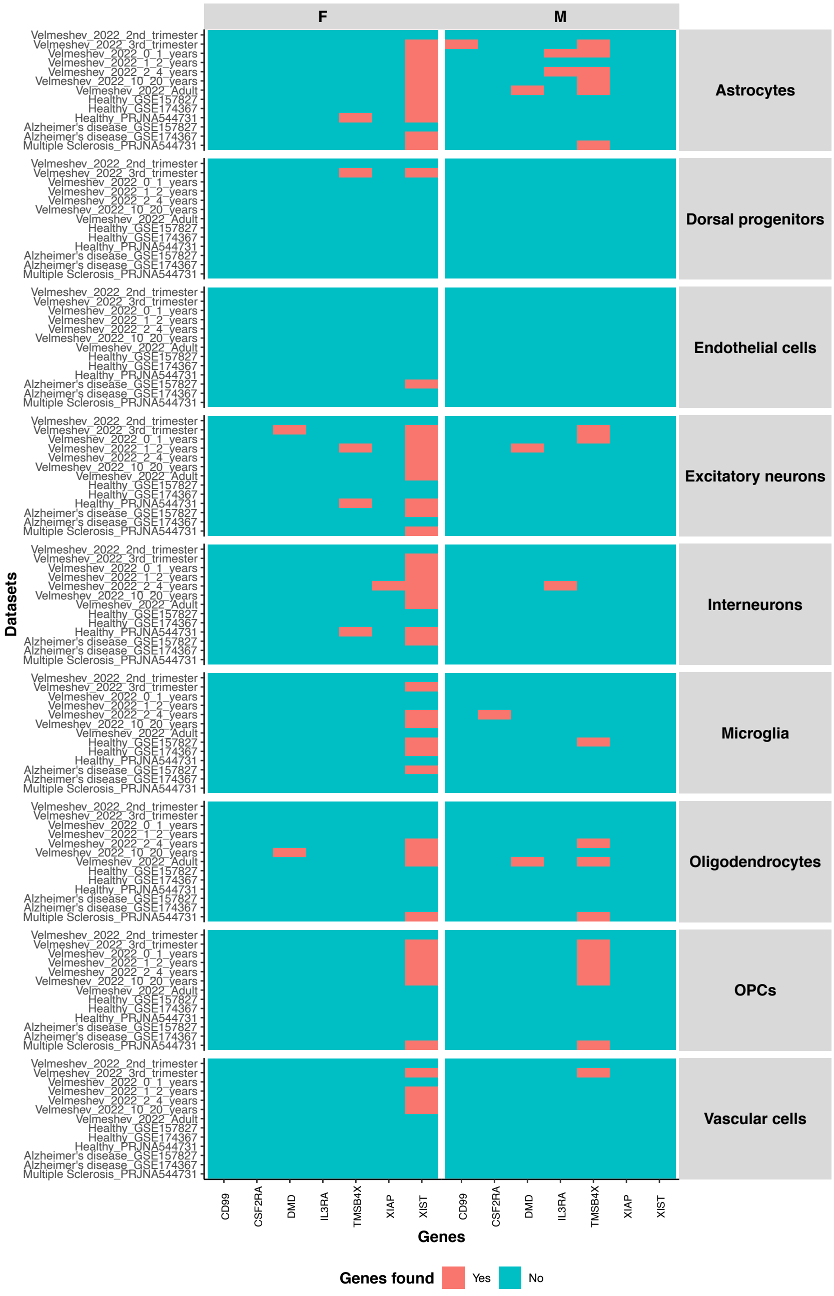
F
M
Astrocytes
Dorsal progenitors
Endothelial cells
Excitatory neurons
Interneurons
Microglia
Oligodendrocytes
OPCs
Vascular cells
Datasets
Velmeshev_2022_2nd_trimester
Velmeshev_2022_3rd_trimester
Velmeshev_2022_0_1_years
Velmeshev_2022_1_2_years
Velmeshev_2022_2_4_years
Velmeshev_2022_10_20_years
Velmeshev_2022_Adult
Healthy_GSE157827
Healthy_GSE174367
Healthy_PRJNA544731
Alzheimer's disease_GSE157827
Alzheimer's disease_GSE174367
Multiple Sclerosis_PRJNA544731
CD99
CSF2RA
DMD
IL3RA
TMSB4X
XIAP
XIST
Genes
Genes found
Yes
No

Fig. S52: **XCI genes are mostly not found in either females or males, with the exception of XIST and TMSB4X.** The heatmap show the presence of genes X-inactivation in SG-biased genes lists from each cell type and dataset. As expected, XIST is solely found in the female-biased genes. However, TMSB4X, which we expected to behave as XIST, was instead mostly found in male- and not female-biased genes.

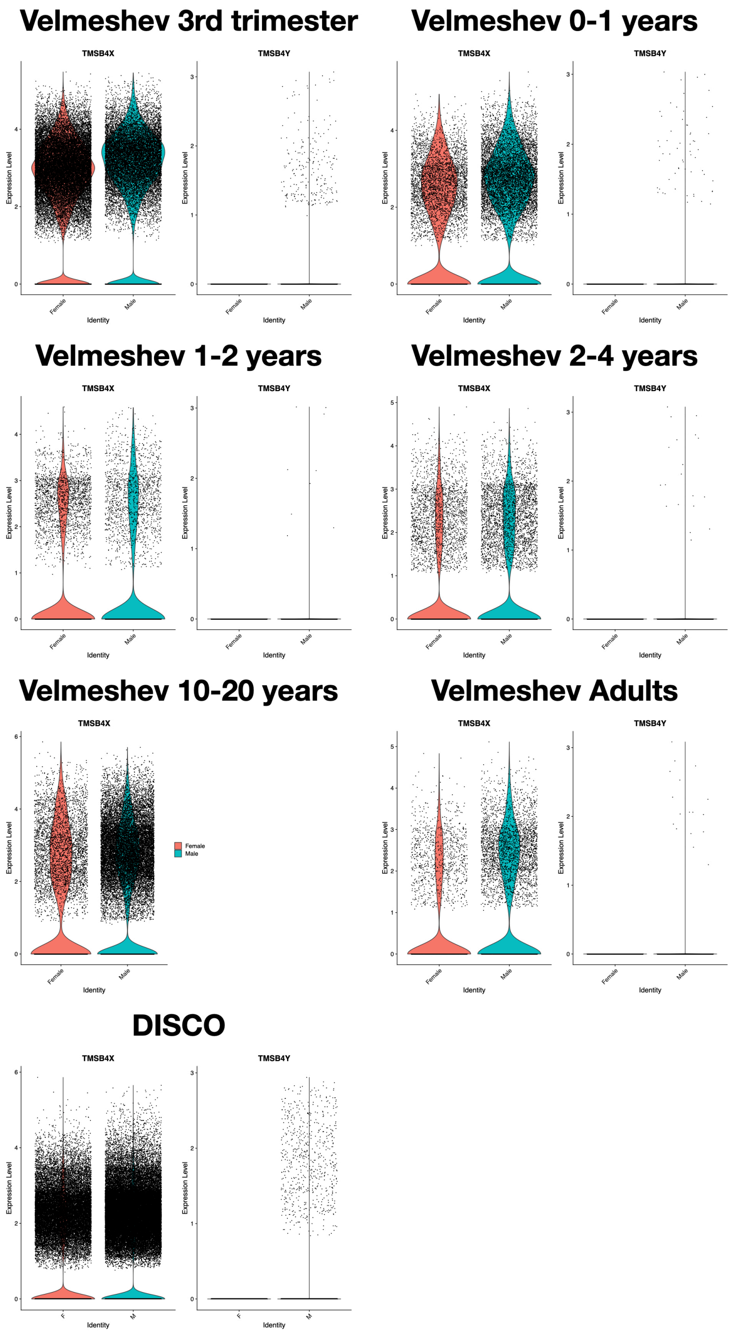
Velmeshev 3rd trimester
Velmeshev 0-1 years
Velmeshev 1-2 years
Velmeshev 2-4 years
Velmeshev 10-20 years
Velmeshev Adults
DISCO
TMSB4X
TMSB4Y
Expression Level
Identity
Female
Male
F
M

Fig. S53: **Expression of TMSB4X and TMSB4Y in the sexes in each dataset.** The X- and Y-linked genes were expressed in almost every dataset, indicating that the biased expression we observed for TMSB4X is due to actual differential expression and not other causes, e.g. misalignment. As seen previously, the second trimester did not have X and Y chromosome genes, therefore is missing.

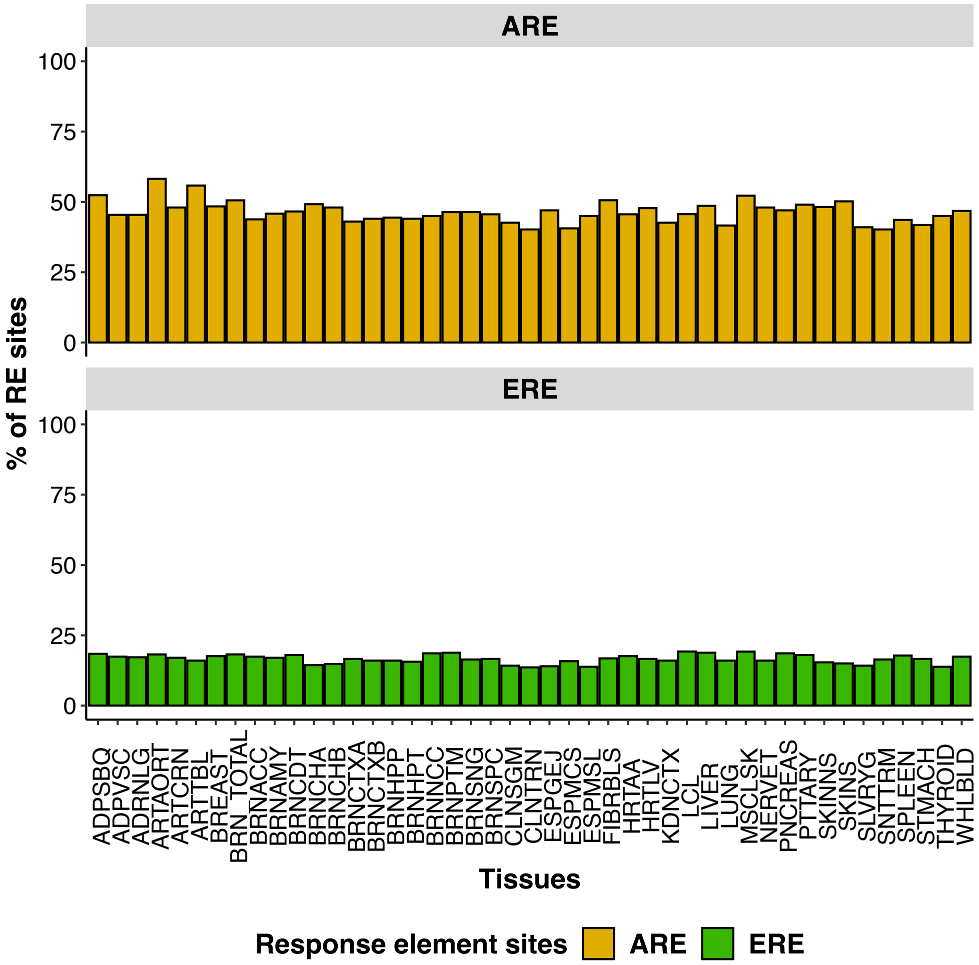

Fig. S54: **Percentage of the top 500 human organ-specific genes which express sex hormones response elements.** The organ-specific genes were calculated and ranked according to local false sign rate previously (Oliva et al., 2020). For each of the organs/tissues, we calculated how many genes were presenting AREs (top) and EREs (bottom). No modifications were performed on the groups or the labeling from the original study, except for the introduction of 'BRN_TOTAL', in which we grouped together the distinct genes from all brain regions combined, to investigate whether we could a overall brain-specificity. **Abbreviations:** ARE: androgen response element; ERE: estrogen response element; ADPSBQ: Subcutaneous adipose; ADPVSC: Visceral omentum; ADRNLG: Adrenal gland; ARTAORT: Aorta; ARTCRN: Coronary artery; ARTTBL: Tibial artery; BREAST: Breast mammary tissue; BRN_TOTAL: All brain regions; BRNACC: Anterior cingulate cortex; BRNAMY: Amygdala; BRNCDT: Caudate [basal ganglia]; BRNCHA/BRNCHB: Cerebellum/Cerebellar hemisphere; BRNCTXA/BRNCTXB: Cortex/Frontal cortex; BRNHPP: Hippocampus; BRNHPT: Hypothalamus; BRNNCC: Nucleus accumbens [basal ganglia]; BRNPTM: Putamen [basal ganglia]; BRNSNG: Substantia nigra; BRNSPC: Spinal cord [cervical c-1]; CLNSGM: Sigmoid colon; CLNTRN: Transverse colon; ESPGEJ: Gastroesophageal junction; ESPMCS: Esophagus mucosa; ESPMSL: Esophagus muscularis; FIBRBLS: Cultured fibroblasts; HRTAA: Atrial appendage; HRTLV: Left ventricle; KDNCTX: Kidney cortex; LCL: lymphoblastoid cell lines; LIVER: Liver; LUNG: Lung; MSCLSK: Skeletal muscle; NERVET: Tibial nerve; PNCREAS: Pancreas; PTTARY: Pituitary; SKINNS: Not sun-exposed skin (suprapubic); SKNS: Sun-exposed skin (lower leg); SLVRYG: Minor salivary gland; SNTTRM: Small intestine terminal ileum; SPLEEN: Spleen; STMACH: Stomach; THYROID: Thyroid; WHLBLD: Whole blood.

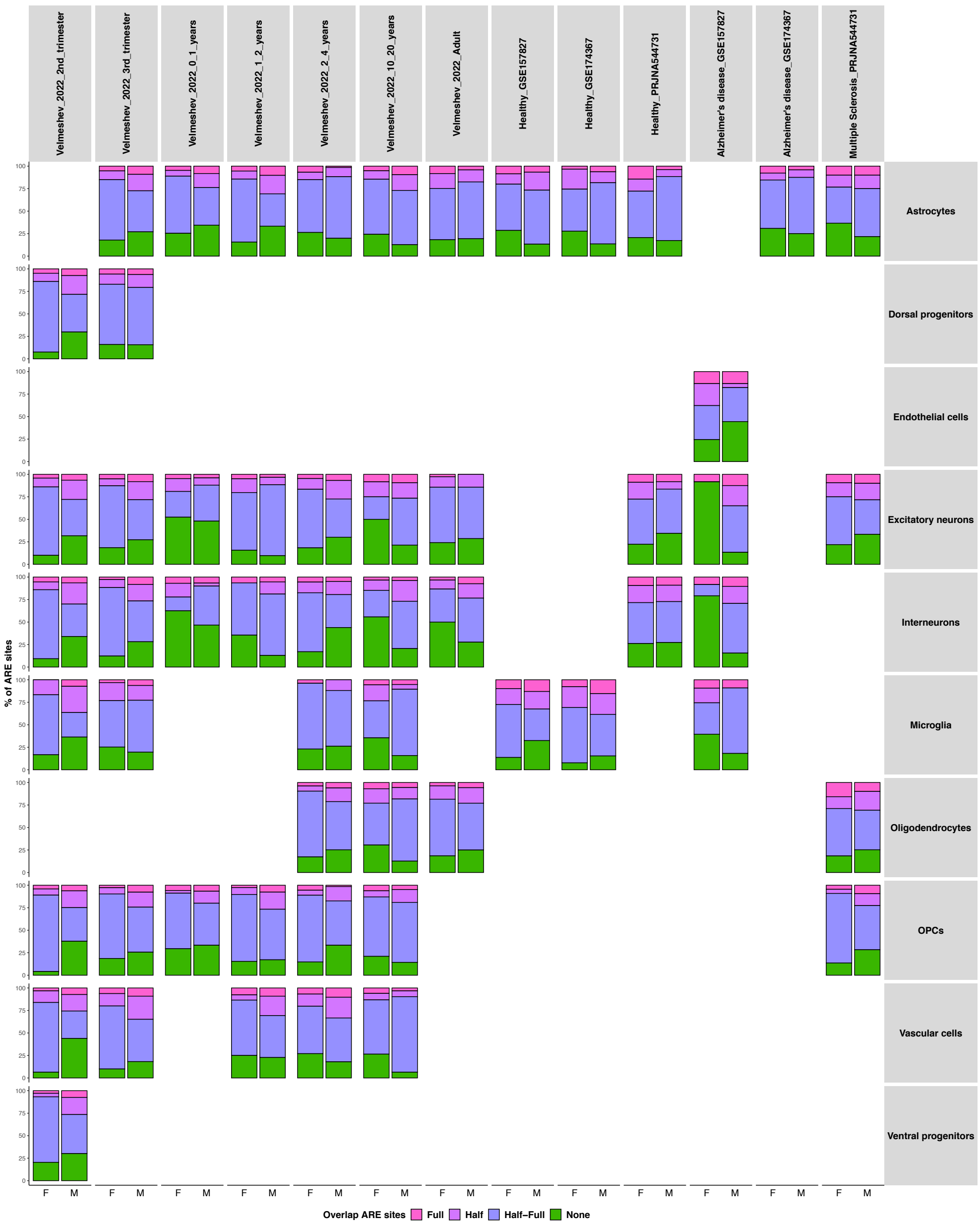
Velmeshev_2022_2nd_trimester
Velmeshev_2022_3rd_trimester
Velmeshev_2022_0_1_years
Velmeshev_2022_1_2_years
Velmeshev_2022_2_4_years
Velmeshev_2022_10_20_years
Velmeshev_2022_Adult
Healthy_GSE157827
Healthy_GSE174367
Healthy_PRJNA544731
Alzheimer's disease_GSE157827
Alzheimer's disease_GSE174367
Multiple Sclerosis_PRJNA544731
Astrocytes
Dorsal progenitors
Endothelial cells
Excitatory neurons
Interneurons
Microglia
Oligodendrocytes
OPCs
Vascular cells
Ventral progenitors
% of ARE sites
0
25
50
75
100
F
M
Overlap ARE sites
Full
Half
Half–Full
None

Fig. S55: **Androgen response element (ARE) sites are strongly present across cell types, datasets and sexes.** These bar plots show the ARE sites expression in the SG-biased genes, divided in full, half and full-half sites. ARE sites percentages were overall around 75%, much higher than expected, with few exceptions (e.g. female-biased genes in the neuronal populations in the AD GSE157827 dataset). The majority of SG-biased genes had both half and full sites for AREs. **Abbreviations:** ARE: androgen response element.

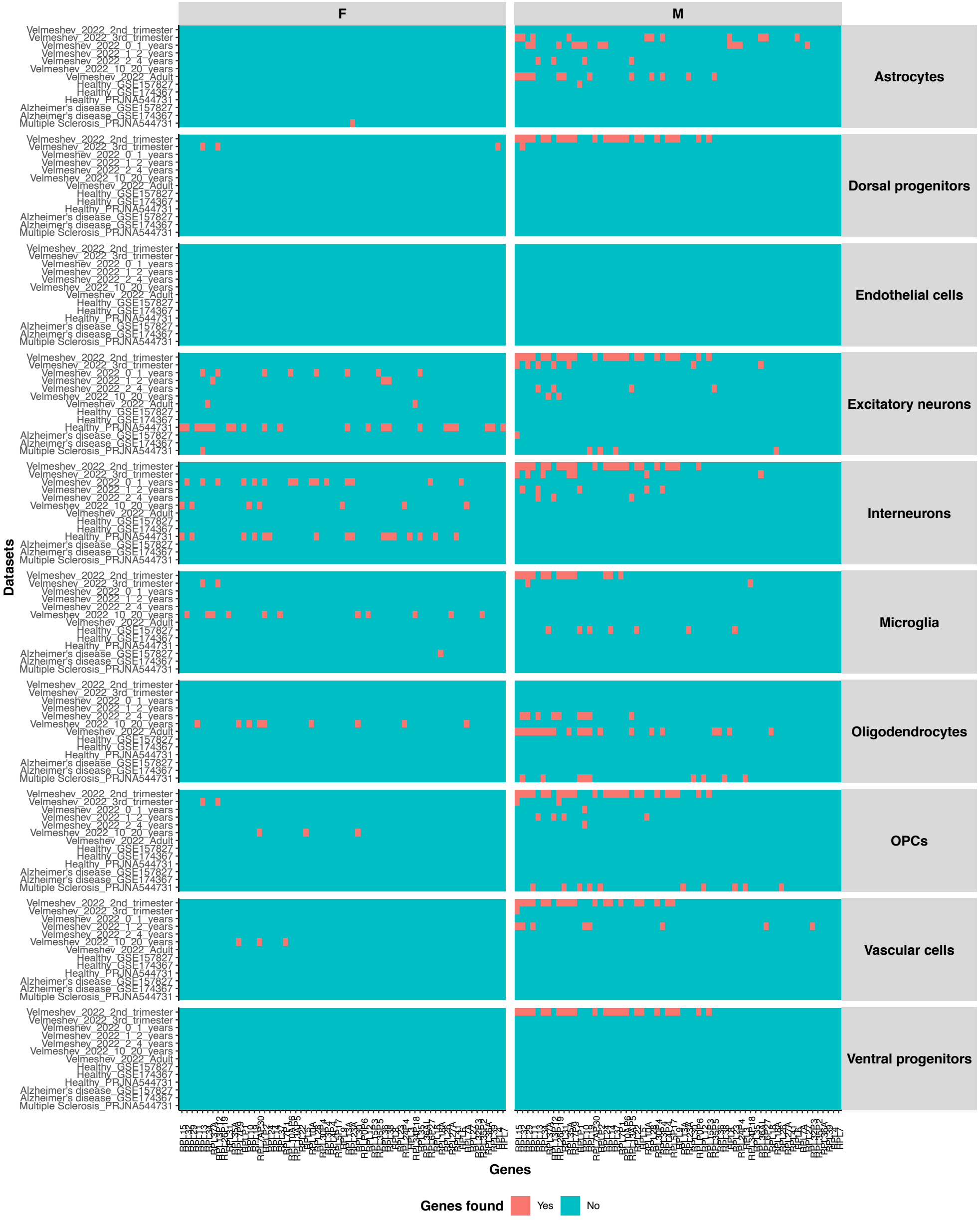

Fig. S56: **Presence of L ribosomal proteins (RPL) genes in the SG-biased genes.** Heatmap showing the presence of RPL genes across cell types and sex within each dataset. Presence indicates whether the gene is found in each specific dataset-sex-cell type combination.

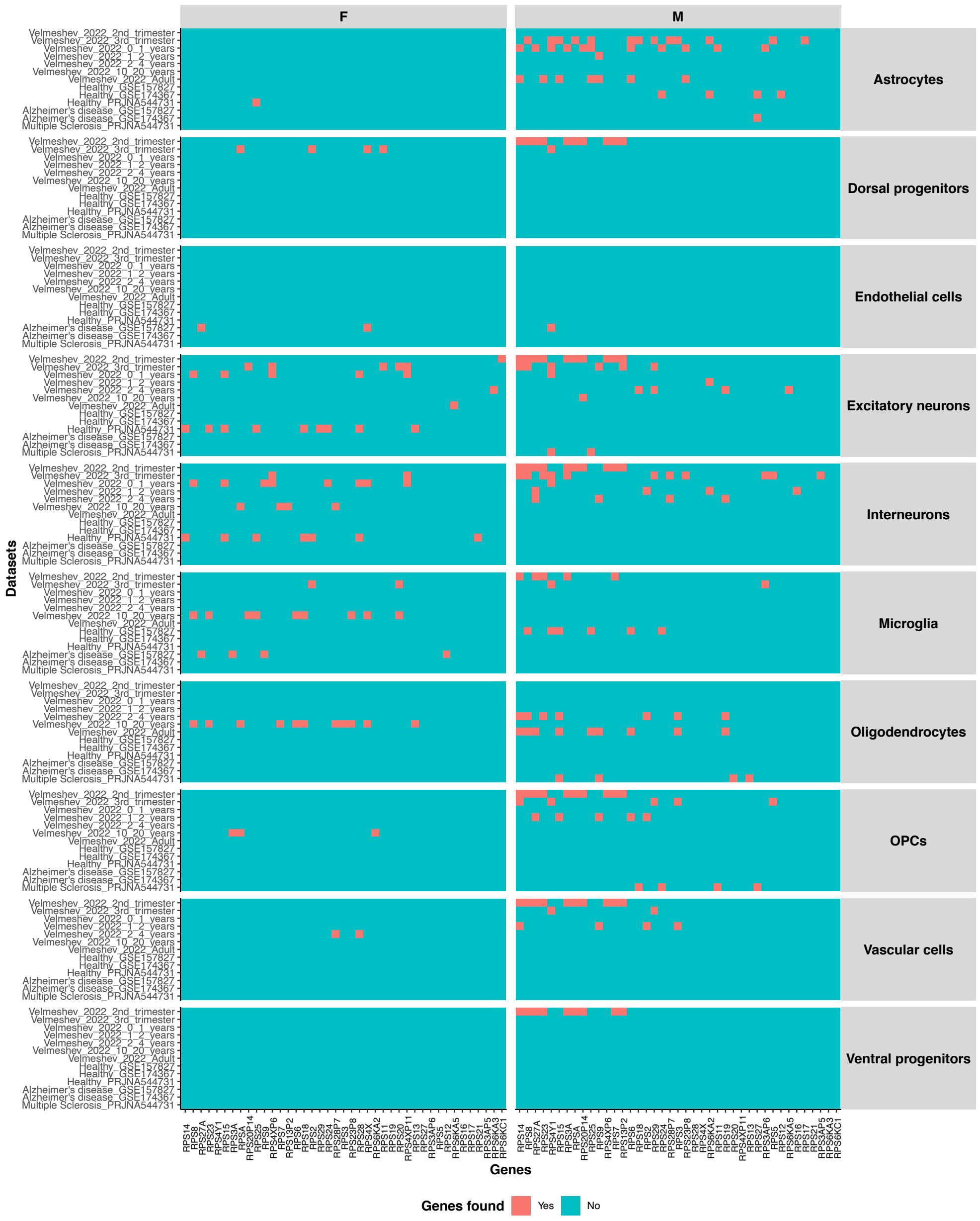

Fig. S57: **Presence of S ribosomal proteins (RPS) genes in the SG-biased genes.** Heatmap showing the presence of RPS genes across cell types and sex within each dataset. Presence indicates whether the gene is found in each specific dataset-sex-cell type combination.

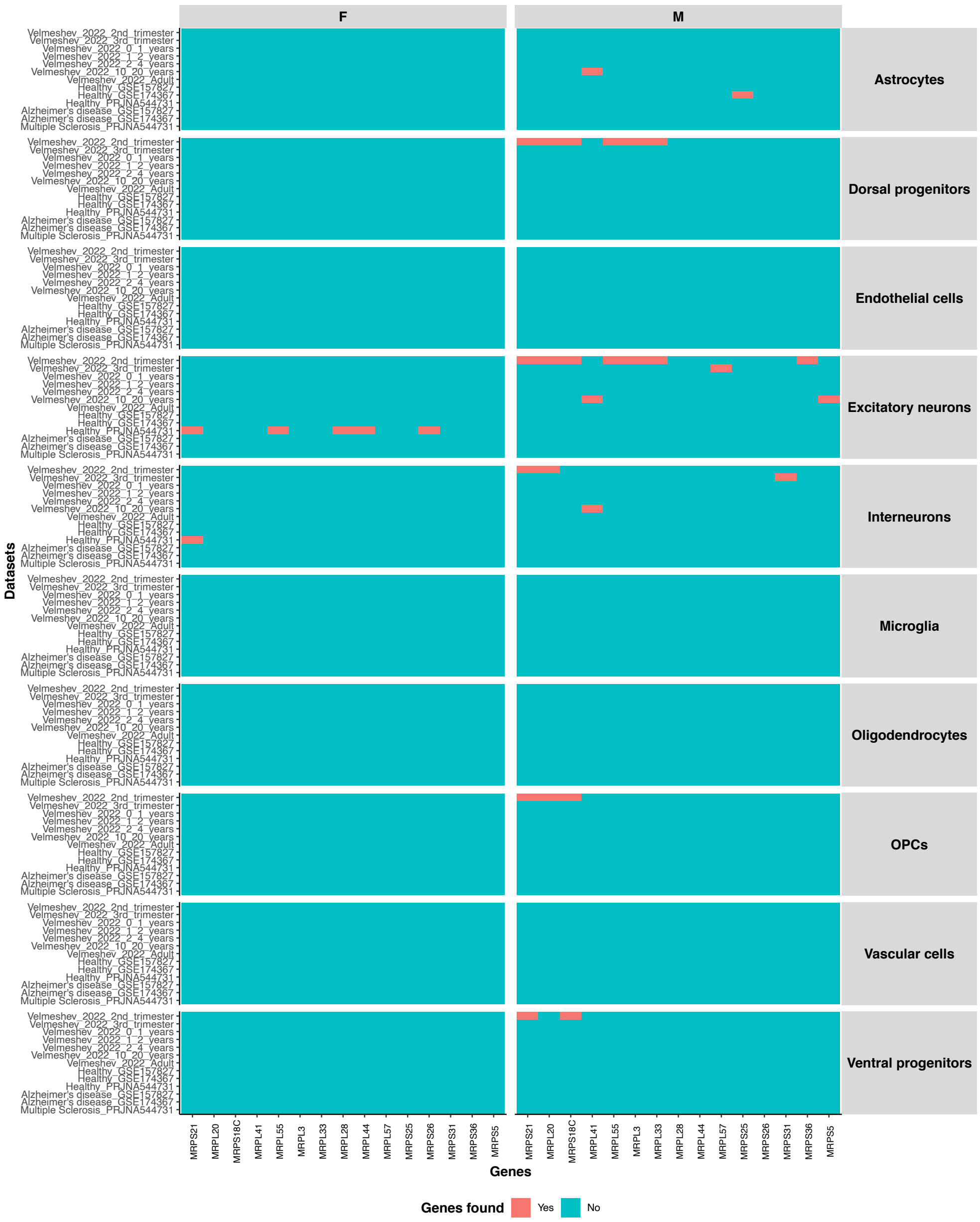

Fig. S58: **Presence of mitochondrial ribosomal proteins (MRP) genes in the SG-biased genes.** Heatmap showing the presence of MRP genes across cell types and sex within each dataset. Presence indicates whether the gene is found in each specific dataset-sex-cell type combination.
